# Small molecules in targeted cancer therapy: advances, challenges, and future perspectives

**DOI:** 10.1038/s41392-021-00572-w

**Published:** 2021-05-31

**Authors:** Lei Zhong, Yueshan Li, Liang Xiong, Wenjing Wang, Ming Wu, Ting Yuan, Wei Yang, Chenyu Tian, Zhuang Miao, Tianqi Wang, Shengyong Yang

**Affiliations:** 1 State Key Laboratory of Biotherapy and Cancer Center, West China Hospital, Sichuan University, Chengdu, Sichuan People’s Republic of China; 2 Personalized Drug Therapy Key Laboratory of Sichuan Province, Department of Pharmacy, Sichuan Provincial People’s Hospital, School of Medicine, University of Electronic Science and Technology of China, Chengdu, Sichuan People’s Republic of China

**Keywords:** Drug development, Drug discovery

## Abstract

Due to the advantages in efficacy and safety compared with traditional chemotherapy drugs, targeted therapeutic drugs have become mainstream cancer treatments. Since the first tyrosine kinase inhibitor imatinib was approved to enter the market by the US Food and Drug Administration (FDA) in 2001, an increasing number of small-molecule targeted drugs have been developed for the treatment of malignancies. By December 2020, 89 small-molecule targeted antitumor drugs have been approved by the US FDA and the National Medical Products Administration (NMPA) of China. Despite great progress, small-molecule targeted anti-cancer drugs still face many challenges, such as a low response rate and drug resistance. To better promote the development of targeted anti-cancer drugs, we conducted a comprehensive review of small-molecule targeted anti-cancer drugs according to the target classification. We present all the approved drugs as well as important drug candidates in clinical trials for each target, discuss the current challenges, and provide insights and perspectives for the research and development of anti-cancer drugs.

## Introduction

Drug treatment together with surgical operation, radiotherapy and biotherapy constitute the main approaches to cancer treatment. For a long time, chemotherapy, which is a method of killing tumor cells and/or inhibiting the growth and proliferation of tumor cells by chemical drugs, was the only approach to cancer drug therapy. The biggest characteristic of chemotherapy is the inability to distinguish between cancer cells and normal cells, resulting in significant toxicity and side effects. Over the past two decades, there has been a tremendous shift in cancer treatment, from broad-spectrum cytotoxic drugs to targeted drugs.^1^ Compared with traditional chemotherapy drugs, targeted drugs can specifically target cancer cells but spare normal cells, hence having high potency and low toxicity. Encouraged by the approval of the first small-molecule tyrosine kinase inhibitor (TKI) imatinib for clinical use by the US Food and Drug Administration (FDA) in 2001,^2^ targeted drugs have rapidly developed and entered a golden period of development. In the past 20 years, there has been a significant increase in FDA-approved targeted drugs for cancer treatment.

**Fig. 1:**
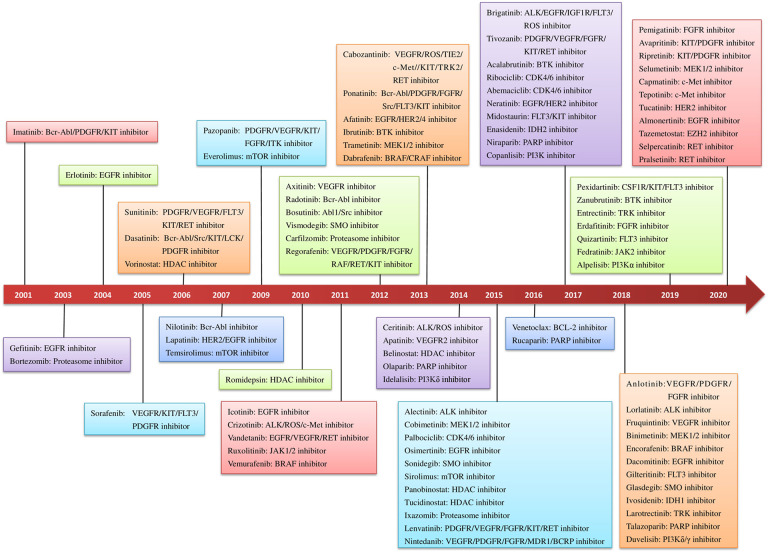
Timeline for the approval of small-molecule targeted anti-cancer drugs

Targeted drugs can be roughly classified into two categories: small molecules and macromolecules (e.g., monoclonal antibodies, polypeptides, antibody–drug conjugates, and nucleic acids).^3,4^ Compared with macromolecule drugs, small-molecule targeted drugs have advantages in some aspects such as the pharmacokinetic (PK) properties, costs, patient compliance, and drug storage and transportation (Supplementary Table S1). Despite challenged by macromolecule drugs represented by monoclonal antibodies in recent years, small-molecule targeted drugs still gain great development. To date, there are a total of 89 anti-cancer small molecules approved in the United States and China. Figure 1 summarizes the small-molecule anti-cancer drugs approved by the US FDA and National Medical Products Administration (NMPA) of China since 2001. The targets of these drugs cover a large scope including kinases, epigenetic regulatory proteins, DNA damage repair enzymes, and proteasomes. It is undeniable that small-molecule targeted anti-cancer drugs still face many challenges such as low response rate and drug resistance.

To better promote the development of small-molecule targeted anti-cancer drugs, we will conduct a comprehensive review for them. In order to facilitate the description, protein targets of the approved agents will be taken as a clue. For each target, marketed small-molecule drugs and important drug candidates in clinical trials will be presented. Finally, an analysis of the current challenges in the field and a future perspective will also be given.

## Kinase inhibitors

**Fig. 2:**
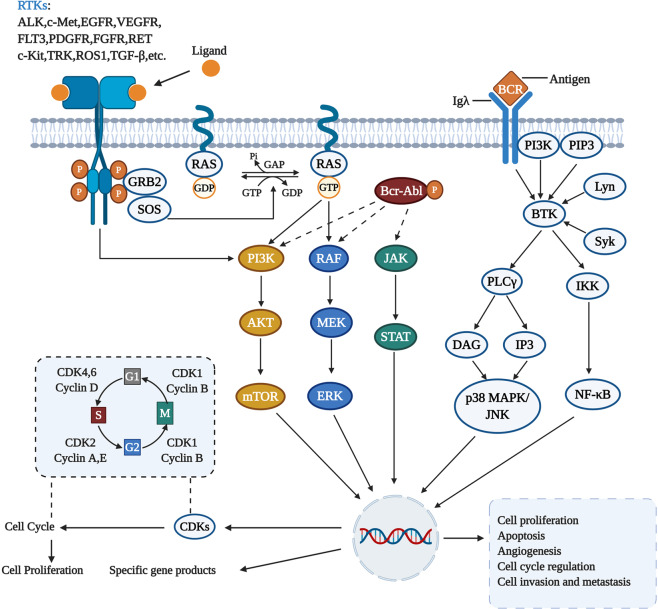
Activation of different protein kinase-dependent pathways. The set of RTKs influences a small number of intermediaries, such as phosphoinositide 3-kinase (PI3K) and mitogen-activated protein kinases (MAPK), thereby activating the complex signaling networks that are related to cell proliferation, differentiation, adhesion, apoptosis, and migration. The aggregation, activation, and depolymerization of the periodic CDK-cyclin complex are critical events driving cell cycle turnover. Figure created with BioRender.com

Protein kinase is a kind of enzyme that catalyzes the transfer of γ-phosphate group from ATP to protein residues containing hydroxyl groups. It has an important role in cell growth, proliferation, and differentiation (Fig. 2).^5^ The human kinome comprises ~535 protein kinases.^6^ According to the substrate residues, protein kinases can be classified as tyrosine kinases (including both receptor and non-receptor tyrosine kinases), serine/threonine kinases, and tyrosine kinase-like enzymes. Dysregulation of protein kinases is linked to various diseases, particularly cancer. Protein kinases are the most widely studied tumor therapeutic targets. Currently, a large number of protein kinase inhibitors have been reported. These kinase inhibitors can be classified into different categories by using many ways. Here we adopted an integrated classification system proposed by Roskoski, which is one of the most widely used methods.^7^ According to this classification system, protein kinase inhibitors are classified into six types (Type-I–VI). Type-I inhibitors bind to the active conformation of the kinase (DFG-Asp in, αC-helix in). Type-I½ inhibitors bind to a DFG-Asp in inactive kinase conformation with αC-helix out, while type-II inhibitors bind to a DFG-Asp out inactive conformation. These types of inhibitors occupy part of the adenine binding pocket and form hydrogen bonds with the hinge region connecting the small and large lobes of the enzyme. Among them, type-I½ and type-II antagonists can be further divided into A and B subtypes. Type A inhibitors extend past the Sh2 gatekeeper residue into the back cleft, while type B inhibitors fail to extend into the back cleft. The possible importance of this difference is that type A inhibitors have longer residence times compared with type B inhibitors when binding to their targets. Type III and type IV kinase inhibitors are allosteric in nature. Type III inhibitors restrain kinase activity by binding to an allosteric site, which is in the cleft between the small and large kinase lobes adjacent to the ATP-binding pocket. Contrariwise, type IV inhibitors bind outside of the cleft. Moreover, the bivalent molecules that span two distinct regions of the kinase domain are type V inhibitors. Type-I–V inhibitors are all reversible. In contrast, compounds that bind covalently with the kinase active site are called type VI inhibitors (irreversible kinase inhibitors).

## Receptor tyrosine kinase inhibitors

### ALK inhibitors

Anaplastic lymphoma kinase (ALK) encoded by the *ALK* gene is a single transmembrane tyrosine kinase of the insulin receptor family.^8^ ALK can activate multiple downstream signaling pathways and has an important role in the development of the nervous system.^9^ Constitutive activation of ALK through point mutations or chromosomal rearrangements has been identified in multiple human cancers such as anaplastic large cell lymphoma, diffuse large B-cell lymphoma (DLBCL),^10^ inflammatory myofibroblastic tumor,^11^ and non-small cell lung cancer (NSCLC).^12^ Fusion of echinoderm microtubule-associated protein-like 4 with *ALK* (*EML4-ALK*) in NSCLC was identified in 2007 by Soda et al.^12^ this rearrangement of the *ALK* gene has been detected in ~3–7% of patients with NSCLC. *EML4-ALK* gene fusion is initiated by inversion in the short arm of chromosome 2, which juxtaposes the N-terminal of the *EML4* promoter and the kinase domain of the *ALK* gene, ultimately leading to ligand-independent constitutive activation of ALK and promoting cancer cell proliferation and survival. Several other *ALK* gene fusions, such as *NPM-ALK*, *ATIC-ALK*, and *RANBP2-ALK*, have also been discovered;^13–15^ these rearrangements define a specific subgroup of cancerous patients that can be treated with selective ALK inhibitors.^16^

Crizotinib approved in 2011 is a first-generation ALK inhibitor targeting multiple tyrosine kinases including ALK, cellular-mesenchymal-epithelial transition factor (c-Met), and proto-oncogene tyrosine-protein kinase reactive oxygen species (ROS) (Table 1).^17^ Two randomized phase III trials (NCT00932893, NCT01154140) established the superiority of crizotinib over chemotherapy in patients with advanced *ALK*-rearranged NSCLC, and it is now the standard drug therapy for metastatic *ALK*-positive NSCLC.^18,19^ Unfortunately, most patients develop resistant mutations to crizotinib within 12 months, especially L1196M and G1269A mutations, which can lead to relapse.^20^ The central nervous system (CNS) is the most common relapse site in patients with NSCLC treated with crizotinib, probably because of its poor blood–brain barrier (BBB) permeability.^21^ The second-generation ALK inhibitors ceritinib,^22^ alectinib,^23^ and brigatinib^24^ were subsequently developed for the treatment of crizotinib-resistant *ALK*-positive NSCLC, all of which are multikinase inhibitors (Table 1). Ceritinib is more potent than crizotinib and has doubled progression-free survival (PFS) compared with chemotherapy in clinical studies.^22^ Alectinib has advantages over both crizotinib and ceritinib, and has shown inhibitory activity against several crizotinib or ceritinib-resistant ALK mutations such as L1196M, G1269A, C1156Y, and F1174L.^25^ This agent is not a substrate of P-glycoprotein and can cross the BBB and effectively prevent the progression of CNS metastases. It was approved for NSCLC treatment in 2015 and recommended as first-line therapy for patients with *ALK* fusion-positive NSCLC in 2017. Moreover, brigatinib was granted accelerated approval by the FDA in 2017 as second-line therapy for patients with *ALK*-positive metastatic NSCLC, based on the considerable systemic and intracranial responses in clinical trials.^26^ Similar to the experience with crizotinib, novel resistance mechanisms were observed in patients who relapsed after treatment with second-generation ALK inhibitors. Secondary ALK kinase domain mutations, such as the G1202R, V1180L, and I1171T mutants, are the most common resistance mechanisms.^27^ Lorlatinib is an oral ATP-competitive brain penetrant inhibitor of ALK/ROS1 approved in 2018.^28,29^ As a third-generation ALK inhibitor, all recognized ALK mutations (except L1198F mutation) can be targeted by lorlatinib.^29^ Interestingly, lorlatinib is structurally distinct from most second-generation ALK inhibitors but has the same structural basis as crizotinib. However, patients harboring the L1198F mutation, which confers resistance to lorlatinib, have reported re-sensitivity to crizotinib.^30^ This result indicates that retreatment under molecular guidance should be considered as a clinically meaningful approach for *ALK*-positive NSCLC.

**Table 1:** Properties of approved small-molecule inhibitors of receptor tyrosine kinases

Chemical structure	Name	Targets	Approved indications (year)	Corporation
	Crizotinib (Xalkori)	ALK/ROS/c-Met	NSCLC (2011)	Pfizer
	Ceritinib (Zykadia)	ALK/ROS	NSCLC (2014)	Novartis
	Alectinib (Alecensa)	ALK	NSCLC (2015)	Roche/Chugai
	Brigatinib (Alunbrig)	ALK/ROS/ IGF1R/EGFR/FLT3	NSCLC (2017)	Ariad
	Lorlatinib (Lorbrena)	ALK	NSCLC (2018)	Pfizer
	Capmatinib (Tabrecta)	c-Met	NSCLC (2020)	Novartis
	Tepotinib (Tepmetko)	c-Met	NSCLC (2020)	Merck
	Gefitinib (Iressa)	EGFR	NSCLC (2003)	AstraZeneca
	Erlotinib (Tarceva)	EGFR	NSCLC (2004) Pancreatic cancer (2005)	Roche/Astellas
	Lapatinib (Tykerb)	EGFR/HER2	Breast cancer (2007)	Novartis
	Icotinib (Conmana)	EGFR	NSCLC (2011)	Betta
	Afatinib (Gilotrif)	EGFR/HER2/HER4	NSCLC (2013)	Boehringer Ingelheim
	Osimertinib (Tagrisso)	EGFR	NSCLC (2015)	AstraZeneca
	Neratinib (Nerlynx)	HER2/HER4/EGFR	Breast cancer (2017)	Puma Biotech
	Dacomitinib (Vizimpro)	EGFR	NSCLC (2018)	Pfizer
	Almonertinib (Ameile)	EGFR	NSCLC (2020)	Hansoh
	Tucatinib (Tukysa)	HER2	Breast cancer (2020)	Seattle Genetics
	Midostaurin (Rydapt)	FLT3/c-Kit	AML (2017)	Novartis
	Gilteritinib (Xospata)	FLT3/AXL	AML (2018)	Kotobuki/Astellas
	Quizartinib (Vanflyta)	FLT3	AML (2019)	Daiichi Sankyo
	Pexidartinib (Turalio)	CSF1R/c-Kit/FLT3	TGCT (2019)	Daiichi Sankyo
	Sorafenib (Nexavar)	c-Kit/FLT3/RET/PTC/ VEGFR-1/2/3/PDGFR-β	RCC (2005) HCC (2007) DTC (2013) Thyroid cancer (2014)	Bayer
	Sunitinib (Sutent)	PDGFR-α/β/VEGFR-1/2/3/CSF1R/c-Kit/RET/FLT3	RCC (2006) GIST (2006) Pancreas neuroendocrine tumor (2011)	Pfizer
	Pazopanib (Votrient)	PDGFR-β/VEGFR-1/2/3/ FGFR-1/3/c-Kit/Itk/Lck/c-GSK	RCC (2009) STS (2012)	Novartis
	Vandetanib (Caprelsa)	EGFR/VEGFR/RET/BRK/TIE2/EPH	MTC (2011)	Genzyme
	Axitinib (Inlyta)	VEGFR-1/2/3	RCC (2012)	Pfizer
	Cabozantinib (Cometriq)	VEGFR-1/2/3/TYRO3/ROS/TIE2/c-Met/HGFR/c-Kit/TRK2/RET	MTC (2013) RCC (2016) HCC (2019)	Exelixis
	Regorafenib (Stivarga)	VEGFR-1/2/3/PDGFR-α/β/FGFR-1/2/RAF/RET/c-Kit	CRC (2012) GIST (2013) HCC (2017)	Bayer
	Apatinib (Aitan)	VEGFR-2/Src/c-Kit	Gastric cancer (2014)	Hengrui Medicine
	Lenvatinib (Lenvima)	PDGFR-α/VEGFR-1/2/3/FGFR-1/2/3/4/c-Kit/RET	DTC (2015) Thyroid cancer (2015) RCC (2016) HCC (2018) Endometrial carcinoma (2019)	Eisai
	Tivozanib (Fotivda)	PDGFR-α/VEGFR-1/2/3/FGFR-1/2/3/4/c-Kit/RET	RCC (2017)	Eusa
	Fruquintinib (Elunate)	VEGFR-1/2/3	CRC (2018)	Chi-Med/Lilly
	Nintedanib (Ofev)	VEGFR-1/2/3/PDGFR-α/β/ MDR1/BCRP/FGFR-1/3	NSCLC (2015)	Boehringer Ingelheim
	Anlotinib (Focus V)	VEGFR-2/3/PDGFR-β/FGFRs	NSCLC (2018) STS (2019) SCLC (2020)	Chia Tai Tianqing
	Erdafitinib (Balversa)	FGFR-1/2/3/4	Urothelial carcinoma (2019)	Janssen
	Pemigatinib (Pemazyre)	FGFR-1/2/3/4	Cholangiocarcinoma (2020)	Incyte
	Avapritinib (Ayvakit)	c-Kit/PDGFR-α	GIST (2020)	Blueprint
	Ripretinib (Qinlock)	c-Kit/PDGFR-α	GIST (2020)	Deciphera
	Selpercatinib (Retevmo)	RET	NSCLC (2020) MTC (2020) Thyroid cancer (2020)	Loxo
	Pralsetinib (Gavreto)	RET	NSCLC (2020) MTC (2020) Thyroid cancer (2020)	Blueprint Medicines
	Larotrectinib (Vitrakvi)	TRKA/B/C	Solid tumors with *NTRK* fusion (2018)	Bayer
	Entrectinib (Rozlytrek)	TRKA/B/C/ROS1/ALK	Solid tumors with *NTRK* fusion (2019)	Roche

Currently, there are still some ALK inhibitors under clinical investigation, such as the pan-TKIs entrectinib,^31^ belizatinib,^32^ and repotrectinib,^33^ which target oncogenic rearrangements in ALK, ROS, and tropomyosin receptor kinase (TRK).^34^ Among them, entrectinib was approved for the treatment of TRK fusion solid tumors in 2019. The clinical use of these drugs as ALK inhibitors is still under evaluation.^31^ The aminopyrazine derivative ensartinib is a newly developed second-generation ALK inhibitor. It exhibits efficacy in crizotinib-naive and crizotinib-resistant patients with *ALK*-positive, locally advanced or metastatic NSCLC, as well as patients with brain metastases.^34^ A phase III study is ongoing to compare ensartinib with crizotinib for the first-line treatment of *ALK*-positive NSCLC (NCT02767804). Moreover, CEP-37440 is an orally administered inhibitor of ALK and focal adhesion kinase. A phase I trial (NCT01922752) of this agent has been performed in patients with solid tumors, but no preliminary data are available.^35^

Drug-resistant mutations are major obstacles that limit the clinical efficacy of ALK inhibitors. To date, ALK inhibitors have been developed to the third generation. Rational sequential therapy (using first-, second-, and third-generation ALK inhibitors for NSCLC therapy sequentially according to *ALK* gene mutations) can effectively overcome drug resistance and improve the survival of applicable patients. Of note is that retreatment with crizotinib can benefit patients harboring the ALK L1198F mutation, which is resistant to the latest generation of ALK inhibitors.^36^ For resistance arising from bypass activation, combining ALK inhibitors with other targeted therapies such as a mitogen-activated protein kinase (MAPK) inhibitor,^37,38^ cyclin-dependent kinase (CDK) inhibitor (ceritinib with ribociclib, NCT02292550), mammalian target of rapamycin (mTOR) inhibitor (ceritinib with everolimus, NCT02321501), and heat-shock protein 90 inhibitor has been assessed in a number of trials.^39^ Since the expression of programmed death-ligand 1 is reportedly associated with *EML4-ALK*, combined treatments of ALK and immune checkpoint inhibitors have also been evaluated in *ALK*-positive NSCLC.^38^ In addition to kinase inhibitors, degrading carcinogenic proteins using proteolysis targeting chimera (PROTAC) technology is an effective anti-cancer strategy. The PROTACs MS4077 and MS4078 designed by Zhang et al. have shown great potency in reducing ALK fusion protein in preclinical studies, suggesting a new approach to drug discovery targeting ALK.^40^

### c-Met inhibitors

Cellular-mesenchymal-epithelial transition factor (c-Met), also known as hepatocyte growth factor receptor (HGFR), is encoded by the *MET* proto-oncogene located on chromosome 7q21-31.^41,42^ Under normal physiology, the binding of c-Met to its sole ligand HGF initiates the activation of the HGF/c-Met signaling pathway, which further activates several downstream signals including the PI3K/AKT, MAPK, STAT, and NF-κB pathways, and has a central role in a variety of cytoplasmic and nuclear processes, such as cell proliferation, survival, invasion, motility, scattering, angiogenesis, and epidermal–mesenchymal transition.^42–44^ These normal regulatory functions mainly occur during embryonic development, wound healing, and post-injury tissue regeneration. However, aberrant activation of c-Met signaling caused by *MET* amplification, mutation, inadequate degradation, transcriptional deregulation, or aberrant HGF autocrine or paracrine has been implicated in the development of various solid tumors.^44,45^ c-Met overexpression has also been reported to be related to poor prognosis and resistance to cytotoxic and molecular targeted therapy, especially for patients treated with EGFR inhibitors, in which *MET* amplification accounts for ~20% of resistant cases.^46^ Activating *MET* mutations usually occur in the semaphoring domain (e.g., E168D) and juxtamembrane domain (e.g., T1010I, P1009S, skipping mutation) of exons 14, 18, and 19. Of these, c-Met exon 14 skipping mutations promote its oncogenic activity by suppressing c-Met receptor degradation.^43,45,47^ These mutations are rare in patients with primary tumors but common in advanced cancers with metastases, especially in lung adenocarcinoma, brain gliomas, and renal cell carcinoma (RCC).^47,48^

During the last decade, great progress has been made in antitumor therapy targeting the HGF/c-Met signaling pathway. The early developed c-Met inhibitors were multikinase inhibitors. As early as 2011 and 2012, two multitarget c-Met inhibitors, crizotinib and cabozantinib, were approved for the treatment of NSCLC and medullary thyroid cancer (MTC) as well as RCC, respectively.^49,50^ However, the indications are not based on their ability to target c-Met but are due to the inhibitory effect of crizotinib on the ALK fusion protein and the multikinase inhibitory activity of cabozantinib. The development of selective c-Met inhibitors has progressed rapidly in recent years, and two highly selective c-Met inhibitors, capmatinib and tepotinib, were approved in the first half of 2020 (Table 1). Capmatinib (INCB28060) is an oral competitive c-Met inhibitor with ≥10,000-fold selectivity for c-Met compared with other kinases and potently inhibits c-Met activity at picomolar concentrations.^51^ In the GEOMETRY *mono-1* trial (NCT02414139) conducted in patients with *MET* exon 14 skipping mutations, capmatinib exhibited a high objective response rate (ORR) and relatively durable responses in both previously treated and newly diagnosed patients, including those with brain metastases.^52^ Combination therapy of capmatinib and gefitinib was also evaluated in a phase II trial in NSCLC patients with disease progression after gefitinib treatment (NCT01610336). A disease control rate of 80% was achieved in 65 subjects, and more responses were observed in patients with *MET* amplification.^53^ Similar results were reported in the combination therapy of capmatinib with other EGFR inhibitors, such as erlotinib.^54^ Due to its significant efficacy compared to existing therapies, capmatinib granted a breakthrough therapy designation by the FDA for NSCLC patients harboring *MET* exon 14 skipping mutations in 2019 and was approved for this indication on May 6, 2020. Tepotinib (EMD1214063), developed by Merck, has more than 1000-fold selectivity for c-Met.^55^ Clinical trials of tepotinib (NCT04647838 and NCT03940703) also showed significant effectiveness in the treatment of cancer patients harboring *MET* mutations and in combination therapy with EGFR TKIs.^56^ It has been approved by the Ministry of Health, Labour and Welfare (MHLW) of Japan for the treatment of unresectable, advanced, or recurrent NSCLC in patients with skipping mutations in *MET* exon 14.^57^

There are also many small-molecule c-Met inhibitors at different stages of clinical trials. Representative multikinase c-Met inhibitors include foretinib (XL880/GSK1363089), glesatinib (MGCD265), BMS-777607, and S49076, which target c-Met/RON/VEGFR-2/KIT/TIE2/PDGFR, c-Met/TIE2/RON/VEGFR-1/2/3, c-Met/RON/AXL, and c-Met/AXL/MER/FGFR, respectively.^58–61^ Several clinical trials were carried out to test their efficacy for cancer therapy, but some of the results have not been disclosed. In a phase I trial for NSCLC patients who progressed after chemotherapy (NCT01068587), combined foretinib with erlotinib could achieve a response rate of 17.8% in the evaluated patients, and the clinical response was closely associated with baseline c-Met expression.^58^ In a phase I trial conducted in patients with advanced solid tumors (ISRCTN00759419), S49076 was administered orally once daily or twice daily in continuous 21-day cycles at escalating doses, followed by an expansion phase at the RP2D. The results showed that 83 patients (81.4%) had side effects, and 93% of them were grade 1–2. Nine patients had more than 6 months of stable disease, and the overall clinical benefit rate was 23%.^61^ In addition, several c-Met-specific inhibitors are also undergoing clinical research. Tivantinib is a non-ATP-competitive inhibitor of c-Met. In phase II randomized open-label study conducted in previously treated locally advanced or metastatic NSCLC patients (NCT00777309), the combination of tivantinib and erlotinib showed an ORR of 10% vs. 7% of the control arm and a median PFS of 3.8 vs. 2.3 months in the control arm.^62^ Volitinib (savolitinib) selectively inhibits c-Met activity in an ATP-dependent manner. The tolerability and safety of volitinib as monotherapy or in combination with gefitinib in NSCLC patients with mutant or wild-type EGFR have been studied in several phase I trials (NCT01773018, NCT02374645).^63^ It was also assessed in combination with osimertinib for patients with resistant NSCLC harboring the T790M mutation in a phase II clinical study (NCT02143466). The preliminary results showed that the combination of volitinib plus osimertinib had reliable safety and effectiveness. SAR125844 derived from triazolopyridazine is an effective specific c-Met inhibitor with an IC_50_ value of 4 nM.^64^ A phase I trial (NCT01657214) of SAR125844 displayed encouraging anti-NSCLC activity in patients with *MET* amplification, and the drug was well tolerated.^65^ More than a dozen c-Met inhibitors are currently under clinical assessment in China for the treatment of various malignancies either alone or in combination, such as bozitinib, ningetinib, glumetinib, and kanitinib. Some of them are undergoing phase II/III clinical trials and have great potential for approval in the near future.^66^

In addition to selective and non-selective small-molecule c-Met inhibitors, monoclonal antibodies against HGF ligand and c-Met are also effective strategies targeting the HGF/c-Met axis. One of the major challenges for the clinical use of these c-Met inhibitors is to distinguish the applicable population that is most likely to derive benefits from HGF/c-Met targeted therapy. It has been reported that c-Met expression detected by immunohistochemistry cannot accurately define the potential benefit patients.^67^ Biomarkers or biomarker combinations, such as *MET* mutations, *MET* gene amplification, and HGF expression, should be evaluated in cohorts receiving anti-c-Met targeted therapy to identify potential predictors of efficacy for specific c-Met inhibitors.^67,68^ For example, the *MET* exon 14 skipping mutation is a biomarker predicting the response to capmatinib and tepotinib. Drug resistance is another issue that needs to be considered in the development and clinical use of c-Met inhibitors. Multiple mechanisms have been identified to contribute to resistance to HGF/c-Met targeted therapy, including amplification of *MET* or *KRAS*, *MET* secondary mutations, induction of HGF secretion, and increased bypass activation.^67,69,70^ The clinical response to currently approved c-Met inhibitors is largely restricted to patients with *MET* amplification or exon 14 deletion. NSCLC patients who are resistant to EGFR inhibitors mediated by c-Met overexpression can also respond to c-Met inhibitors. However, inhibition of either c-Met or its ligand alone has not been proven to be potent in unselected cancer patients. Rationally designed combination strategies, such as combining c-Met inhibitors with HGF neutralizing antibodies, and cooperatively targeting upstream, downstream or parallel signaling, can not only improve the clinical benefits but also overcome drug resistance to c-Met inhibitors. Such strategies may also benefit a wide range of patients who lack *MET* gene abnormalities.

### EGFR inhibitors

The epidermal growth factor receptor (EGFR) is a transmembrane protein implicated in a wide range of biological processes. Members of this family also include ERBB2/HER2, ERBB3/HER3, and ERBB4/HER4, which are structurally similar and consist of an extramembrane ligand-binding region, a single-stranded transmembrane region, and a highly conserved intra EGFR membrane tyrosine kinase region.^71,72^ When the EGFR extracellular domain binds to its ligand, such as EGF and TGF-α, EGFR dimerizes and autophosphorylates, thereby activating downstream intracellular signaling cascades, which are closely related to cell proliferation, survival, and apoptosis.^72^ Abnormal activation of EGFR mutations is an important contributor to the tumorigenesis of multiple cancer types, especially lung cancer, breast cancer, and pancreatic cancer.^73–75^

As shown in Table 1, several EGFR TKIs are clinically available. The first generation of EGFR TKIs, such as gefitinib, erlotinib, and icotinib, are reversible inhibitors with a quinazoline structure. These drugs are highly effective in NSCLC patients harboring EGFR-activating mutations (exon 19 deletion and exon 21 L858R).^74,76,77^ They demonstrated a significant PFS benefit over platinum doublet chemotherapy in the clinic. In addition, erlotinib has also been used in combination with gemcitabine for the clinical treatment of pancreatic cancer.^78^ The EGFR L858R/T790M dual mutation is the major cause of treatment failure (>50%) after taking the first generation of EGFR inhibitors.^79^ The second-generation irreversible EGFR-TKIs afatinib and dacomitinib are designed to conquer the T790M mutation.^80,81^ They can covalently bind to the ATP-binding pocket of EGFR and show stronger pharmacological activity than gefitinib. However, they also strongly inhibit wild-type EGFR and cause severe rash and diarrhea, thereby limiting their clinical doses. Therefore, these agents are only used for NSCLC patients harboring EGFR-sensitive mutations but could not benefit sufferers harboring the T790M mutant.^79,80,82^ Novel pyrimidine-based third-generation EGFR TKIs have inhibitory effects on EGFR-activating mutations and the T790M mutation specifically but show weak inhibitory activity on wild-type EGFR. Osimertinib is the first approved third-generation EGFR inhibitor and can achieve a PFS of over 10 months in patients harboring the EGFR T790M mutation.^83^ Almonertinib, developed by Hansoh Pharma, is an analog of osimertinib. This drug also showed significant anti-cancer effects in resistant patients with NSCLC in clinical trials, and has been approved for NSCLC therapy by the NMPA recently.^84^ The success of osimertinib and almonertinib in overcoming acquired resistance is mainly attributed to their high potency and selectivity against the EGFR T790M mutation.

Lapatinib and neratinib, which are clinically available for patients with breast cancer, are dual-target inhibitors that inhibit the activities of both EGFR and HER2 (Table 1). Among them, lapatinib is a reversible TKI and is mainly used in combination with capecitabine for the treatment of advanced or metastatic breast cancers that show HER2 overexpression and have previously received treatment by anthracycline, paclitaxel, or Herceptin.^85^ Neratinib is an irreversible inhibitor mainly used in breast cancer patients who have completed standard Herceptin-assisted treatment and are currently without but at high risk of progression.^86^ Besides, tucatinib (irbinitinib) is a potent and selective HER2 inhibitor with an IC_50_ of 8 nM. This is a newly approved HER2 inhibitor, and is also used for the treatment of patients with advanced unresectable or metastatic HER2-positive breast cancer.^87^

In addition, many other EGFR inhibitors are undergoing clinical trials. Typically, olmutinib is an irreversible anilino-thienopyrimidine inhibitor of EGFR that shows high inhibitory activity against the L858R/T790M dual mutation or exon 19 deletion.^88^ Phase I and phase II trials (NCT01588145, NCT02444819, and NCT02485652) have been conducted to evaluate the efficacy and safety of olmutinib alone or in combination with drugs such as afatinib, bevacizumab, or pembrolizumab on NSCLC patients.^89^ So far this drug is only clinically available in South Korea, and has not been approved in other countries due to the potential serious side effects, such as Stevens-Johnson syndrome.^88,90^ Avitinib is an irreversible pyrrolopyrimidine derivative that is evaluated clinically for the treatment of T790M mutant NSCLC (NCT03574402).^91^ Its inhibitory activity was 300 times higher on the T790M mutant than on wild-type EGFR. Pelitinib is an irreversible fluroanilino-quinoline EGFR inhibitor. This agent has been assessed in phase II clinical trials (NCT00072748, NCT00072748) for patients with NSCLC or colorectal carcinoma.^92^ Moreover, the third-generation EGFR inhibitor furmonertinib (alflutinib) developed by Allist Pharmaceuticals is being evaluated for the treatment of NSCLC in several clinical trials (NCT02973763, NCT03452592, and NCT03787992).

Acquired resistance to EGFR TKIs develops after 9–14 months of treatment. The main causes of drug resistance include EGFR secondary mutation, activation of alternative pathways, and phenotypic transformation, especially the former. The acquired EGFR C797S mutation mediates resistance to third-generation EGFR inhibitors in ∼40% of osimertinib-treated NSCLC patients.^93^ Both *cis* and *trans* mutations of C797S and T790M were observed in resistant cases. If EGFR C797S and T790M mutations occur in trans, the combination of first- and third-generation EGFR TKIs has been reported to be an effective treatment strategy.^94^ If they occur in cis, the patients are resistant to all approved EGFR TKIs; this is also a focus for research on fourth-generation EGFR TKIs.^94^ Jia et al. reported an allosteric inhibitor EAI045 that targets both C797 and T790M mutations but spares wild-type EGFR. A combination of EAI045 and cetuximab is effective in mouse models of lung cancer driven by EGFR (L858R/T790M) and by EGFR (L858R/T790M/C797S) mutants.^95^ Moreover, Shen et al. designed and synthesized a series of 5-methylpyrimidopyridone derivatives as EGFR (L858R/T790M/C797S) inhibitors.^96,97^ The representative compound 8r-B inhibited EGFR (L858R/T790M/C797S) mutant with an IC_50_ of 27.5 nM.^97^ Further PK-oriented optimization of 8r-B is ongoing.

### FLT3 inhibitors

Fms-like tyrosine kinase 3 (FLT3), which is widely expressed in hematopoietic stem and progenitor cells, is a transmembrane protein encoded by the proto-oncogene *FLT3*. It belongs to the type III RTK family, which also includes PDGFR, FMS, and KIT. All of them consist of an extracellular ligand-binding domain, a single transmembrane hydrophobic alpha helix region, and an intracellular kinase domain. FLT3 is activated by binding to the ligands, which results in its dimerization and conformational changes. Subsequent autophosphorylation of FLT3 triggers signal transduction, activating intracellular signaling cascades such as PI3K/AKT/mTOR, RAS/RAF/MAPK, and JAK/STAT,^98,99^ which are closely related to cell proliferation, differentiation, survival, and apoptosis. FLT3 is widely overexpressed in patients with acute myeloid leukemia (AML), and its mutations lead to the constitutive activation of downstream signals.^100,101^ Internal tandem duplication (ITD) mutations in FLT3 (FLT3-ITD) are detected in ~25% of AML patients, and point mutations in the tyrosine kinase domain (TKD) are observed in 7–10% of patients.^102^ These mutations have been identified to be involved in the occurrence of leukemia. Due to the established pathogenetic and prognostic roles of FLT3-ITD and FLT3-TKD in AML, several FLT3 inhibitors have been developed for AML therapy.

The first-generation FLT3 inhibitors, including sorafenib, sunitinib, midostaurin, tandutinib, and lestaurtinib, are multikinase inhibitors.^103–105^ They are not specific for FLT3 and have inhibitory activity against various other RTKs, such as PDGFR, KIT, VEGFR, RAF, or JAK2. The clinical efficacy of most of these inhibitors as monotherapy for AML was unimpressive, and their off-target inhibition also increased adverse events.^106^ Therefore, clinical studies on first-generation FLT3 inhibitors for AML monotherapy were discontinued except for midostaurin. A randomized phase III trial (RATIFY study, NCT00651261) showed that the addition of midostaurin to cytarabine chemotherapy significantly improved overall survival (OS) for FLT3-mutated AML patients.^104^ Based on the beneficial results of the RATIFY study, midostaurin was approved by the US FDA for combination therapy with standard chemotherapy in 2017 (Table 1). Distinguishingly, pexidartinib (Turalio) is also an orally bioavailable multitarget inhibitor, with IC_50_ values of 9, 12, and 17 nM against FLT3-ITD, c-Kit, and colony-stimulating factor 1 receptor (CSF1R), respectively. However, it is not used clinically for AML therapy but approved for the treatment of adult patients with tenosynovial giant cell tumors (TGCTs). This indication is based on its inhibitory effect on CSF1R, which is frequently overexpressed in TGCTs.^107^ Second-generation FLT3 inhibitors developed by rational drug design are more potent and specific and have less toxicity related to off-target effects. Gilteritinib is the first approved second-generation FLT3 inhibitor and is also the first effective FLT3 inhibitor for AML monotherapy (Table 1).^108^ A randomized open-label phase III trial (ADMIRAL study, NCT02421939) showed that the median OS was significantly longer in the gilteritinib monotherapy group (9.3 months) than in conventional chemotherapy-treated patients (5.6 months) (*p* < 0.001). It was approved for the treatment of relapsed or refractory AML patients with FLT3 mutations in 2018. Quizartinib was screened by the KinomeScan technique to improve the affinity and specificity to FLT3 kinase, and it showed strong activity and selectivity against FLT3-ITD but not TKD.^109^ The clinical efficacy of quizartinib was also superior to conventional chemotherapy (NCT00989261); therefore, it was approved for relapsed or refractory AML patients with FLT3-ITD mutations in 2019.^110^

Currently, many promising FLT3 inhibitors are still under clinical evaluation. Crenolanib, originally developed as an inhibitor of PDGFR, is also a second-generation FLT3 inhibitor with inhibitory activity against both FLT3-ITD and FLT3 D835 mutations.^111^ Crenolanib development focused on assessing the combination effects of this drug with conventional chemotherapy in terms of first-line and relapse treatment. Several clinical trials are underway to evaluate the clinical efficacy of crenolanib, including a randomized phase III trial evaluating the potency of crenolanib in combination with induction chemotherapy for relapsed or refractory FLT3-mutated AML patients (NCT02298166) and a multicentre phase III trial comparing the effects of crenolanib with midostaurin during induction chemotherapy and consolidation therapy for newly diagnosed FLT3-mutated AML patients (NCT03258931). SKLB-1028 is a multitarget inhibitor with FLT3 inhibitory activity.^112^ A phase I trial (NCT02859948) was conducted to evaluate the safety, tolerability and pharmacokinetic characteristics of SKLB-1028 in FLT3 mutant AML subjects.^113^ Moreover, it has been reported that SKLB-1028 also has inhibitory activity on FLT3 secondary mutations such as FLT3-D835Y and FLT3-F691L; therefore, it is considered a potential therapeutic drug for resistant AML patients harboring the corresponding mutations.^114^ The Bcr-Abl1 inhibitor ponatinib is currently approved for chronic myeloid leukemia (CML) and acute lymphoblastic leukemia (ALL) therapy, and it is also a FLT3 inhibitor with potent inhibition of FLT3-ITD.^115^ Phase I/II studies are ongoing to evaluate the efficacy and safety of ponatinib in combination with cytarabine for AML patients with the FLT3-ITD mutation (NCT02428543) and its monotherapy or combination with azacitidine for untreated AML patients who are unfit for conventional chemotherapy (NCT02829840). In addition, the anti-AML activity and safety of several other multikinase inhibitors with FLT3 inhibitory activity, such as AT-9283, ENMD-981693, 4SC-203, cabozantinib, and CR-4, are also being evaluated in the clinic (NCT01054937, NCT01961765).^116^

Primary and secondary resistance is a challenging issue for TKI treatment, including FLT3 inhibitors, which results in the limited and transient efficacy of FLT3 inhibitors. Primary resistance to FLT3 inhibitors involves insensitive FLT3 mutations, expression of FGF2 or CYP3A4 in the bone marrow microenvironment, upregulation of anti-apoptotic proteins MCL-1, BCL-XL, or BCL-2, and other activated signals. Acquired resistance includes TKD secondary mutations at the activating loop residues (e.g., D835, D839, I836, and Y842) or the gatekeeper site F691,^117,118^ autocrine FLT3 signaling, and activation of alternative pathways. The understanding of the molecular mechanisms associated with resistance to FLT3 inhibitors lays a foundation for establishing strategies to overcome and reduce resistance. Many combination strategies have been evaluated to improve the treatment outcome, such as the combination with epigenetic therapy (e.g., HDAC inhibitors), proteasome inhibitors, and inhibitors targeting independent signaling pathways or downstream pathways of FLT3-ITD (e.g., STAT5 inhibitor, CDK4/6 inhibitors, PI3K/mTOR inhibitors).^119–123^ Another strategy is to develop irreversible FLT3 inhibitors. FF-10101 is a covalent-binding FLT3 inhibitor and can maintain the ability to bind FLT3 in either an active or inactive conformation.^124^ The irreversible binding of FF-10101 provides potent inhibitory effects on multiple secondary mutations, such as F691L gatekeeper mutation. A phase I/IIa study (NCT03194685) was conducted to assess its safety, tolerability, pharmacokinetics, and efficacy in subjects with relapsed or refractory AML. Meanwhile, a variety of novel FLT3 inhibitors, including 7 h,^125^ PLX3379,^126^ and MZH29,^127^ are in the preclinical research and development stage. They are potential next-generation FLT3 inhibitors to conquer secondary mutation-mediated resistance.

### VEGFR/FGFR/PDGFR inhibitors

Angiogenesis is a complex process through which new blood vessels form from pre-existing vessels.^128^ In physiological circumstances, angiogenesis is strictly regulated by various endogenous pro-angiogenic and anti-angiogenic factors.^129^ Aberrant angiogenesis exists in a wide range of diseases including arthritis, retinopathies, atherosclerosis, endometriosis, and cancer.^130–132^ In 1971, Judah Folkman raised the hypothesis that solid tumors cause new blood vessel growth (angiogenesis) in the tumor microenvironment by secreting pro-angiogenic factors, initiating the research between angiogenesis and cancer.^133^ Angiogenesis is critical for the development and subsequent growth of human solid tumors; otherwise, tumor size will not exceed 1–2 mm.^134^ Tumors require new blood capillaries to provide nutrient and oxygen, remove metabolic waste, and facilitate the formation of metastases.^135,136^ As an increasing number of tumor angiogenesis-related genes, transcription factors, signaling pathways, and their mechanisms of action have been revealed, anti-angiogenesis has become an attractive strategy for cancer therapy.^137,138^ Well-known pro-angiogenic factors mediating the angiogenic switch include vascular endothelial growth factor (VEGF),^139^ basic fibroblast growth factor (bFGF),^140^ platelet-derived growth factor (PDGF),^141^ transforming growth factor (TGF),^142^ insulin-like growth factor, epidermal growth factor (EGF),^143^ and angiopoietin.^144^ In the past few years, efforts to develop anti-angiogenic treatments have mainly focused on inhibiting the activities of their receptors such as VEGF receptors (VEGFR-1-3), FGF receptors (FGFR1–4), PDGF receptors (PDGFRα and PDGFRβ), and TGF-β receptors (TGF-βRI, TGF-βRII, and TGF-βRIII).^131,145,146^

Currently, more than 10 anti-angiogenic TKIs have been approved by the FDA and NMPA of China for the treatment of multiple solid malignancies, and most of them are multikinase inhibitors (Table 1). Sorafenib can inhibit a number of receptor tyrosine kinases (RTKs) including VEGFR-1/2/3, c-Kit, FLT3, RET, PDGFRβ, and RAF, and is the first approved anti-angiogenic inhibitor.^147^ It was initially approved for the treatment of advanced RCC in 2005. Subsequently, the FDA-approved sorafenib for the treatment of advanced hepatocellular carcinoma (HCC) in 2007 based on encouraging results from the SHARP trial, and for differentiated thyroid carcinoma (DTC) in 2013 based on beneficial results from the DECISION trial.^148,149^ Sorafenib is also the first small-molecule targeted drug to be approved for these three cancer indications. The multikinase inhibitor lenvatinib approved in 2015 has the same clinical indications as sorafenib, and they are currently the only two targeted agents used clinically for the first-line treatment of HCC.^148,150–152^ Other approved anti-angiogenic inhibitors for the first- or second-line treatment of RCC or DTC include sunitinib^153^ (2006), pazopanib^154^ (2009), axitinib^155^ (2012), cabozantinib^156^ (2016), and tivozanib^157^ (2017). Among them, sunitinib, an indol-2-one multikinase inhibitor targeting VEGFR-1/2/3, PDGFRα/β, c-Kit, CSF1R, RET, and FLT3, is the second approved anti-angiogenic TKI, and was simultaneously approved for two distinct indications including RCC and imatinib-resistant gastrointestinal stromal tumor (GIST).^158,159^ The anilinoquinazoline derivative vandetanib inhibits the activities of EGFR, VEGFR-2/3, RET, BRK, TIE2, and EPH. It is the first drug to be approved for the treatment of adult patients with metastatic MTC by the FDA.^160^ However, this indication is most likely attributed to its inhibitory effect on RET, a tyrosine kinase hyperactivated by mutations in MTC.^161^ Another anti-angiogenic inhibitor used for the clinical treatment of MTC is cabozantinib, which also has high RET inhibitory activity.^162^ Relatedly, the FDA-approved two highly specific RET inhibitors (selpercatinib and pralsetinib) in 2020. Both of them show a wide range of therapeutic effects on RET-driven (*RET* mutation or *RET* fusion-positive) malignancies in clinical trials and have been approved for the treatment of advanced or metastatic *RET*-mutant MTC, *RET* fusion-positive NSCLC, and radioactive iodine-refractory thyroid cancer (Table 1).^163,164^ Regorafenib developed by Bayer is a fluoro-derivative of sorafenib with activity against multiple kinases including VEGFR-1/2/3, PDGFRα/β, FGFR1/2, BRAF, c-Kit, and RET.^165^ It has shown clinical effectiveness for patients with metastatic colorectal cancer (mCRC), who progress after prior standard treatment (NCT01103323), and received FDA approval in 2012.^166^ Afterward, the FDA expanded its indication to advanced GIST in 2013 based on the results of GRID clinical trial (NCT01271712). In this phase III study, although no difference was observed in the OS between regorafenib and placebo groups (hazard ratio = 0.77, *p* = 0.199), the PFS was significantly improved to 4.8 months in the treatment group, and the placebo arm was just 0.9 months.^167^ The indolinone derivative nintedanib targets VEGFR-1/2/3, FGFR1/2, and PDGFRα/β.^168^ It was initially used clinically for the treatment of idiopathic pulmonary fibrosis^169^ and the combination therapy of nintedanib and docetaxel was approved as a second-line treatment for patients with NSCLC in the same year by the European Medicines Agency (EMA) but not the FDA.^170^ In the past decade, Chinese researchers have also made great progress in developing anti-angiogenic drugs, and several have been approved by the NMPA of China. Apatinib developed by Hengrui Medicine inhibits the activities of VEGFR-2, c-Src, and c-Kit simultaneously and was approved for the treatment of advanced gastric cancer in October 2014.^171^ The multitarget inhibitor anlotinib is developed by Chiatai Tianqing.^172^ This anti-angiogenic agent has been used for the treatment of several malignant tumors including NSCLC, soft tissue sarcoma (STS), and small cell lung cancer (SCLC).^173^ These two drugs have been identified as orphan drugs by the FDA, but have not been launched in the United States. Fruquintinib developed by Hutchison Whampoa Limited is a potent small-molecule inhibitor of VEGFR-1/2/3.^174^ The FRESCO trial, a randomized double-blind phase III study (NCT02314819), laid the foundation for the approval of fruquintinib in patients with mCRC in 2018.^175^ Its effectiveness and safety are being explored in a phase I trial conducted in a non-Chinese population in the United States.^176^

In addition to anti-angiogenic effects, several selective FGFR or PDGFR inhibitors have also been approved recently, mainly functioning as therapeutic agents for FGFR or PDGFR-driven malignancies (Table 1). Erdafitinib is an orally potent pan-FGFR inhibitor with IC_50_ values of 1.2, 2.5, 3.0, and 5.7 nM against FGFR1, 2, 3, and 4, respectively.^177^ As the first approved FGFR-selective inhibitor, it has been used for the second-line treatment of locally advanced or metastatic urothelial carcinoma. Meanwhile, it is undergoing clinical development as a treatment for other malignancies including NSCLC, gastric cancer, prostate cancer, cholangiocarcinoma, esophageal cancer, and lymphoma.^178^ Pemigatinib, also known as INCB054828, is an orally potent FGFR-selective inhibitor with IC_50_ values in the nanomolar range against FGFR1-3. Based on the results of FIGHT-202 (NCT02924376), a phase II, open-label, single-arm, multicenter study to evaluate the efficacy and safety of pemigatinib in cholangiocarcinoma subjects, pemigatinib received accelerated approval in April 2020 for the treatment of patients with previously treated, advanced/metastatic, or surgically unresectable cholangiocarcinoma harboring FGFR2 fusions or other rearrangements.^179^ This is also the first approved targeted treatment for cholangiocarcinoma.^180^ Pemigatinib is being evaluated for clinical use in several other FGFR-driven cancers and received orphan designation in August 2019 by the FDA for the treatment of myeloid/lymphoid neoplasms with eosinophilia and rearrangement of FGFR1 or PDGFRα/β.^179^ Avapritinib, developed by Blueprint Medicines, is an effective and selective inhibitor of PDGFRα and KIT activation loop mutants, which has recently been approved in the United States for adults with unresectable or metastatic GIST harboring PDGFRα exon 18 mutations.^181^ GIST can be classified according to different molecular subtypes, and KIT or PDGFRα mutated GISTs are important subgroups that commonly arise in the stomach. Five TKIs of PDGFRs or KIT (imatinib, sunitinib, regorafenib, avapritinib, and ripretinib) are currently used clinically for GIST therapy.^182^ As target-specific inhibitors, avapritinib and ripretinib have shown extensive inhibitory effects on KIT or PDGFRα mutant GIST, and are potent for patients harboring primary or secondary resistant mutations, including the PDGFRα D842V mutant.^183–185^

Efforts are being made to expand many of the above-mentioned approved VEGFR, FGFR, or PDGFR inhibitors to other cancer indications. Meanwhile, a large number of novel inhibitors are also being developed, many of which have entered clinical trials. Several representative drug candidates with the potential to receive approval in the near future are presented here. Cediranib developed by AstraZeneca is a potent multikinase inhibitor targeting VEGFR-1/2/3, PDGFRα/β, and c-Kit.^186^ However, AstraZeneca discontinued the development of cediranib for the treatment of mCRC, NSCLC, and recurrent glioblastoma due to the mediocre results from phase III clinical trials (NCT00399035, NCT00795340, NCT00777153) in these indications. At present, it shows new hope in the combination therapy of ovarian cancer, and two phase III trials (NCT03278717 and NCT02446600) are ongoing to compare the efficacy of cediranib plus olaparib with olaparib alone or in patients with ovarian cancer.^187^ Dovitinib, also named CHIR-258, is a multitargeted anti-angiogenic inhibitor with IC_50_s of 10/13/8, 2, 1, 8/9, and 27/210 nM for VEGFR-1/2/3, c-Kit, FLT3, FGFR1/3, and PDGFRα/β, respectively.^188^ In an open-label, randomized, phase III study to compare the safety and efficacy of dovitinib vs. sorafenib in patients with metastatic RCC (NCT01223027), there was no difference in PFS between these two drugs in third-line treatment.^189^ It received orphan drug designation for the treatment of adenoid cystic carcinoma in 2019. Motesanib is an orally administered multikinase inhibitor of VEGFR-1/2/3, PDGFRα/β, c-Kit, and RET, and was considered a potent anti-NSCLC drug in Asian patients based on the MONET1 trial.^190^ However, the results of a later phase III trial (NCT02629848) evaluating the efficacy of motesanib plus paclitaxel and carboplatin were disappointing. Motesanib plus paclitaxel/carboplatin did not significantly improve PFS vs. placebo plus paclitaxel/carboplatin (median PFS: 6.1 verse 5.6 months) in East Asian patients with stage IV/recurrent non-squamous NSCLC.^191^ Nevertheless, it showed marked anti-cancer effects in patients with advanced thyroid cancer in two phase II studies (NCT00121628, NCT02084732).^192,193^ Recently, a phase II study (NCT00121628) assessing the efficacy of motesanib in low-grade neuroendocrine tumors (NETs) also achieved satisfactory treatment results with a 4-month PFS of 78.5%, and the median PFS of all patients was 8.7 months.^194^ Sulfatinib is a potent inhibitor against VEGFR-1/2/3, FGFR1, and CSF1R with IC_50_ values in the range of 1–24 nM.^195^ An encouraging antitumor activity and acceptable safety profile were observed in a phase I trial (NCT02133157), particularly in NETs.^196^ It is currently being evaluated in advanced NETs in two phase III studies (NCT02589821 and NCT02588170).^197^ Crenolanib is an orally bioavailable TKI of PDGFRα/β and FLT3.^198^ A multicenter, randomized, double-blinded, phase III trial (NCT02847429) was conducted to assess the efficacy of oral crenolanib vs. oral placebo in combination with best supportive care in subjects with advanced or metastatic GIST with PDGFRα D842V mutation.^199^ In addition, due to its high inhibitory activity on both FLT3-ITD and FLT3-TKD mutant subtypes, which are important therapeutic targets for AML, crenolanib is also evaluated in a phase III randomized multicenter study conducted in AML subjects with FLT3 mutation (NCT03258931).^200^ Lucitanib, developed by Shanghai HaiHe Biopharma, potently and selectively inhibits VEGFR-1, VEGFR-2, VEGFR3, FGFR1, and FGFR2 with IC_50_ values of 7, 25, 10, 17.5, and 82.5 nM, respectively.^201^ A multicenter phase III study is being conducted to evaluate the efficacy of lucitanib in combination with carboplatin plus etoposide in untreated participants with extensive-stage SCLC (NCT04254471). Brivanib, in particular, is an orally active L-alanine ester prodrug that inhibits VEGFR-2 with an IC_50_ of 25 nM, and has moderate potency against VEGFR-1 and FGFR1.^202^ It is currently being evaluated in a phase III clinical trial on subjects with advanced HCC, who have failed or are intolerant to sorafenib (NCT00858871).^203,204^ Meanwhile, multiple noncovalent FGFR-selective inhibitors (pan-FGFR inhibitors) have also advanced to phase III trials, including the well-known AZD4547^205^ and infigratinib,^206^ both of which are typical type-I inhibitors. The former is being assessed for the treatment of patients with stage IV SCLC in phase II/III trial (NCT02965378), and the latter was granted orphan drug designation by the FDA for the treatment of cholangiocarcinoma in 2019.^207^ In addition, targeting TGF-β signaling is also a potential strategy for anti-angiogenic therapy.^208^ However, no TGF-βR inhibitor has been approved for clinical use, and several drug candidates are undergoing evaluation in clinical trials including galunisertib, vactosertib, LY-3200882, PF-06952229, YL-13027, and GFH018.^209–211^ Galunisertib, a selective TGF-βR type-I (TGF-βRI) kinase inhibitor with an IC_50_ of 56 nM, is the most advanced drug candidate among them.^209,212^ It has been evaluated clinically in several solid tumors including HCC, glioma, and glioblastoma multiforme, and achieved remarkable effect in the treatment of HCC. The median OS of galunisertib monotherapy for HCC can reach 16.8 months.^213,214^

The concept of “starving tumors to death” by inhibiting tumor angiogenesis has promoted the development of anti-angiogenic therapy.^215^ However, many anti-angiogenesis drugs produced only modest survival benefits for cancer patients in clinical trials.^216,217^ One of the reasons can be explained by vascular normalization; this theory emphasizes that anti-angiogenesis agents mainly selectively block the formation of immature blood vessels rather than the mature and functional vasculatures. Therefore, anti-angiogenesis treatment alone is generally ineffective unless combined with chemotherapy.^218,219^ On the other hand, tumor angiogenesis is regulated by multiple signaling pathways, and many interconnected pathways can compensate for the effect of single inhibition of one of these signals, such as the VEGFR pathway. This is why the approved small-molecule anti-angiogenesis TKIs are mostly multitarget inhibitors and only several selective inhibitors are used clinically for cancer patients with specific mutations. This also indicates the importance of combination therapy in the clinical use of anti-angiogenesis agents.^220,221^ Accumulating evidences have demonstrated that anti-angiogenic therapy can not only inhibit the formation of neo-vascular, but also regulate the immune microenvironment, which provides a theoretic basis for the combination of anti-angiogenesis agents with immunotherapy.^221^ Hundreds of clinical studies have been designed to evaluate such combination strategies. Encouragingly, the combination of axitinib with PD-1 antibody pembrolizumab has been approved for the treatment of patients with advanced RCC in 2019.^222^ Another challenge of anti-angiogenic therapy is the lack of more personalized use of existing anti-angiogenesis agents. This is also a significant feature that distinguishes most anti-angiogenesis TKIs from other molecular targeted therapies; the former is given to unselected patients within approved indications, while the latter are used in proper patients selected by robust biomarkers, which markedly improve their clinical benefits. Most efforts have been made to identify molecular biomarkers for anti-angiogenesis agents, such as expression of VEGF and FGF in blood and tumors,^223^ tumor perfusion status,^224^ and other angiogenic factors, but none of them have yet been validated for routine clinical use. Recently, several studies have suggested that anti-angiogenesis-related side effects, such as hypothyroidism, high blood pressure, or hand-foot syndrome, may be associated with the antitumor efficacy of angiogenesis inhibitors.^223,224^ As the lack of reliable predictive biomarkers in the clinic, these side effects may contribute to clinical decision, but further clinical verification is still needed.

### TRK inhibitors

The tropomyosin receptor kinase (TRK) family is composed of three members, TRKA, TRKB, and TRKC, which are encoded by the neurotrophic tyrosine receptor kinase (NTRK) genes *NTRK1*, *NTRK2*, and *NTRK3*, respectively.^225^ To activate TRK receptors, neurotrophins (TRK ligands) bind to the extracellular domain of the receptors, stimulating homodimerization and autophosphorylation of TRK proteins, thereby activating downstream signaling pathways, such as RAS/MAPK/ERK, PI3K/AKT, and PLCγ. *NTRK* gene rearrangements containing a kinase domain of one of the three TRKs and a dimerization domain of another gene generate fusion proteins and result in aberrant activation of TRKs, which have been identified as oncogenic drivers of various cancers.^226–228^ Therefore, TRKs are emerging as important targets for cancer therapy. The rearrangements of *NTRK* genes occur in only 1% of all malignancies; they have been widely detected at low frequencies in some common cancers, such as lung cancer, thyroid carcinoma, glioblastoma, and colorectal cancer. However, in several rare pediatric and adult cancer types, including infantile fibrosarcoma, secretory breast carcinoma, and salivary gland secretory carcinoma, *NTRK* gene rearrangements are common.

The discovery of TRK inhibitors renewed interest in *NTRK* gene rearrangements as oncogenes. Currently, two first-generation TRK inhibitors are available for clinical cancer treatment (Table 1). Larotrectinib is the first approved selective oral pan-TRK inhibitor with high potency against TRKA, TRKB, and TRKC.^229^ Entrectinib (RXDX-101/NMS-E628) is a potent multikinase inhibitor targeting TRKA/B/C, ROS1, and ALK.^230^ Both agents received the FDA breakthrough therapy identification; this breakthrough designation highlights the efficacy of TRK inhibitors in various cancers that have the same mutation, regardless of cancer type and patient age. Based on the tumor-agnostic efficacy of the “basket trail” conducted in diverse *NTRK* fusion-positive cancers, larotrectinib and entrectinib granted FDA approval for the treatment of adult and pediatric patients with TRK fusion solid tumors. In clinical use, *NTRK* gene fusions should be diagnosed to select patients for targeted TRK therapy. Remarkably, both larotrectinib and entrectinib displayed activity against CNS tumors with *NTRK* fusions, indicating the ability for BBB penetration.^231,232^ When referring to the adverse events of first-generation TRK inhibitors, it should be noted that both agents have favorable overall safety profiles compared to other small-molecule TKIs. These drugs are generally well-tolerated in patients, with low incidences of dose reductions, discontinuations, and grade 3–4 adverse events.^225^

Recently, there have been several small-molecule TRK inhibitors in different stages of clinical research, some of which target multiple kinases, such as cabozantinib (targeting c-Met, RET, VEGFR-2, ROS1, ALK, and TRK),^233^ merestinib (targeting c-Met, TEK, ROS1, and TRK),^234^ belizatinib (targeting ALK and TRK),^235^ sitravatinib (targeting c-Met, RET, AXL, and TRK),^236^ altiratinib (targeting c-Met, TIE2, VEGFR-2, FLT3, and TRK),^237^ and DS-6051b (targeting ROS and TRK).^238^ These inhibitors displayed varying degrees of inhibitory activity against TRK. Some of them have been approved for indications other than TRK fusion tumors; for example, cabozantinib was approved as an anti-angiogenic inhibitor for the treatment of patients with advanced RCC in 2016, and data are limited on its efficacy against *NTRK* fusions. However, with the increasing interest in TRK as a cancer therapy target, an increasing number of clinical trials have been performed to evaluate the effects of these inhibitors in patients with TRK fusion-positive tumors. In addition, a phase I study was carried out to assess the safety, PKs, and PDs of the selective TRK inhibitor PLX7486 as a single agent in patients with any histological solid tumors with activating *NTRK* point or *NTRK* fusion mutations (NCT01804530).^239^ However, the results were not disclosed.

Acquired resistance to TKI treatment can be mediated by on-target mutations or off-target (bypass activation) mechanisms. Until now, on-target mutations in the kinase domain of *NTRK* fusion have been the only resistance mechanism of first-generation TRK inhibitors, which can result in amino acid substitutions of the solvent front, activation loop xDFG motif, and gatekeeper residues in the kinase domain of TRK fusion proteins, interfering with TRK inhibitor binding.^225^ The first resistance case to TRK inhibition was discovered in a colorectal cancer patient treated with entrectinib, and two acquired resistance mutations, TRKA G595R and TRKA G667C, were detected in the plasma cfDNA of this patient.^240^ Several other resistant mutations were subsequently identified in patients resistant to larotrectinib and entrectinib, including the acquired TRKC G623R substitution, A608D mutation and gatekeeper F589L substitution in TRKA, and the substitutions involving the xDFG site of TRKA (G667S) and TRKC (G696A).^229,241^ Fortunately, next-generation TRK inhibitors are currently in development to overcome acquired resistance to larotrectinib and entrectinib. In particular, selitrectinib (LOXO-195), repotrectinib (TPX-0005), and ONO-5390556 have demonstrated nanomolar inhibitory activity against the TRK mutants mentioned above.^242^ Among them, the safety and efficacy of selitrectinib and repotrectinib are currently under assessment in phase I/II trials (NCT04275960, NCT04094610).

## Non-receptor tyrosine kinase inhibitors

### Bcr-Abl1 inhibitors

c-Abl is encoded by the abelson murine leukemia 1 (*ABL1*) gene on chromosome 9 and belongs to the Abl family of non-receptor tyrosine kinase; it has been implicated in a range of cellular processes including the regulation of cell differentiation, cell cycle, and survival. Philadelphia (Ph) chromosome translocation results in the molecular juxtaposition of *ABL1* and the breakpoint cluster region (*BCR*) of chromosome 22, forming an aberrant *BCR-ABL* fusion gene on chromosome 22.^243^ This gene encodes a 210 kDa oncoprotein (p210 Bcr-Abl1) that is capable of autophosphorylation and constitutively activates the downstream pathway, thereby driving the uncontrolled proliferation of leukemia cells in almost all cases of CML and ~20% of patients with ALL.^244–246^ The *BCR-ABL* fusion gene was identified as a specific biomarker for diagnosis and prediction of response to treatment, while Bcr-Abl1 fusion tyrosine kinase is considered to be a susceptible target for certain leukemias.

As indicated in Table 2, imatinib is the first approved Bcr-Abl1 inhibitor as well as the first approved small-molecule TKI, which launches a new era of tumor-targeted therapy. This agent has shown striking activity in patients with chronic phase CML (CML-CP) and Ph^+^ ALL.^247^ A 5-year follow-up study conducted in patients with CML-CP receiving interferon or imatinib treatment showed that the OS and PFS of patients taking imatinib could reach 89% and 93%, respectively.^248^ The introduction of imatinib for the treatment of CML patients with Ph chromosome translocation provides a proof-of-principle for using aberrant kinases as therapeutic targets. Currently, this drug represents the gold therapeutic standard in patients with CML in the clinical setting. Although treatment with imatinib has achieved exciting results, drug resistance caused by point mutations in the kinase domain of *BCR-ABL* has frequently emerged such as G250E, Q252H, Y253H/F, and E255K/V mutations located in the P loop region, T315I mutation in the ATP-binding region, and H395P/R mutation in the activation region.^248–251^ Point mutations decrease the affinity of imatinib to the Bcr-Abl1 kinase domain, resulting in reduced imatinib inhibitory activity.^249,252^ The increasing recognition of imatinib resistance stimulates the development of second-generation Bcr-Abl1 inhibitors including dasatinib, nilotinib, bosutinib, and radotinib, which were approved in 2006, 2007, 2012, and 2012, respectively.^253–256^ Both dasatinib and bosutinib are oral dual Src/Abl1 kinase inhibitors, and the former is ~300-fold more potent than imatinib. Nilotinib, an aniline pyrimidine derivative developed from imatinib by crystallographic analysis and structural modification, has better lipophilicity and solubility and ~30-fold higher potency. Radotinib is the structural analog of nilotinib and is used as a second-line treatment in the clinic. These inhibitors can suppress most clinically relevant *BCR-ABL* mutants, except T315I gatekeeper mutation, which occurs in up to 20% of patients with resistant CML.^256,257^ Ponatinib is a third-generation Bcr-Abl1 inhibitor with activity against T315I mutation.^258^ The binding pattern of ponatinib is similar to imatinib, except that the carbon-carbon triple bond extending from the purine of ponatinib enforces compatibility with T315I residue. It is currently approved for the treatment of patients with CML or ALL that are either resistant or unable to tolerate other Bcr-Abl1 inhibitors. In addition, due to the multitarget properties of imatinib, dasatinib, and nilotinib, they were also evaluated clinically for the treatment of some solid tumors. Among them, imatinib was approved for GIST therapy in 2003 (Table 2).

**Table 2:** Properties of approved small-molecule inhibitors of non-receptor tyrosine kinases

Chemical structure	Name	Targets	Approved indications (year)	Corporation
	Imatinib (Gleevec)	Bcr-Abl/PDGFR-β/c-Kit	CML (2001) GIST (2003) ALL (2006)	Novartis
	Dasatinib (Spraysel)	Bcr-Abl/Src/c-Kit/LCK/PDGFR-β	CML (2006) ALL (2006)	Bristol-Myers Squibb
	Nilotinib (Tasigna)	Bcr-Abl/DDR1/2	CML (2007)	Novartis
	Bosutinib (Bosulif)	Abl1/Src	CML (2012)	Pfizer
	Radotinib (Supect)	Bcr-Abl	CML (2012)	IL-Yang
	Ponatinib (Iclusig)	Bcr-Abl /PDGFR-α/VEGFR-2/FGFR-1/Src/FLT3/c-Kit	CML (2013) ALL (2013)	Incyte/Takeda
	Ibrutinib (Imbruvica)	BTK	MCL (2013) CLL (2014) WM (2015) SLL (2016) MZL (2017)	AbbVie/ Johnson & Johnson
	Acalabrutinib (Calquence)	BTK	MCL (2017)	AstraZeneca
	Zanubrutinib (Brukinsa)	BTK	MCL (2019)	BeiGene
	Ruxolitinib (Jakafi)	JAK1/2	Myelofibrosis (2011)	Incyte/Novartis
	Fedratinib (Inrebic)	JAK2	Myelofibrosis (2019)	Impact

Research on the development of novel Bcr-Abl1 inhibitors against drug-resistant mutations is ongoing. To date, up to 13 inhibitors have entered clinical trials. Typically, asciminib (ABL001) is a potent and selective allosteric Abl1 inhibitor, which binds to the myristoyl pocket of Abl1 and induces the formation of an inactive kinase conformation.^259^ Phase I clinical trials are being conducted to evaluate the efficacy and safety of this drug alone or in combination with dasatinib and prednisone in patients with CML or *BCR-ABL*-positive B-cell ALL (NCT02081378, NCT03595917). Meanwhile, it is also evaluated in phase III clinical trial to compare its efficacy with bosutinib in patients with CML-CP (NCT03106779). Rebastinib (DCC-2036) is a potent conformational control inhibitor, designed to conquer Bcr-Abl1 resistant mutations, mainly T315I.^260^ It induces the kinase to a catalytically inactive state, regardless of gatekeeper mutations. Clinically, a phase I dose-finding study of rebastinib in patients with relapsed CML has been completed (NCT00827138); however, the clinical benefit was considered insufficient to support its continued use in leukemia treatment since the advent of ponatinib. Bafetinib (INNO-406) was developed to expand the susceptibility spectrum of mutations to TKIs and increase the selectivity to Bcr-Abl1 to reduce adverse reactions.^261^ In a phase I trial (NCT00352677), bafetinib as second-line treatment can achieve complete cytogenetic response in 19% of patients with CML and Ph^+^ ALL that is resistant or intolerant to imatinib, indicating its potential clinical efficacy.

Outcomes for patients with CML have been greatly improved since the clinical application of Bcr-Abl1 TKIs. However, despite the high initial response rate of these inhibitors, drug resistance and adverse events are two main problems influencing the achievement of the best response and quality of life for patients. Sequential therapy with Bcr-Abl1 inhibitors leads to the continuous acquisition of novel mutations, especially compound mutants, which refers to the accumulation of more than one mutation in the same allele. Ponatinib is the latest generation of Bcr-Abl1 inhibitor, and patients treated with this drug have developed new resistance mutations, such as T315M single-point mutation and complex mutations T315I/E255V and E255V/Y253H.^262–264^ Overcoming these resistance mutations requires the development of next-generation inhibitors and combination therapy strategies. Meanwhile, it has also been reported that some mutations are sensitive to the second-generation inhibitors, such as T315A to nilotinib, E255K/V and Y253H to dasatinib and bosutinib, and patients harboring such mutations can be retreated with these drugs. The clinical use of ponatinib is associated with cardiovascular events, and concerns about arterial thrombosis may limit its treatment in some patients with T315I mutations. For these patients, omacetaxine mepesuccinate approved by the FDA in 2012 is a proper treatment option.^265,266^ It has shown encouraging therapeutic results in patients harboring T315I mutation and is tolerable without cardiovascular toxicity. Moreover, some patients with GIST and systemic mastocytosis can benefit from the treatment of imatinib, dasatinib, or nilotinib due to the broad-spectrum selectivity of them against c-Kit, PDGFR, or Src.^267,268^ Mutations of these kinases can also drive the selection of appropriate inhibitors. An in vitro study showed that imatinib-resistant mutations PDGFRα D842V and c-Kit D816V that commonly occur in GISTs and mastocytosis, respectively, could be strongly inhibited by dasatinib.^269^

### BTK inhibitors

The B-cell receptor (BCR) pathway has a key role in the progression of a variety of B-cell malignancies.^270^ Abnormal activation of BCR signaling has been identified in multiple heterogeneous hematologic malignancies, including B-cell non-Hodgkin’s lymphoma (NHL), chronic lymphocytic leukemia (CLL), small lymphocytic lymphoma (SLL), mantle cell lymphoma (MCL), marginal zone lymphoma (MZL), follicular lymphoma, Waldenstrom’s macroglobulinemia (WM), and DLBCL.^271^ Bruton’s agammaglobulinemia tyrosine kinase (BTK), a crucial component of the BCR pathway, belongs to the non-receptor tyrosine kinase of the TEC family, which contains four other members: tyrosine kinase expressed in hepatocellular carcinoma (TEC), interleukin-2-inducible T-cell kinase (ITK), resting lymphocyte kinase (RLK/TXK), and bone marrow expressed kinase (BMX).^272^ BTK is abundantly expressed in B-cell leukemias and lymphomas and functions as a vital regulator of cell proliferation and survival in various B-cell malignancies.^273^ Inhibiting BTK is considered an effective therapeutic strategy for some hematologic malignancies.^274^

Ibrutinib is the first-generation BTK inhibitor and has been proven to be superior to standard chemotherapy in multiple studies including older patients with significant comorbidity. It is an irreversible small-molecule inhibitor that covalently binds to Cys-481 within the ATP-binding pocket of BTK. Based on the high response rates and durable responses of its monotherapy^275^ or in combination with anti-CD20 antibody,^276^ ibrutinib has been approved by the FDA for the treatment of MCL, CLL, WM, SLL, and MZL between 2013 and 2017 (Table 2).^277–279^ The clinical efficacy of ibrutinib in the treatment of DLBCL, refractory/recurrent primary central nervous system lymphoma, and secondary central nervous system lymphoma is still undergoing evaluation to expand its indications.^280^ Despite the clinical achievement of ibrutinib, side effects including arthralgia, atrial fibrillation, pneumonitis and rash have also been reported and limit its clinical use. Most of the toxicity of ibrutinib is due to its off-target activities against four other TEC family kinases, EGFR, HER2, and Janus kinase 3 (JAK3).^281^ Particularly, in combination therapy with the CD20 antibody rituximab, off-target inhibition of ITK by ibrutinib led to an antagonistic effect on antibody-dependent cell-mediated cytotoxicity, influencing the combined effects.^282^ The off-target activity of ibrutinib triggered the development of more selective second-generation BTK inhibitors. Acalabrutinib^283^ and zanubrutinib^284^ are currently approved second-generation BTK inhibitors (Table 2). Similar to ibrutinib, they are irreversible inhibitors and form covalent bonds with the Cys-481 residue of the BTK active site.^284^ And their selectivity is significantly improved. Acalabrutinib inhibited BTK with an IC_50_ of 3 nM and had less off-target activity on EGFR, ITK, or TEC;^285^ zanubrutinib had similar inhibitory activity to ibrutinib against BTK, but its IC_50_s on TEC, ITK, EGFR, HER2, and JAK3 were 2–70 times higher than those of ibrutinib.^286^ Currently, the FDA has approved them for the treatment of adult MCL patients who have received at least one prior therapy.^287^ Both of them are still evaluated in the clinic for the treatment of some other malignancies, such as NHL,^288^ multiple myeloma (MM),^289^ and ovarian cancer.^290^

Several promising BTK irreversible inhibitors are under clinical evaluation.^291^ Tirabrutinib (ONO-4059) is a highly selective covalent inhibitor of BTK with an IC_50_ of 2.2 nM. In a phase I trial conducted in patients with B-cell malignancies (NCT02457559), tirabrutinib showed significant potency on patients in the CLL group. Ninety-six percent of CLL patients responded to tirabrutinib, and all of the evaluated CLL patients harboring del 17p or TP53 mutations without del 17p responded.^292^ Moreover, a phase II trial (NCT02968563) is underway to assess the efficacy and safety of tirabrutinib in combination with the PI3K inhibitor idelalisib and the anti-CD20 antibody obinutuzumab.^293^ Spebrutinib (CC-292/AVL-292) is also a second-generation BTK inhibitor and inhibits BTK activity with an IC_50_ of 0.5 nM.^294^ The results of phase I studies (NCT01692184, NCT01732861, and NCT01351935) showed that spebrutinib was safe and well-tolerated following once-daily administration in patients with relapsed or refractory CLL/SLL, WM, and NHL.^295^ Despite its high in vitro activity, spebrutinib exhibited inferior clinical efficacy compared with the approved BTK inhibitors. The reasons for the suboptimal effect are not fully understood, but the highly variable PK and pharmacodynamics (PD) seem to limit spebrutinib to continuously reach the in vivo targets.^296^ In addition, due to the critical role of BTK in the development and function of B cells, BTK has also been confirmed as a potential therapeutic target for autoimmune disorders. Several BTK inhibitors including evobrutinib,^297^ spebrutinib,^294^ branebrutinib, ^298^ and HM71224^299^ have been assessed in clinical trials for the treatment of autoimmune diseases, such as rheumatoid arthritis, systemic lupus erythaematosus, and remitting multiple sclerosis.

Both the first and second generations of BTK inhibitors covalently bind to the sulfhydryl group of Cys-481 in the active site of BTK. Cys-481 is reported to be the frequently mutated residue of BTK in cases of resistance to irreversible BTK inhibitors. Several mutants, including C481S, C481R, C481F, C481Y, and C481T, have been identified in resistant patients, especially C481S, which results in the vast majority of drug resistance.^300^ Moreover, PLCγ2 mutations have also been implicated in BTK inhibitor resistance. As a downstream protein of BTK, PLCγ2 mutations drive continued signaling regardless of BTK activity.^301^ Developing noncovalent BTK inhibitors is one of the available strategies to overcome resistance caused by Cys-481 mutations. Vecabrutinib,^302^ LOXO-305,^303^ fenebrutinib^304^ and ARQ-531^305^ are all noncovalent reversible inhibitors of BTK undergoing clinical evaluation. These agents inhibit BTK activity without forming covalent bonds with the Cys-481 residue; therefore, they have the same inhibitory effects on either wild-type BTK or its mutants. Another strategy is to target other components of the BCR pathway, such as inhibition of the upstream signaling pathways LYN or SYK, which can restrain BTK phosphorylation and conquer resistance caused by PLCγ2 mutations. In addition, the combined treatment of BTK inhibitors and other targeted agents is also considered a potential strategy for patients resistant to BTK inhibitor monotherapy.^306^

### JAK inhibitors

Janus kinases (JAKs) belong to the family of non-receptor tyrosine kinases and are composed of four isoforms, JAK1, JAK2, JAK3, and TYK2, with up to 70% homology.^307,308^ JAK1, JAK2, and TYK2 are widely distributed in various tissues and cells, while JAK3 is only expressed in the bone marrow and lymphatic-derived cells.^309^ JAKs are able to transfer extracellular signals to the nucleus and mediate DNA transcription and protein expression.^310^ Receptor-coupled JAKs can be activated when inflammatory cytokines such as interleukin and interferon bind to cytokine receptors.^311^ Then JAKs catalyze the phosphorylation of receptor tyrosine residues and recruit and phosphorylate downstream signal transducer and activator of transcription (STAT) proteins. Activated STAT proteins promote their translocation to the nucleus and regulation of target-gene transcription and expression. Distinct cytoplasmic domains of cytokine receptors activate different JAKs and STATs.^312^ The JAK/STAT pathway runs downstream of more than 50 cytokines and growth factors and is considered to be the central communication node for the immune system.^JA-7^ Given the important role of the JAK/STAT pathway in cytokine signal transduction, targeting JAK/STAT is considered a promising strategy for the treatment of multiple autoimmune diseases, such as rheumatoid arthritis and systemic lupus erythematosus. Additionally, STAT signals (e.g., STAT3, STAT5, or STAT6) have been found to be frequently activated in malignant tumors, especially hematopoietic cancers and are involved in cell proliferation, survival, invasion, or inflammation.^313^ As the critical upstream protein of STAT signals, JAKs are also potential targets for cancer treatment. The application of JAK inhibitors in cancer is mainly focused on hematologic malignancies.^314^

Thus far, four JAK inhibitors have been approved for clinical use. As shown in Table 2, ruxolitinib is the first launched JAK inhibitor and selectively targets JAK1 and JAK2 with moderate activity against TYK2. It was approved for the treatment of myelofibrosis (a myeloproliferative neoplasm), polycythemia vera, and bone marrow cancer in 2011 by the FDA.^315^ The recently approved fedratinib is a selective JAK2 inhibitor and is used for myelofibrosis treatment in the clinic.^316^ Meanwhile, it has been reported that fedratinib also showed efficacy in the treatment of NSCLC in preclinical studies. It can reverse the resistance of NSCLC cells to erlotinib and abrogate PD-L1 expression, which mediates immune checkpoint blockade therapy in NSCLC.^317^ Another two approved JAK inhibitors are tofacitinib and baricitinib.^318,319^ They are all used clinically for the treatment of autoimmune diseases.^313^

Recently, the role of several new JAK inhibitors in cancer treatment is undergoing clinical evaluation. WP1066 is a novel JAK2 and STAT3 inhibitor with little activity against JAK1 and JAK3. A phase I trial of WP1066 was performed to evaluate its effects in the treatment of melanoma and glioblastoma (NCT01904123).^320^ Gandotinib (LY2784544) is an orally potent inhibitor of JAK1 and JAK2.^274^ Clinical studies have demonstrated that gandotinib has adequate efficacy, safety, and tolerability profiles in patients with myeloproliferative neoplasms (NCT01594723). Lestaurtinib (CEP701) is a multikinase inhibitor targeting JAK2, FLT3, and neurotropin receptor TrkA. The use of lestaurtinib to treat multiple cancers, including AML, Hodgkin lymphoma, neuroblastoma, prostate cancer, and myeloproliferative disorders, has been reported in experimental or clinical studies.^321^ A phase II study (NCT00668421) exhibited moderate efficacy and moderate but frequent gastrointestinal toxicity of lestaurtinib in myelofibrosis patients.^322^ Further studies are still needed to assess its clinical benefits. INCB039110 is an effective JAK1 inhibitor with >20-fold selectivity over JAK2 and >100-fold over JAK3 and TYK2.^323^ In an open-label phase II trial (NCT01633372), myelofibrosis-related symptoms were obviously ameliorated after INCB039110 treatment. Moreover, pacritinib (SB1518) is a dual JAK2 and FLT3 inhibitor that can inhibit JAK2 and JAK2 V617F mutations as well as FLT3 and FLT3-D835Y mutations. It has been clinically tested in myelofibrosis and AML patients (NCT03645824, NCT02532010), and has shown efficacy for myelofibrosis therapy.^324^

JAK inhibitors are effective for the management of immune‐mediated diseases (rheumatoid arthritis, ulcerative colitis, and psoriatic arthritis),^313^ myelofibrosis,^325^ and polycythaemia vera; these diseases generally respond well to JAK inhibitors. A large number of clinical trials related to JAK inhibitors are still in progress.^326^ With further understanding of the clinical potential of JAK inhibitors, their indications will also be expanded.^327^ For example, the combination of JAK inhibitors with PD-L1 monoclonal antibodies or inhibitors of relevant kinases such as STAT inhibitors in cancer treatment has a certain theoretical basis and data support.^328^ In addition, side effects should be considered in the clinical use of JAK inhibitors. The adverse reactions related to JAK inhibitors include infection, neutropenia, venous thromboembolism, anemia, hypercholesterolemia, and even malignancy. Susceptibility to various infections is the main side effect after treatment with these inhibitors. Therefore, reliable prevention and monitoring strategies are needed during the medication process.^329,330^

## Serine/theonine kinase inhibitors

### BRAF/MEK/ERK inhibitors

Rat sarcoma virus (RAS)-rapidly accelerated fibrosarcoma (RAF)-mitogen-activated protein kinase (MAPK)-extracellular signal-regulated kinase (ERK) signaling is a well-established pathway that controls cell growth, proliferation, and survival in normal cells and cancer cells.^331,332^ Core components of RAS-RAF-MAPK-ERK signaling include the small GTPase RAS, the serine/threonine kinase RAF, the protein kinases MEK1/2 and ERK1/2. RAF, including ARAF, BRAF, and CRAF, is the direct downstream of RAS, which serves as a transducer of extracellular stimuli.^332^ RAF activates the dual-specificity protein kinase MEK1/2, which subsequently phosphorylates ERK1/2. Activated ERK phosphorylates multiple substrates in the cytosol and nucleus, and then promote cell proliferation and survival.^332^ Dysregulation of RAS/RAF/MEK/ERK signaling can be observed in a large number of cancers, which is most commonly due to mutations in RAS or BRAF.^332,333^ BRAF mutations (mostly BRAF V600E) have been identified in ~40–50% of melanomas and 6% of other malignancies, which results in several-fold hyperactivation of BRAF and continuous activation of downstream MEK and ERK.^333,334^ Due to the picomolar affinities of RAS to GTP, RAS was taken as an undruggable target until the appearance of irreversible small-molecule inhibitors of KRAS G12C in recent years.^335^ Therefore, BRAF and the downstream kinases MEK1/2 and ERK are considered attractive therapeutic targets for malignant tumors, especially melanoma.

Small-molecule BRAF inhibitors can be classified into two types. Type-I inhibitors stabilize the kinase in its active (DFG-in) conformation by occupying the ATP-binding pocket.^336^ The FDA has approved three type-I competitive BRAF inhibitors for the treatment of non-resectable BRAF V600E/K/D mutant melanoma as single agents or combined with MEK inhibitors including vemurafenib in 2011,^337^ dabrafenib in 2013,^338^ and encorafenib in 2018 (Table 3).^339^ Type-II BRAF inhibitors bind to the adjacent hydrophobic sites of ATP-binding pocket and stabilize the kinase in its inactive (DFG-out) conformation.^336^ Sorafenib, a representative type-II pan-RAF inhibitor, is a multikinase inhibitor. This agent was initially developed as a RAF kinase inhibitor; however, it also showed inhibitory activity against VEGFR-1/2/3, PDGFR, FLT1, KIT, and RET. It is currently approved by FDA for the treatment of unresectable HCC, advanced RCC, and thyroid carcinoma refractory to radioactive iodine, but not for melanoma due to the unsatisfactory results of clinical trials.^340^

**Table 3:** Properties of approved small-molecule inhibitors of serine/theonine kinases

Chemical structure	Name	Targets	Approved indications (year)	Corporation
	Vemurafenib (Zelboraf)	BRAF	Melanoma (2011)	Roche/Genentech
	Dabrafenib (Tafinlar)	BRAF/CRAF	Melanoma (2013) NSCLC (2017) ATC (2018)	Novartis/GlaxoSmithkline
	Encorafenib (Braftovi)	BRAF	Melanoma (2018)	Array
	Trametinib (Mekinist)	MEK1/2	Melanoma (2013) NSCLC (2017) ATC (2018)	Novartis/GlaxoSmithkline
	Cobimetinib (Cotellic)	MEK1/2	Melanoma (2015)	Roche/Genentech/ Exelixis
	Binimetinib (Balimek)	MEK1/2	Melanoma (2018)	Array
	Selumetinib (Koselugo)	MEK1/2	Neurofibromatosis type 1 (2020) Plexiform neurofibromas (2020)	AstraZeneca/Merck
	Palbociclib (Aiboxin)	CDK4/6	Breast cancer (2015)	Pfizer
	Ribociclib (Kisqali)	CDK4/6	Breast cancer (2017)	Novartis
	Abemaciclib (Verzenio)	CDK4/6	Breast cancer (2017)	Lilly
	Idelalisib (Zydelig)	PI3Kδ	CLL (2014) Follicular lymphoma (2014)	Gilead
	Copanlisib (Aliqopa)	Pan-PI3K	Follicular lymphoma (2017)	Bayer
	Duvelisib (Copiktra)	PI3Kγ/δ	CLL (2018) SLL (2018) Follicular lymphoma (2018)	Verastem
	Alpelisib (Piqray)	PI3Kα	Breast cancer (2019)	Novartis
	Temsirolimus (Torisel)	mTOR	RCC (2007)	Pfizer
	Everolimus (Afinitor)	mTOR	RCC (2009) Pancreatic cancer (2011) Breast cancer (2012)	Novartis
	Sirolimus (Rapamune)	mTOR	LAM (2015)	Pfizer

The discovery and use of BRAF inhibitors opened up an avenue for the development of inhibitors of MEK1/2 and ERK, downstream targets of RAS/RAF/MEK/ERK signaling. Three MEK1/2 inhibitors have been approved in combination with BRAF inhibitors for the treatment of unresectable or metastatic melanoma harboring BRAF V600E/K mutation including reversible allosteric inhibitors trametinib and binimetinib as well as cobimetinib, a highly selective inhibitor that restrains the catalytic activity of MEK1/2 (Table 3); trametinib is also administered as a monotherapy.^341^ The FDA-approved combination regimens of BRAF and MEK inhibitors (vemurafenib plus cobimetinib, dabrafenib plus trametinib, and encorafenib plus binimetinib) can achieve a longer PFS in patients with melanoma compared with BRAF inhibitor monotherapy in clinical trials, and encorafenib-binimetinib combination had the best toxicity profile.^342^ The combination of dabrafenib and trametinib has also been approved for the treatment of NSCLC or anaplastic thyroid cancer (ATC) patients harboring BRAF V600E mutation. Moreover, the MEK inhibitor binimetinib (as monotherapy or in combination therapy) has been reported to be effective for patients with NRAS mutant melanoma.^343^ Recently, the FDA-approved a novel MEK1/2 inhibitor named selumetinib. It is a highly selective allosteric inhibitor of MEK1/2 and was initially assessed in patients with metastatic uveal melanoma. It has been granted orphan drug status as adjuvant treatment for thyroid cancer and as treatment for neurofibromatosis type 1. Based on the results of the phase II SPRINT trial, selumetinib was approved for pediatric patients with neurofibromatosis type 1 and symptomatic, inoperable plexiform neurofibromas in April 2020 (Table 3).^344^ Currently, no ERK inhibitor has been approved for clinical use.

The success of RAS/MAPK signaling inhibitors in the treatment of melanoma has promoted the further development of such inhibitors, and a variety of novel inhibitors have entered clinical trials.^345,346^ Typically, pimasertib (AS703026) is a highly selective ATP non-competitive allosteric inhibitor of MEK1/2. A phase II trial comparing the combination of pimasertib with SAR245409 (PI3K inhibitor) to pimasertib alone in patients with recurrent unresectable borderline or low-grade ovarian cancer has been completed (NCT01936363). Response to pimasertib alone (ORR: 12%) was as effective as the combination, suggesting that MEK inhibition could be used as an alternative treatment method to cytotoxic chemotherapy in this population.^347^ RO5126766 is a dual RAF/MEK allosteric inhibitor with a novel structure based on coumarin skeleton. A phase I clinical trial (NCT02407509) is conducted to assess RO5126766 alone or together with everolimus on patients with solid tumors or MM harboring BRAF, NRAS, and/or KRAS mutation;^348^ the completion date of this trial is targeted for June 2020. The pyrazole amino-pyrimidine derivative ravoxertinib (GDC-0994) is a selective ERK1/2 inhibitor with an IC_50_ in the subnanomolar range. Preclinical studies have demonstrated that ravoxertinib had good PD and PK properties. Interestingly, it also showed strong antitumor activity in nude mice harboring KRAS-mutant xenografts.^349^ The efficacy and safety of ravoxertinib in combination with cobimetinib in the treatment of locally advanced or metastatic solid tumors were evaluated in a phase I study (NCT02457793), but the data are not yet available.

The clinical application of BRAF and MEK inhibitors has changed the prospect of melanoma treatment, especially the combination therapy with both agents. Treatment with BRAF inhibitors is often accompanied by paradoxical MAP kinase reactivation that limits the clinical response.^350^ The combination with MEK inhibitors alleviates this situation to some extent, thereby significantly improving the response rate and survival of patients with melanoma. However, the efficacy of this therapy is limited by acquired resistance caused by gene mutation and bypass activation.^351^ Some novel combinations of targeted therapy are expected to improve efficacy and overcome drug resistance, such as the combination with ERK inhibitors, CDK4/6 inhibitors, or inhibitors of the PI3K/AKT/mTOR pathway.^352^ In addition, resistance to BRAF and MEK inhibitors is driven partly by immune-mediated mechanisms; therefore, combined targeted therapy and immunotherapy have become the focus in clinical melanoma treatment in recent years.^351^ An array of such trials is under assessment for toxicity, efficacy, and treatment sequence to support the continuous progress of individualized treatment of melanoma.^351,352^

### CDK inhibitors

Cell cycle abnormalities result in uncontrolled cell proliferation and have been considered one of the important hallmarks of cancer.^353^ Cyclin-dependent kinases (CDKs) are critical enzymes regulating cell cycle progression and require cyclin proteins for activation and downstream phosphorylation.^354^ To date, at least 20 CDKs and 29 cyclins have been identified in humans.^355^ Among them, CDK4 and CDK6 are necessary for regulating growth signaling and driving the transition of the cell cycle from G1 to S phase. During the process of regulation, CDK4 and CDK6 are activated by D-type cyclins, which induce the phosphorylation of tumor suppressor retinoblastoma protein 1 (RB1) early in the G1 phase. This leads to the inactivation of RB and release of E2F transcription factors, thereby promoting the transcription of target genes related to cell cycle progression.^356–358^ Given the critical role of the CDK4/6-RB1 axis in mediating cellular proliferation and tumorigenesis, inhibition of CDK4/6 is a promising strategy for cancer therapy that can induce cell cycle arrest in G1 phase and result in decreased cell viability.^359^ Notably, CDK4 and CDK6 are functionally identical in their biological effects; therefore, dual inhibition of CDK4 and CDK6 is essential because of the compensatory effects. Cyclin D, the catalyst for CDK4/6, is a major transcriptional target of the estrogen receptor (ER). Estrogen binding to the ER initiates cyclin D transcription, followed by activation of the CDK4/6-RB1 pathway.^360–362^ Hence, dysregulation of the CDK4/6-RB1 pathway is a significant feature of hormone receptor (HR)-positive breast cancers.^363^

In the past three decades, great progress has been made in the development of CDK inhibitors.^364^ However, many early developed non-selective and pan-CDK inhibitors (e.g., flvopiridol, seliciclib, UCN-01) have been discontinued due to their limited clinical efficacy or serious side effects. To date, three CDK inhibitors are clinically available: palbociclib,^365^ ribociclib,^366^ and abemaciclib (Table 3).^367^ All of them have similar chemical structures and are orally selective reversible inhibitors specifically targeting CDK4/6. They are approved for the treatment of metastatic HR-positive, HER2-negative breast cancer in combination with specific endocrine therapies.^368^ In contrast, palbociclib and ribociclib are used in combination with non-steroidal aromatase inhibitors (e.g., letrozole) in postmenopausal women with HR-positive HER2-negative metastatic breast cancer,^369^ and palbociclib is also combined with fulvestrant for breast cancer patients with disease progression following endocrine therapy.^370^ Abemaciclib shares an indication with palbociclib for use in combination with fulvestrant in HR-positive HER2-negative breast cancer progressing after endocrine therapy.^371^ In particular, it is also the only CDK4/6 inhibitor approved as monotherapy for HR-positive HER2-negative metastatic breast cancer pretreated with endocrine therapy and chemotherapy.^372^ Despite being approved only for specific breast cancer, the three approved CDK4/6 inhibitors have also been assessed in other solid tumors and hematologic malignancies, such as lung cancer, prostate cancer, melanoma, glioblastoma, and myelofibrosis, and have shown promising results in preclinical studies or clinical trials of some malignancies.^373,374^ Nevertheless, further clinical research is needed to confirm their efficacies.

Additionally, several newly developed CDK4/6 inhibitors have entered clinical trials. Trilaciclib (G1T28) inhibits CDK4 and CDK6 with IC_50_ values of 1 and 4 nM, respectively. It has been evaluated clinically for the prevention of chemotherapy-induced myelosuppression in triple-negative breast cancer (TNBC) and SCLC.^375^ The pyridopyrimidine derivative PF-06873600 is potent against CDK2/4/6 and can overcome palbociclib resistance in preclinical studies. Phase II trials of PF-06873600 in the treatment of metastatic breast cancer and other gynecological cancers are ongoing (NCT03519178). SHR-6390 conquered resistance to tamoxifen or trastuzumab, and its combination with endocrine therapy had significant synergistic effects in breast cancer.^376^ BPI-16350, FCN-437, and XZP-3287 can effectively penetrate the blood–brain barrier. These CDK4/6 inhibitors are currently being studied in phase I/II trials (NCT03791112, NCT04488107, and NCT04539496).^364^ A variety of reversible ATP-competitive inhibitors of other CDK isoforms have been evaluated in the clinic in combination with standard-of-care agents or as monotherapy, such as the CDK2 inhibitors inditinib (AGM-130)^377^ and FN-1501,^378^ the selective CDK7 inhibitor ICEC0942,^379^ the CDK9 inhibitors BAY-1251152,^364^ and CYC-065, a second-generation inhibitor of CDK2/5/9. Meanwhile, with the development of modern biotechnology, non-classical CDK inhibitors (allosteric inhibitors, covalent inhibitors, and PROTACS) have laid the foundation for the discovery of a novel generation of selective CDK inhibitors. Notably, SY-1365 is an ATP-competitive covalent inhibitor of CDK7 and is the first irreversible CDK inhibitor entering the clinical study.^380^ However, the development of this drug was discontinued in September 2019 by Syros Pharmaceuticals due to poor clinical data, which showed that sustaining CDK7 target coverage levels to enhance clinical activity would require more frequent doses or a higher dose, and this may lead to an overly burdensome dosing schedule for patients.^364^

Attention should be paid to the adverse events related to the approved CDK4/6 inhibitors. Hematological toxicities such as neutropaenia are often observed in the clinical use of palbociclib and ribociclib,^359^ while gastrointestinal toxicities, especially diarrhea, are common in abemaciclib treatment.^368^ Most adverse reactions are normally easily controlled by standard supportive care and dose adjustments. In September 2019, the FDA issued a drug safety newsletter and warned that palbociclib, ribociclib, and abemaciclib used to treat advanced breast cancer could trigger rare but serious pneumonia called interstitial lung disease.^381^ This risk warning information must be added to the drug labels and patient instructions of all approved CDK4/6 inhibitors.^382^ Moreover, understanding the mechanisms of resistance to CDK4/6 inhibitors is another issue that needs to be considered. RB1 is the phosphorylation target of CDK4/6; therefore, loss of RB1 expression is one of the potential mechanisms leading to CDK4/6 inhibitor resistance. RB1 mutations have been detected in the ctDNA of breast cancer patients resistant to CDK4/6 inhibitors, with an estimated frequency of 5%.^383^ Ectopic overexpression of cyclin E1 (CCNE1) and cyclin E2 (CCNE2) can also result in resistance to antioestrogen and palbociclib monotherapy through activation of CDK2.^384^ In addition to cell cycle alterations, overexpression or mutation in upstream proteins, including AKT1, KRAS/HRAS/NRAS, ERBB2, and FGFR2, have also been observed as potential resistance mechanisms. Given these findings, exploring rational combination treatment strategies may be efficacious to conquer CDK inhibitor resistance.^385^

### PI3K/AKT/mTOR inhibitors

The phosphatidylinositol 3-kinase (PI3K)/V-AKT murine thymoma viral oncogene homolog (AKT)/mammalian target of rapamycin (mTOR) signaling pathway has an important role in cell growth, proliferation, survival, apoptosis, and motility and is frequently activated in human cancer.^353^ PI3Ks are a family of lipid kinases that catalyze the phosphorylation of phosphatidylinositol D3. According to the structural characteristics of the subunits and substrates, PI3Ks can be divided into three classes. Of these, class I PI3K is the major isoform implicated in cancer and can be further subdivided into class IA and class IB, which are activated by RTKs and GPCRs, respectively. Class IA consists of PI3Kα, PI3Kβ, and PI3Kδ encoded by *PIK3CA*, *PIK3CB*, and *PIK3CD* genes, respectively. Class IB comprises only the PI3Kγ subtype encoded by *PIK3CG.*^386,387^ PI3K/AKT/mTOR pathway is activated by various mechanisms in oncogenesis and progression. *PIK3CA* gene is frequently dysregulated in multiple cancers, both by point mutations and amplification. The tumor suppressor PTEN negatively regulates the PI3K pathway by dephosphorylating PIP3 to PIP2. It is also mutated frequently and results in loss of function in human cancers, which upregulates PIP3 levels and leads to constitutive activation of AKT and downstream components. Moreover, as the immediate downstream effector of PI3K, amplification and activating mutations of *AKT* are often observed in solid tumors.^388^ Hyperactivation of PI3K/AKT/mTOR signaling is not only common in a variety of tumors but also closely related to drug resistance (e.g., resistance mechanism of EGFR inhibitors); thus, this pathway has become an attractive target for developing antitumor targeted drugs. Many small-molecule inhibitors of PI3K, AKT, and mTOR have been developed in the past few years. However, only several PI3K and mTOR inhibitors have been approved for cancer treatment.

The early developed PI3K inhibitors are mostly pan-PI3K inhibitors that are capable of binding to all class I PI3Ks, such as wortmannin and LY294002. However, due to their poor PK properties, they are not ultimately approved for clinical use. The second-generation isoform-selective PI3K inhibitors are highly selective for the four isoforms of the class I PI3K catalytic subunit p110 (α, β, γ, and δ). As indicated in Table 3, idelalisib is the first approved selective PI3Kδ inhibitor based on its efficacy in the treatment of relapsed or refractory CLL patients. It is also recommended for the treatment of lymphocytic lymphoma patients who have received at least two prior systemic therapies or patients with relapsed follicular B-cell NHL.^389^ Copanlisib, approved in September 2017, is a pan-PI3K inhibitor with IC_50_ values of 0.5, 3.7, 6.4, and 0.7 nM against class I PI3K-α, β, γ, and δ isoforms, respectively.^390^ It is used clinically for the treatment of adult patients with relapsed follicular lymphoma who have received at least two prior systemic therapies. Duvelisib is an oral dual PI3Kγ and PI3Kδ inhibitor with IC_50_ values of 27 and 2.5 nM, respectively.^391^ It prevents the activation of PI3K-γ and δ isoforms by competitively and reversibly binding to the ATP-binding pocket of the p110 subunit.^392^ The FDA has approved duvelisib for the treatment of adult patients with relapsed or refractory CLL, SLL, and follicular lymphoma after at least two prior therapies. The newly approved alpelisib is a selective inhibitor targeting the α-isoform of class I PI3K, with an in vitro IC_50_ of 4.6 nM. It is indicated in combination with fulvestrant for the treatment of postmenopausal women and men with HR-positive, HER2-negative, *PIK3CA*-mutated, advanced, or metastatic breast cancer as detected by an FDA-approved test following progression on or after an endocrine-based regimen.^393^ This is also the first PI3K inhibitor approved for clinical treatment of breast cancer. In addition, there are also a variety of PI3K inhibitors undergoing clinical evaluation, such as the pan-class I PI3K inhibitors XL147 and ZSTK474, PI3Kγ inhibitor IPI-549, and PI3Kα inhibitor serabelisib.^394–397^ Some of them have progressed to phase II or III trials and have good prospects for approval. In addition, the promising efficacy of PI3K inhibitors raised the question of whether the combined inhibition of multiple pathway components could further improve the efficacy without excessive side effects. Developing dual PI3K/mTOR inhibitors is a predominant strategy due to the high homology between the ATP-binding domain of p110 and the catalytic site of mTOR. Several dual PI3K/mTOR inhibitors have entered clinical trials, such as bimiralisib, dactolisib (BEZ235), GDC-0084 (RG7666), and gedatolisib (PKI-587/PF-05212384).^398–400^ Current clinical trials focus on combining them with a range of other antitumor agents. Notably, GDC-0084 is orally administered and can penetrate the BBB. It is currently specifically developed for patients with glioma or brain metastases from solid tumors.

Compared with PI3K and mTOR inhibitors, the development of AKT inhibitors is relatively slow. The obtained results from numerous preclinical studies revealed that AKT inhibitors are potent for tumors with *PIK3CA* mutations or loss of PTEN function.^386^ However, most of these agents displayed limited clinical effects in patient settings, especially as monotherapy. Only several AKT inhibitors, including ATP-competitive inhibitors and allosteric inhibitors, have progressed to clinical trials. The ATP-competitive inhibitors ipatasertib, capivasertib, afuresertib, and uprosertib are all highly selective pan-AKT inhibitors and can prevent AKT activation and enhance the antitumor activity of chemotherapeutic drugs.^401,402^ A phase III trial (NCT03072238) of ipatasertib is being conducted to evaluate the efficacy of its combination with abiraterone and prednisone/prednisolone in adult male patients with metastatic castration-resistant prostate cancer (CRPC). Three other phase III trials related to ipatasertib combination are actively recruiting patients with HR-positive and HER2-negative locally advanced unresectable or metastatic breast cancer (NCT04060862, NCT04177108, and NCT03337724). The efficacy of capivasertib (AZD5363) combined with paclitaxel as a first-line treatment for locally advanced or metastatic TNBC patients is under evaluation in a phase III trial (NCT03997123).^403^ Afuresertib (GSK2110183) monotherapy showed satisfactory safety and clinical activity against hematological malignancies, including MM.^404^ Uprosertib (GSK2141795) was used for relapsed or refractory MM either alone or in combination with trametinib in phase I and II clinical trials (NCT01989598, NCT01979523).^405^ In addition, allosteric AKT inhibitors are represented by MK-2206. Several clinical trials are ongoing to test MK-2206 monotherapy or in combination with targeted therapy or chemotherapy for metastatic breast cancer and in combination with lapatinib and trastuzumab for HER2-positive breast cancer (NCT01277757, NCT01245205, and NCT01235897). Notably, there is currently an AKT inhibitor approved for marketing, named miltefosine. However, it is used clinically for the treatment of visceral and cutaneous leishmaniasis but not for malignancies.

mTOR inhibitors can be divided into two categories: rapamycin analogs (rapalogs) and ATP-competitive inhibitors. The former is the first-generation mTOR inhibitor, which forms a complex with FK506 binding protein 12 (FKBP12) and inhibits the activity of only mTORC1 but not mTORC2.^406^ They are also the first compounds developed to target the PI3K/mTOR pathway. Currently, multiple rapalogs, such as sirolimus (rapamycin), temsirolimus, and everolimus, have been approved for the treatment of various cancers, including RCC, breast cancer, and pancreatic cancer (Table 3). Of these, rapamycin was initially approved as an immunosuppressant for transplant patients, and its indications were subsequently extended to lymphangioleiomyomatosis (LAM).^407^ Although patients can benefit from such medications, drug resistance caused by mTORC2-mediated negative feedback limits their clinical use. Second-generation mTOR inhibitors are designed as ATP-competitive inhibitors, which can simultaneously suppress the activity of both mTORC1 and mTORC2. At present, no such inhibitors have received approval, but some of them are under clinical evaluation, such as sapanisertib (TAK-228), vistausertib (AZD2014), CC-223, BI860585, DS-3078a, ME-344, GDC-0349, OSI-027, and P529.^406^ Representatively, sapanisertib (TAK-228), formerly known as INK128 or MLN0128, is an orally effective mTOR inhibitor with an IC_50_ of 1 nM.^408^ It is being evaluated for the treatment of multiple cancers in several phase II trials (NCT02484430, NCT03047213, and NCT02893930). Vistusertib is a selective mTOR inhibitor with an IC_50_ of 2.8 nM. In preclinical studies, vistusertib exhibited a broad spectrum of antitumor activity.^409^ It is undergoing evaluation in a phase II clinical trial for the treatment of recurrent grade II–III meningiomas (NCT03071874).

Isoform-specific inhibitors have fewer off-target effects and side effects than pan-PI3K, pan-AKT, and dual PI3K/mTOR inhibitors. However, due to their limited therapeutic efficacy caused by compensation effects or various mutations of the PI3K/AKT/mTOR pathway, most clinical trials still tend to use pan-inhibitors. Therefore, clarifying the mutant types via molecular pathological diagnosis in advance can improve the clinical application of specific inhibitors. On the other hand, the clinical efficacy using only PI3K/AKT/mTOR inhibitors were shown to have modest effects for patients in actual treatment. More importantly, PI3K/AKT/mTOR cascade exchanges crosstalk with many other signaling pathways, such as Wnt and MAPK signaling. Hence, using such inhibitors is prone to negative feedback regulation, resulting in resistance. These problems highlight the limitations of PI3K/AKT/mTOR inhibitors as monotherapy in malignancies. Currently, efforts have focused on combination therapy, including inhibition of parallel pathways as well as targeted therapy combined with cytotoxic drugs,^388^ and the results of most clinical trials are promising. Moreover, treatment with PI3K pathway inhibitors could also induce adverse effects, even though isoform-specific inhibitors are no exception. For example, the PI3Kδ inhibitor idelalisib was shipped with four black-frame warnings, suggesting fatal and severe liver toxicity, diarrhea and colitis, pneumonia, and intestinal perforation.^410^ Other toxicities reported in clinical trials of the PI3K/AKT/mTOR inhibitors include immunosuppression, hypoglycemic, cardiac toxicity, neuropsychiatric effects, cutaneous reactions, nausea, mouth ulcers, constipation, etc. A better understanding of the mechanism of side effects caused by such drugs and their management might advance novel PI3K/AKT/mTOR inhibitors from clinical trials to the bedside, with new treatment options for patients.

We in the above summarize the anti-cancer kinase inhibitors approved by the US FDA and NMPA of China. To date, a total of 70 kinase inhibitors have been approved to use in the clinic, which accounts for almost 80% of all approved small-molecule targeted anti-cancer drugs (89 in total). Despite this great achievement, these kinase inhibitors just act on a small number of kinase targets (<30 kinases). Many kinases that are thought of as potential anti-cancer targets still lack inhibitors or at least potent and selective inhibitors. Therefore, drug discovery targeting kinases still has a lot of room for development. Another hot topic of debate regarding kinase inhibitors is whether single-target drugs or multitarget drugs have more advantages. Target-specific kinase inhibitors generally have low toxicity but are easy to develop resistance. While multikinase inhibitors often have high side effects, but have advantages in anti-cancer efficacy and overcoming drug resistance.

## Epigenetic inhibitors

**Fig. 3:**
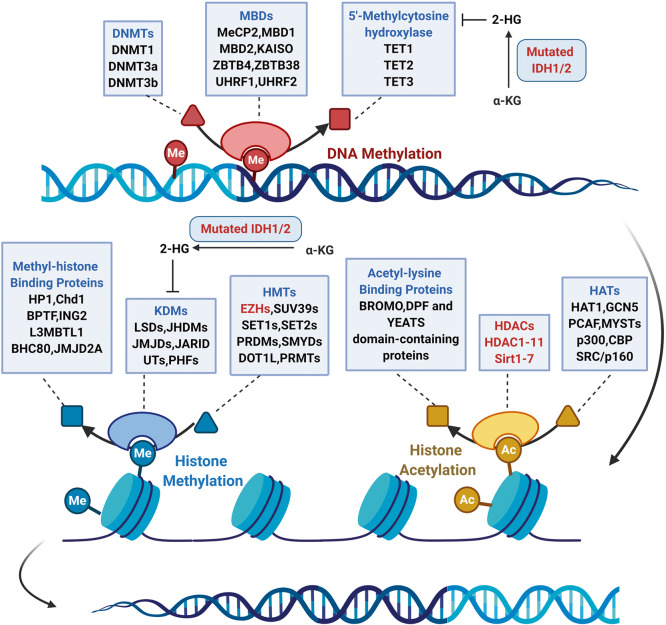
Commonly altered epigenetic regulatory proteins implicated in cancer. Gene silencing in mammalian cells is usually caused by methylation of DNA CpG islands as well as hypermethylation or hypoacetylation of histones. The writers (DNMTs, HATs, and HMTs) refer to enzymes that transfer chemical groups to DNA or histones; the erasers (HDACs and KDMs) are enzymes responsible for removing chemical groups from histones; the proteins (MBDs and BET family proteins) that can recognize the methyl-CpGs and modified histones are readers. Mutated IDH1/2 catalyzes the reduction of α-KG to 2-HG, which inhibits the activity of TET and lysine demethylases, resulting in DNA hypermethylation and increased histone lysine methylation. Figure created with BioRender.com

Epigenetics is a branch of genetics that studies the heritable changes of gene expression without changing the nucleotide sequence of genes. It is strictly regulated by a variety of chemical modifying enzymes and recognition proteins, which are often called “writers”, “erasers”, and “readers”.^411,412^ The writers refer to enzymes that transfer chemical groups to DNA or histones, which include DNA methyltransferases (DNMTs), histone acetyltransferases (HATs), and histone lysine methyltransferases (KMTs). The erasers remove post-translational modifications, and include histone deacetylases (HDACs) and histone lysine demethylases (KDMs). The readers are proteins that can recognize the modified histones or DNA, such as methyl-binding domain proteins, and bromodomain and extra-terminal (BET) family proteins (Fig. 3). Abnormal epigenetic regulation is also closely related to various diseases including tumor, immune diseases, and many rare diseases. Though numerous epigenetic regulatory proteins have been identified as potential disease targets, only fewer epigenetic drugs are approved for clinical use at present.

### EZH2 Inhibitors

Enhancer of zeste homolog 2 (EZH2), a histone methyltransferase, functions as a catalytic subunit of the polycomb repressor complex 2 (PRC2), which also comprises other members including embryonic ectoderm development (EED), suppressor of zeste 12 (SUZ12), and histone-binding proteins RbAp46/48.^413^ PRC2 is one of the two core complexes of polycomb group proteins (PcGs), and is responsible for transferring methyl groups from S-adenosyl-L-methionine (SAM) to lysine 27 on histone H3 (H3K27) through its C-terminal SET domain, resulting in chromatin compaction and transcriptional silencing of target genes. As the central component of PRC2, EZH2 is involved in numerous epigenetic modifications that are associated with cell proliferation, differentiation, survival, adhesion, and DNA damage repair.^414^ Dysfunction of EZH2 is closely related to tumorigenesis and progression. Accumulating evidence has confirmed that EZH2 is frequently mutated and abnormally overexpressed in various malignant tumors including prostate cancer,^415,416^ ovarian cancer,^417^ endometrial carcinoma,^418^ breast cancer,^419^ melanoma as well as hematological malignancies,^420^ such as NHL, B-cell lymphoma, and T-cell ALL.^421–424^ It promotes tumorigenesis mainly through three mechanisms: PRC2-dependent H3K27 methylation, PRC2-dependent non-histone protein methylation, and PRC2-independent coactivator of transcriptional factors. Given the evidence for EZH2 enzymatic gain of function being a cancer driver, inhibition of EZH2 has been thought of as a novel and promising approach for cancer therapy.^413^

The first reported small-molecule EZH2 inhibitor is 3-deazaneplanocin A (DZNep), a cyclopentanyl analog of 3-deazaadenosine. This compound can potently inhibit the *S*-adenosyl-l-homocysteine (SAH) hydrolase activity and induce the increase of cellular 5-adensylhomocystein levels, thus suppressing the activity of global *S*-adenosyl-l-methionine (SAM)-dependent histone lysine methyltransferase, including EZH2-mediated histone methylation. Therefore, DZNep is a non-specific EZH2 inhibitor.^425^ Although treatment with DZNep showed significant antitumor activity in various preclinical studies, the poor PK profile and safety encouraged further development of more potent and selective EZH2 inhibitors. Since 2012, multiple SAM-competitive EZH2 inhibitors have been reported, including EI1, EPZ005687, GSK126, GSK343, UNC1999, tazemetostat (EPZ6438), SHR2554, CPI-1205, DS-3201, PF-06821497, and HH2853 (NCT04390737).^413^ These compounds display better selectivity for EZH2 or EZH1 compared with other methyltransferases, and most of them bind to both wild-type and mutant EZH2, particularly Y641 and Y646 mutations.^426,427^ Currently, several of these inhibitors (e.g., tazemetostat, SHR2554, CPI-1205, DS-3201, PF-06821497, and HH2853) have entered clinical trials to evaluate their clinical efficacy and safety in a variety of solid tumors or hematological malignancies. Among them, tazemetostat developed by Epizyme and Eisai is an oral competitive inhibitor of the SAM pocket of the EZH2 SET domain, and received accelerated approval in January 2020 in the USA for the treatment of adults and adolescents aged ≥16 years with locally advanced or metastatic epithelioid sarcoma not eligible for complete resection (Table 4). Tazemetostat is the first approved EZH2 inhibitor and also the first therapy specifically for the treatment of epithelioid sarcoma.

**Table 4:** Properties of approved small-molecule inhibitors of epigenetic targets

Chemical structure	Name	Targets	Approved indications (year)	Corporation
	Tazemetostat (Tazverik)	EZH2	Epithelioid sarcoma (2020)	Epizyme/Eisai
	Vorinostat (Zolinza)	HDAC1/2/3/6	CTCL (2006)	Merck
	Romidepsin (Istodax)	HDACs	CTCL (2010) PTCL (2011)	Celgene
	Belinostat (Beleodaq)	HDAC1/2	PTCL (2014)	Onxeo/Spectrum
	Tucidinostat (Epidaza)	HDAC1/2/3/10	PTCL (2015)	Chipscreen Biosciences
	Panobinostat (Farydak)	HDACs	MM (2015)	Secura Bio
	Enasidenib (Idhifa)	IDH2	AML (2017)	Agios/Celgene
	Ivosidenib (Tibsovo)	IDH1	AML (2018)	Agios

In addition to the highly selective SAM-competitive inhibitors, EZH2 can also be inhibited by disrupting its interaction with other PRC2 subunits. EED226 (MAK683) is a potent and selective PRC2 allosteric antagonist, which directly binds to the H3K27me3 pocket of EED and induces a conformational change, therefore disrupting EZH2-EED interaction and resulting in loss of PRC2 activity.^428^ EED226 shows similar activity to SAM-competitive inhibitors in blocking H3K27 methylation of PRC2 target genes and inducing regression of human lymphoma xenograft tumors. Interestingly, EED226 could also potently inhibit the activity of PRC2 containing a mutant EZH2 protein resistant to SAM-competitive inhibitors.^428^ A phase I trial (NCT02900651) is ongoing to evaluate the safety and efficacy of EED226 in patients with advanced malignancies, such as DLBCL, nasopharyngeal carcinoma (NPC) or other advanced solid tumors for whom no further effective standard treatment is available. Besides, some other small-molecule inhibitors, such as A-395,^429^ BR-001, and UNC6852, were also reported as EED inhibitors that inhibit the interaction between EZH2 and EED and destabilize PRC2 complex,^430,431^ thereby exerting antitumor activity. Moreover, Wang et al. developed a unique strategy to target EZH2 by protein degradation.^432^ They identified a gambogenic acid (GNA) derivative GNA002 that could specifically and covalently bind to Cys668 within the EZH2-SET domain and trigger EZH2 degradation through COOH terminus of Hsp70-interacting protein (CHIP)-mediated ubiquitination. In this study, GNA002 significantly suppressed H3K27Me3 and effectively reactivated PRC2-silenced tumor suppressor genes, and could inhibit tumor growth in an EZH2-dependent manner.^432^

The efficacy of EZH2 inhibitors is limited by both primary and acquired resistance, which is the main challenge for the clinical use of EZH2 inhibitors. Primary resistance is often due to EZH2 resistant mutations, differences in the tumor microenvironment, heterogeneity of tumor tissue structure (influencing PKs and drug delivery), or activation of prosurvival pathways as reported in DLBCL and SWI/SNF mutant cancer cells.^433,434^ Huang et al. profiled global post-translational histone modification changes across a large panel of cancer cell lines with various sensitivities to EZH2 inhibitors, and found that oncogenic transcriptional reprogramming mediated by MLL1’s interaction with the p300/CBP complex directed H3K27me loss to reciprocal H3K27ac gain and restricted response to EZH2 inhibition.^435^ This resistance could be effectively reversed by concurrent inhibition of H3K27me and H3K27ac with a combination of EZH2 and BRD4 inhibitors. Acquired resistance is usually because of multiple secondary mutations of EZH2, such as Y641F, C663Y, E720G, Y726F, Y111L, and Y661D.^433,434,436,437^ Combination therapy is thought as the most efficient strategy to improve the limited effectiveness of EZH2 inhibitors.^438^ For example, the approved drug tazemetostat is currently assessed clinically in several combined treatments for a variety of malignant tumors. Exploring various effective combination strategies, including combination with chemotherapy, immunotherapy, kinase targeted therapy, and metabolic regulators, is a promising direction in the near future.^414,439,440^

### HDAC inhibitors

Histone deacetylases (HDACs) are important epigenetic regulators that remove the acetyl groups from the N-acetylated lysine residues of histones and various non-histone substrates. To date, 18 HDACs have been identified in mammals and are characterized into 4 subfamilies: class I HDACs (HDACs 1, 2, 3, 8), class II HDACs (class IIa: HDACs 4, 5, 7, 9; class IIb: HDACs 6, 10), class III HDACs (Sirt1-7) and class IV HDAC (HDAC11).^441,442^ Class I, II, and IV HDACs are Zn^2+^-dependent histone deacetylases and can be restrained by inhibitors (such as TSA and SAHA) that occupy the catalytic core of the zinc-binding site. Class III HDACs require nicotinamide adenine dinucleotide (NAD^+^) for their activity.^441–443^ Aberrant upregulation of HDACs has been reported in various types of cancers. Such changes alter the transcription of oncogenes and tumor suppressor genes, which are closely associated with cell proliferation, apoptosis, differentiation, migration, and cancer angiogenesis.^443,444^ Inhibition of HDACs has proven to be an effective strategy for cancer therapy. To date, a variety of HDAC inhibitors have been approved by the FDA (Table 4) or are undergoing clinical trials.

Based on chemical structural differences, HDAC inhibitors can be divided into four categories: hydroxamates, cyclic tetrapeptides, benzamides, and aliphatic acids.^445^ Hydroxamide HDAC inhibitors inhibit HDAC activity through coordination with Zn^2+^. TSA was the first natural hydroxamate HDAC inhibitor. Its structural analog vorinostat (SAHA) is a pan-inhibitor of classical classes of HDACs (I, II, and IV) and was approved in October 2006 for the treatment of cutaneous T-cell lymphoma (CTCL) (Table 4).^446^ Belinostat and panobinostat are two other hydroxamate pan-HDAC inhibitors that are approved for the treatment of relapsed or refractory peripheral T-cell lymphomas (PTCL) and drug-resistant MM, respectively.^447,448^ Both of them are derivatives of M-carboxycinnamic acid bishydroxamate. Cyclic tetrapeptides are a class of HDAC inhibitors with complex structures. Romidepsin isolated from *Chromobacterium violaceum* is the only such inhibitor granted US FDA approval. Its indications were initially only CTCL and expanded to PTCL in November 2011, and the objective response rate was 34% in CTCL patients and 25% in patients with PTCL.^449^ Moreover, tucidinostat (chidamide), containing pyridine and *N*-(2-amino-5-fluorophenyl)-benzamide groups, is a benzamide inhibitor of HDAC1/2/3/10. It was developed wholly in China and approved for the treatment of refractory or relapsed PTCL in 2015 by the NMPA.^450^

Aliphatic acids, such as valproic acid and phenylbutyrate, show relatively weak inhibitory activity against class I and class II HDACs, and both of these aliphatic acid HDAC inhibitors have already been approved for some non-oncological uses in the clinic and are recently under clinical evaluation for cancer therapy.^443^ Sirtuin (class III HDAC) inhibitors include the allosteric non-competitive pan-inhibitor nicotinamide and Sirt-specific inhibitors such as EX-527, sirtinol, and cambinol.^451^ Although Sirt inhibitors have been reported to be useful for cancer treatment, so far no such inhibitors have been approved for clinical use. Moreover, more than 10 other HDAC inhibitors have undergone or are undergoing clinical trials as monotherapy or in combination therapy in patients with hematologic malignancies or solid tumors.^452,453^ For example, the benzamide inhibitor entinostat is currently assessed in phase III trials (NCT02115282 and NCT03538171) for the clinical benefit in patients with HR-positive, HER2-negative, locally advanced, or metastatic breast cancer. A phase I dose-escalation multicentre trial (NCT00741234) demonstrated that the hydroxamate HDAC inhibitor pracinostat was safe, with modest single-agent activity in patients with advanced hematological malignancies.^454^

Currently, the clinical activity of HDAC inhibitors as monotherapy is largely restricted to hematological malignancies, including lymphomas, leukemia, and MM. In solid tumors, HDAC inhibitors just showed limited single-agent activity, which may be attributed to the non-specific blocking of angiogenesis and inflammation as well as the poor PK properties of some agents.^443,455^ Inhibition of tumor angiogenesis hinders drug delivery in solid tumors. The anti-inflammatory effect may induce apoptosis of tumor-fighting immune cells. Another obstacle limiting the clinical use of HDAC inhibitors is their side effects. The common side effects associated with vorinostat, belinostat, and romidepsin were nausea, anorexia, fatigue, and vomiting, which are mostly manageable, but some agents may cause more serious toxicities.^455^ Currently, major efforts to overcome these obstacles are focused on the development of small-molecule isoforms or class-selective HDAC inhibitors. Selective inhibitors can also be used as chemical probes to explore the epigenetic effects and biological processes induced by HDACs in cancer cells. Additionally, HDAC inhibitors are used in combination with other antitumor drugs to optimize their efficacy and conquer drug toxicity and resistance. Many combination therapy regimens have been used in clinical oncology treatment.^452,456,457^ Furthermore, some novel combination strategies, such as combining HDAC inhibitors with epigenetic-targeted drugs, proteasome inhibitors, or immunotherapy, have also exhibited good therapeutic effects in preclinical studies.^453,458–460^

### IDH1/2 inhibitors

Isocitrate dehydrogenases (IDHs) include three subtypes (IDH1, IDH2, and IDH3) and are key enzymes that catalyze the conversion of isocitrate to α-ketoglutarate (α-KG) using nicotinamide adenine dinucleotide phosphate (NADP^+^) or NAD^+^ as a cofactor in the tricarboxylic acid (TCA) cycle.^461^ NADP-dependent IDH1 and IDH2 are homodimeric isoenzymes with 70% sequence similarity and almost the same protein structure, whereas IDH3 is a NAD-dependent enzyme that has a unique sequence.^462,463^ IDH1/2 are mutated in several types of tumors, including AML, glioma, myelodysplastic syndrome (MDS), myeloproliferative neoplasms, chondrosarcoma, and cholangiocarcinoma.^463–467^ Mutations in IDH3 are rarely reported in cancer. IDH1 R132 H/C, IDH2 R140Q, and R172K are the main identified mutants of IDH1/2, and mutant IDH1/2 (mutIDH1/2) loses its normal catalytic function and instead catalyzes the reduction of α-KG to the oncometabolite product 2-hydroxyglutarate (2-HG).^468,469^ The accumulation of 2-HG competitively inhibits the α-KG-dependent dioxygenases involved in epigenetic remodeling and DNA repair, such as JMJD-containing histone lysine demethylases (KDMs) and the ten-eleven translocation (TET) family of 5-methylcytosine hydroxylases (DNA demethylases) (Fig. 3), thereby promoting oncogenesis through transcriptional dysregulation and impairing normal cellular differentiation.^470^ Thus, targeted inhibition of mutated IDH1/2 can reduce the serum 2-HG level and may be therapeutically beneficial for malignancies with IDH1 or IDH2 mutations.^471,472^

Two IDH1/2 inhibitors have been approved for AML therapy (Table 4). Enasidenib (AG-221) is an oral selective inhibitor of mutIDH2 with IC_50_ values of 4.0 nM on IDH2 R140Q and 340 nM on wild-type IDH2.^471^ The precursor form of enasidenib was initially screened by Agios Pharmaceuticals to find potent inhibitors of the IDH R140Q mutant, the most common form of mutIDH2 in AML, and then licensed to Celgene for further development.^472,473^ Enasidenib binds to IDH2 R140Q at an allosteric site within the heterodimer interface of the enzyme, which forces mutIDH2 to form an open conformation with no catalytic activity.^474,475^ It also showed inhibitory activity on IDH2 R172K, and its clinical efficacy was stronger in patients harboring the IDH2 R172K mutation than in patients with the IDH1 R140Q mutation (ORR: 53.3% vs. 35.4%; CR: 24.4% vs. 17.7%).^476^ In 2017, enasidenib received FDA approval for the treatment of IDH2-mutated relapsed/refractory AML.^473^ Ivosidenib (AG-120) is a reversible allosteric mutIDH1 inhibitor optimized from AGI-5198; the development of the latter has been discontinued due to its poor PKs.^477^ Ivosidenib was also developed by Agios and Celgene Pharmaceuticals and prevents the formation of catalytically active sites by binding with the cofactor (magnesium ion) of IDH1.^478^ It is highly selective for IDH1 R132 mutants but has little inhibitory activity against wild-type or mutant IDH2.^479^ The successes of preclinical studies and phase I trials earned ivosidenib orphan drug designation for glioma treatment, and it was first approved by the FDA on 20 July 2018 for adult relapsed/refractory AML patients with mutIDH1.^480^ In addition to AML indications, several clinical trials evaluating the efficacy and safety of enasidenib and ivosidenib in other tumors, such as glioma, T-cell lymphoma, MDS, or cholangiocarcinoma, are ongoing.^481,482^ Some of these studies may support the future expansion of these two drugs.

In the past few years, a variety of other IDH inhibitors have also been developed, and several of them have entered clinical trials.^483^ Typically, vorasidenib (AG-881) is an orally available pan-IDH inhibitor with IC_50_ ranges of 0.04–130 nM against various IDH1 R132, IDH2 R140, and R172 mutations. It could easily penetrate the BBB in preclinical studies, indicating the potential for glioma therapy. The drug is undergoing clinical investigation in hematologic malignancies and solid tumors including glioma. BAY-1436032 developed by Bayer is a specific allosteric inhibitor of mutIDH1, which is potent against all reported IDH R132 mutants. It could also efficaciously pass through the BBB and exert antitumor effects in glioma and AML animal models with IDH R132 mutations.^484^ Two open-label phase I trials of BAY-1436032 are ongoing to evaluate its PDs, safety, tolerability, and preliminary efficacy in patients with AML (NCT03127735) and solid tumors (NCT02746081), but the initial results have not been announced. Clinical trials of other mutIDH1 inhibitors (e.g., FT-2102, DS-1001b, and IDH305) in hematological malignancies or glioma are also underway.^485,486^ Notably, the development of IDH305 has been halted due to its dose-limiting adverse events observed in clinical studies, such as transaminitis and hyperbilirubinaemia.^487^

Although two IDH inhibitors have been approved for AML therapy and showed efficacy and safety in clinical trials of other solid tumors, including glioma, some issues still need to be resolved. Acquired resistance caused by mutIDH isoform switching was detected after treatment with specific IDH inhibitors, such as the IDH2 R140Q mutation after ivosidenib treatment and IDH1 R132C or R132H mutations after enasidenib treatment.^488^ Moreover, IDH2 secondary mutations occurring at the glutamine 316 site (Q316E mutation) or isoleucine 319 site (I319M mutation) after enasidenib treatment will change the conformation of the IDH2 trans-dimer interface and result in resistance to enasidenib.^489^ The pan-IDH inhibitor AG-881 has been reported to be effective for some mutIDH isoform switching-induced resistance.^490^ Nevertheless, there is still a demand to develop novel pan- or specific IDH inhibitors, especially mutIDH2 inhibitors, which were all previously optimized from the scaffolds of enasidenib, to conquer special-site mutations. In addition, due to the complex global epigenetic alterations in oncogenesis, IDH inhibitor monotherapy may be insufficient to treat IDH-mutated malignancies, in particular in glioma; this is also observed in clinical trials. Therefore, reasonable combination therapy with other antitumor agents, especially histone-modifying drugs or BET inhibitors, may be therapeutically beneficial for IDH-mutated patients. Interestingly, Fujiwara H et al. reported that AML patients with IDH mutations had a 14-fold higher response rate to BET inhibitors than common patients.^491^ The combination strategy of IDH inhibitors with the DNMT inhibitor azacitidine in AML treatment has also been evaluated in several clinical trials, and the preliminary results are encouraging.^492,493^

In addition to EZH2, HDAC, and IDH inhibitors, targeting DNMT is also a potential strategy for cancer therapy. Two DNMT inhibitors (azacytidine and decitabine) cxvdv have been approved for the treatment of hematologic malignancies, particularly AML and MDS.^494–496^ These cytosine analogs can incorporate into the DNA or RNA backbone to replace C-5 of cytosine with N-5 and interfere with the methylation as well as induce DNMTs degradation. Moreover, agents for other epigenetic targets are also in development. DOT1L methyltransferase inhibitor (e.g., pinometostat), BET inhibitors (e.g., birabresib, molibresib, ZEN003694, PLX51107), and lysine-specific demethylase 1 (LSD1) inhibitors (e.g., iadademstat, INCB059872, CC-90011) have progressed to different stages of clinical trials.^412,497,498^ Despite the availability of more and more epigenetic agents, there are still many problems for epigenetic therapies. Epigenetic alterations are widely distributed across normal and cancer cells. Even in the cancer cells, these changes may occur early as foundational mutations or arise late and drive clonal subgroups. Furthermore, the sensitivity to certain epigenetic regulation is discrepant in different cancers. These factors are the root causes of drug resistance and side effects. Deeper understanding of the mechanisms and difference of epigenetic alterations in different cancers may contribute to further development and optimization of epigenetic therapies. Normalization of the epigenome can sensitize cancer chemotherapy, molecular targeted therapy, and immunotherapy. Combination therapies are evaluated clinically to broaden the response rates among patients with hematologic malignancies and to expand the indication to solid tumors. However, the current results are not satisfactory. Thus far, only the combination of panobinostat, dexamethasone, and bortezomib has received FDA approval.^499^ The hope is still the deeper epigenetic insights, which may eventually guide the development of epigenetic agents and the relevant combinations.

## BCL-2 inhibitors

**Fig. 4:**
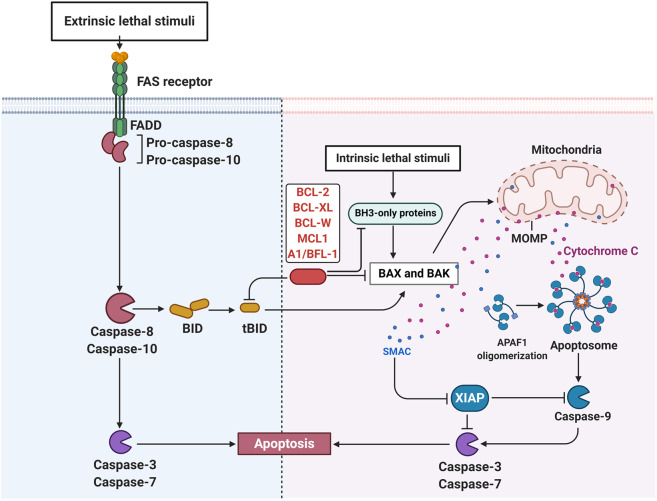
Schematic illustration of extrinsic and intrinsic pathways of apoptosis. In healthy cells, anti-apoptotic BCL-2 proteins (BCL-2, BCL-XL, BCL-W, MCL-1, and A1/BFL-1) bind to and inhibit activators (BH3-only proteins) and effectors (BAX and BAK). Treatment with BCL-2 inhibitors releases the inhibitory effects of anti-apoptotic BCL-2 proteins on activators and effectors. The subsequent activation and oligomerization of the pro-apoptotic proteins BAK and BAX result in the formation of mitochondrial outer membrane permeabilization (MOMP) and the release of cytochrome *C* as well as a second mitochondria-derived activator of caspase (SMAC) from the mitochondria. Cytochrome *C* can form a complex with procaspase 9 and apoptosis protease-activating factor 1 (APAF1), thereby activating caspase 9. Caspase 9 then activates procaspase 3 and procaspase 7, resulting in cell apoptosis. Figure created with BioRender.com

The B-cell lymphoma 2 (BCL-2) family of proteins consists of more than 20 members that regulate the intrinsic apoptosis pathway, and fall into three subfamilies (anti-apoptotic proteins, pro-apoptotic proteins, and the cell death mediators) based on their structure and function.^500^ Anti-apoptotic proteins such as BCL-2 and the closely related proteins BCL-XL, BCL-W, MCL-1, and A1/BFL-1 have four tandem BCL-2 homology (BH) domains and promote cell survival.^500,501^ While the multi-region pro-apoptotic effector proteins (BAX and BAK) and BCL-2 homology 3 (BH3)-only pro-apoptotic proteins (BIM, PUMA, BAD, or NOXA) promote cell apoptosis upon diverse cellular stresses by the alternation of mitochondrial outer membrane permeabilization and release of cytochrome *C* from the mitochondria (Fig. 4).^501,502^ The interaction between the anti-apoptotic and pro-apoptotic BCL-2 family of proteins regulates the apoptotic state of cells. Dysregulation of the apoptosis pathway is common in malignant tumors, especially in hematologic malignancies.^503–505^ BCL-2 was first discovered as an inhibitor of cellular apoptosis, and the in-depth understanding of this target promotes the development and application of BCL-2 inhibitors in cancer treatment.^506,507^

Several early efforts have been made to target the anti-apoptotic BCL-2 family members including antisense oligonucleotide drug oblimersen, the natural product gossypol, and its synthetic derivatives.^508^ A major breakthrough in targeting BCL-2 is the development of small-molecule BH3 mimetics ABT-737 and ABT-263 (navitoclax).^509,510^ These agents can mimic the physiologic inhibitors of anti-apoptotic BCL-2 and its relatives, thereby promoting apoptosis. ABT-737 is the first synthetic BH3 mimetic discovered by structure-activity relationship and nuclear magnetic resonance screening. It binds to BCL-2, BCL-XL, and BCL-W with high affinity, and exhibits 500-fold weaker affinity for MCL1 and BFL-1/A1.^509^ However, ABT-737 has poor bioavailability and requires continuous parenteral administration, which hindered its clinical development. Navitoclax is a structurally related molecule of ABT-737 with high oral bioavailability (~50% in dogs) and entered clinical trials in 2006.^511^ Although the efficacy of navitoclax was observed in preclinical and clinical studies, dose-dependent thrombocytopenia caused by BCL-XL suppression is the major obstacle restricting the clinical application of navitoclax.^510^ Initial efforts on the BH3 mimetics ABT-737 and navitoclax facilitated the successful development of ABT-199 (venetoclax), a highly selective BH3 mimetic with greater affinity for BCL-2 but a much lower affinity for BCL-XL and BCL-W.^512^ In a single-arm phase II trial of venetoclax monotherapy, 79.4% of patients with CLL with the 17p deletion achieved an objective response over a median of 12 months among 107 enrolled patients, and complete remission was achieved in a median of 8.2 months (NCT01889186).^513^ Due to its remarkable efficacy and safety, the FDA-approved venetoclax for the treatment of CLL patients with 17p deletion in April 2016 (Table 5). This indication was subsequently expanded in June 2018 to include patients with CLL or SLL, with or without 17p deletion, who have received at least one prior therapy. Moreover, in November 2018, venetoclax was approved in combination with standard chemotherapy agents, including azacitidine, decitabine, and low-dose cytarabine, for the treatment of newly diagnosed AML in adults who are 75 years of age or older, or who have comorbidities that preclude the use of intensive induction chemotherapy; this approval was based on the impressive results of two clinical studies conducted in this population.^514,515^ As a BCL-2-specific inhibitor, venetoclax is also under assessment for the treatment of many other malignancies in the clinic, including solid tumors, and most of the clinical trials have tested its role in combination therapies.^516^

**Table 5:** Properties of approved small-molecule inhibitors of BCL-2, hedgehog pathway, proteasome, and PARP

Chemical structure	Name	Targets	Approved indications (year)	Corporation
	Venetoclax (Venclyxto)	BCL-2	CLL (2016) SLL (2018) AML (2018)	Abbvie/Genentech
	Vismodegib (Erivedge)	SMO	BCC (2012)	Genentech
	Sonidegib (Odomzo)	SMO	BCC (2015)	Novartis
	Glasdegib (Daurismo)	SMO	AML (2018)	Pfizer
	Bortezomib (Velcade)	Proteasome	MM (2003) MCL (2006)	Millennium
	Carfilzomib (Kyprolis)	Proteasome	MM (2012)	Onyx
	Ixazomib (Ninlaro)	Proteasome	MM (2015)	Takeda
	Olaparib (Lynparza)	PARP1/2/3	Ovarian cancer (2014) FTC (2015) Breast cancer (2018) Pancreatic adenocarcinoma (2019) Prostate cancer (2020)	AstraZeneca
	Rucaparib (Rubraca)	PARP1/2/3	Ovarian cancer (2016) FTC (2016) PPC (2018) Prostate cancer (2020)	Clovis
	Niraparib (Zejula)	PARP1/2	Ovarian cancer (2017) FTC (2017) PPC (2017)	Tesaro/Takeda/Janssen
	Talazoparib (Talzenna)	PARP1/2	Breast cancer (2018)	Pfizer

S55746 (also known as BCL201 or Servier-1) is another orally bioavailable small-molecule BCL-2 inhibitor being studied in clinical trials. It is a BH3 mimetic derived from tetrahydroisoquinoline amide-substituted phenyl pyrazoles, and shows high affinity and selectivity for BCL-2 with a Ki value of 1.3 nM.^517^ The results of a phase I study (NCT02920697) indicated the efficacy and safety of S55746 in patients with B-cell NHL. In addition to the BCL-2 inhibitor, several inhibitors targeting MCL-1 or BCL-XL are also being evaluated in clinical trials. AMG-176 was the first selective MCL-1 inhibitor to be evaluated in the clinic, and is currently being assessed in a phase I study for efficacy, tolerability, and PK properties in patients with relapsed/refractory MM or AML (NCT02675452).^518^ S64315 (MIK665), another MCL-1 inhibitor derived from S63845, can induce apoptosis in diverse hematological malignancies in a Bax/Bak-dependent manner.^519^ It is undergoing evaluation in several phase I clinical trials in patients with relapsed/refractory AML or MDS (NCT02979366), and relapsed/refractory MM or lymphoma (NCT02992483). Two other MCL-1 inhibitors AZD-5991^520^ and AMG-176^521^ have also entered clinical trials to evaluate their safety, PKs, and antitumor response in patients with hematological malignancies (NCT03218683 and NCT02675452). Neither efficacy nor safety data of MCL-1 inhibitors are currently available. In addition, since BCL-XL overexpression is closely related to the oncogenesis of various solid tumors, targeting BCL-XL is a promising therapeutic strategy for some malignancies, although thrombocytopenia induced by BCL-XL inhibition remains a concern. Multiple BCL-XL inhibitors such as WEHI-539 and A-1331852 have been developed, and exhibit significant monotherapy activity in preclinical studies carried out in solid tumors.^522^ As the time of this review, none of the selective BCL-XL inhibitors have entered clinical trials.

The success of BCL-2 family inhibitors in the treatment of hematological malignancies has broken new ground in small-molecule targeted therapy by confirming that protein–protein interactions can also be efficiently and specifically targeted. Despite favorable outcomes, challenges for these inhibitors remain, including identifying reliable biomarkers of response and elucidating resistant mechanisms to BCL-2 inhibition. A study conducted using samples from patients with venetoclax-treated CLL found that neither a cell sensitivity test in vitro nor TP53 status was a predictor of the antitumor response to venetoclax.^523^ BH3 profiling, an in vitro assay to determine the dependence of tumor on individual anti-apoptotic protein, was reported as a potential solution to this problem.^523^ However, due to the diversity of factors influencing the efficacy of BCL-2 inhibitors, the reliability of this finding still requires validation by systematic clinical evaluation. As has been demonstrated repeatedly, cancer cells eventually become resistant to targeted therapies, and BCL-2 apoptotic pathway-dependent malignancies are no exception. The resistance mechanisms involve upregulation of alternative anti-apoptotic BCL-2 family members such as BCL-XL and MCL-1, mutation or phosphorylation of BCL-2, and loss-of-function mutations of pro-apoptotic proteins BAX and BAK.^524^ At present, combination therapy either with diverse anti-apoptotic BCL-2 family inhibitors or with BCL-2 family inhibitors and conventional chemotherapy is the main strategy to overcome resistance. There are considerable clinical studies focusing on these combined effects.^524,525^ How to choose synergistic drugs and avoid the possible side effects are new challenges for researchers. As data from multiple phase I trials of combination therapy become available, close and critical assessments are needed.

## Hedgehog pathway inhibitors

**Fig. 5:**
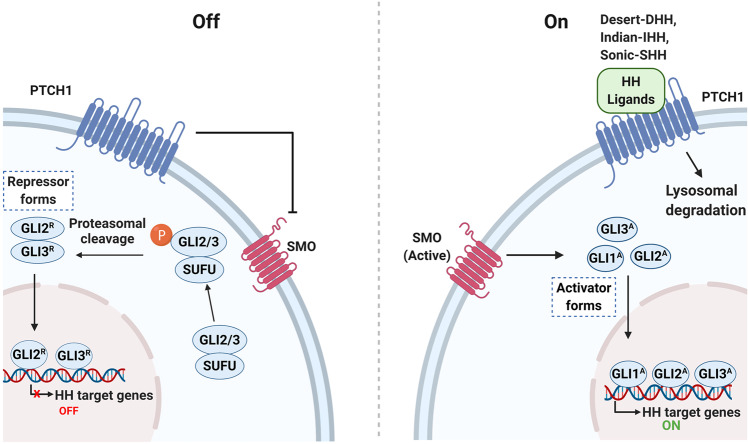
Canonical SMO-dependent hedgehog (HH) signaling pathway. Unliganded PTCH1 prevents the ciliary translocation of SMO effector protein. GLI2 and GLI3 proteins are sequestered in the cytoplasm by SUFU and phosphorylated by protein kinases, thereby preventing HH target-gene transcription. HH ligands binding triggers endocytic internalization of PTCH1, which results in the accumulation and activation of SMO. Active SMO relieves SUFU-mediated inhibition of GLI2 and GLI3. Activator forms of GLI (GLI1^A^/GLI2^A^/GLI3^A^) translocate into the nucleus and initiate the transcription of target genes. Figure created with BioRender.com

The hedgehog (HH) signaling pathway is highly conserved and has an important role in embryonic development and tissue regeneration. The HH pathway can be divided into canonical and noncanonical pathways. Activation of the canonical HH pathway is initiated by the release of HH ligands (Desert-DHH, Indian-IHH, and Sonic-SHH), and these ligands can bind and suppress the 12-pass transmembrane receptor Patched-1 (PTCH1).^526^ In the absence of HH ligands, PTCH1 constitutively inhibits HH signaling by suppressing the transmembrane transducer Smoothened (SMO). Upon ligand binding, PTCH1 is internalized from the cell membrane and degraded, which results in the release of SMO to the primary cilium and phosphorylation at the cytoplasmic end. Active SMO promotes the activation of the glioma-associated oncogene (GLI) transcription factors GLI1, GLI2, and GLI3 and then induces the expression of target genes related to cell proliferation, survival, and differentiation (Fig. 5).^526^ HH signaling can be abnormally activated through various mechanisms, such as HH ligand upregulation and PTCH1 or SMO mutations. Accumulating evidence has suggested that aberrant activation of the HH pathway is closely related to the oncogenesis and progression of a variety of tumors, including solid carcinomas and hematological tumors, as well as self-renewal of cancer stem cells (CSCs).^527,528^ Therefore, the HH pathway has emerged as an attractive target for cancer therapy.

To date, three HH pathway inhibitors have been approved by the FDA for clinical oncology treatment (Table 5). Among them, both vismodegib and sonidegib are oral SMO inhibitors and are used for the treatment of locally advanced, unresectable, or metastatic basal cell carcinoma (mBCC).^529,530^ The results of an open-label trial (NCT01327053) for vismodegib showed a response rate of 68.5% in 1119 locally advanced BCCs (laBCCs) and 36.9% in 96 mBCC patients. The median PFS in laBCC and mBCC was 13.1 and 23.2 months, respectively.^531^ Moreover, in a randomized trial of sonidegib for 66 laBCCs and 13 mBCCs (NCT00833417), the 200 mg/day treatment group had response rates of 57.6% and 7.7%, respectively. The disease control rate, including stable disease, was 91.9% in laBCC and 92.3% in mBCC.^532^ In addition to BCC, many clinical trials have been performed to evaluate the efficacy of these two agents in the treatment of various tumors, including rare tumors.^533–536^ Glasdegib is also a selective HH pathway inhibitor that binds to SMO. In 2018, it was approved by the FDA in combination with low-dose cytarabine chemotherapy for newly diagnosed AML patients who were older than 75 years or unable to receive intensive chemotherapy due to chronic health problems and diseases.^537^ This is also the first HH pathway inhibitor approved to treat AML.

Several other SMO inhibitors have also entered clinical trials to evaluate their therapeutic effects on BCC, pancreatic cancer, colon cancer, and breast cancer.^538^ However, clinical studies of some drugs, such as saridegib (a cyclopamine analog), TAK-441, IPI-926, and CUR-61414, have been terminated due to detrimental effects and lack of response.^539^ It is worth mentioning that the FDA-approved antifungal drug itraconazole was found to have an inhibitory effect against the HH pathway by antagonizing SMO. It has been reported that the clinical benefit of high-dose itraconazole in prostate cancer was mainly attributed to HH signaling inhibition rather than an anti-androgen effect.^540^ Moreover, vitamin D3 could also bind SMO with high affinity and is currently in phase I or phase III clinical trials as a neoadjuvant for the treatment of BCC, pancreatic cancer, CLL, and NHL.^539^

Inhibition of GLI-mediated transcription is an alternative strategy for developing HH signaling inhibitors, and this strategy has the potential to overcome acquired resistance of the approved SMO inhibitors. Currently, there are many reports on GLI inhibitors, but most of them are in the preclinical stage. Arsenic trioxide (ATO), a well-known agent approved by the FDA for acute promyelocytic leukemia treatment, is also a GLI inhibitor. Mechanistically, ATO blocks GLI2 accumulation and thus inhibits the transcriptional activation of GLI target genes. In a preclinical study, treatment with ATO or its combination with itraconazole effectively inhibited the growth of medulloblastoma harboring the SMO D477G mutation (or SMO D473H in humans) that is resistant to SMO inhibitors.^541^ In a clinical study conducted in patients with refractory mBCC, combined treatment with ATO and itraconazole induced stable disease in 3 of 4 patients.^542^ The hexahydropyrimidine derivatives GANT58 and GANT61 screened by constructed cells are selective GLI inhibitors.^543^ They could interfere with GLI1/2-mediated transcriptional activity by blocking their DNA binding and restraining the growth of multiple tumors in a GLI-dependent manner. However, there has been no clinical study of these drugs due to their restricted pharmacological applicability.

Although the approved HH pathway inhibitors (SMO inhibitors) have shown significant antineoplastic activity in a variety of tumors, especially BCC, the diseases relapse and progress within several months due to acquired resistance. The resistance mechanisms involve SMO mutations, such as SMO D473H, activation of HH signaling downstream of SMO, and activation of compensatory signaling pathways.^544^ As mentioned above, GLI inhibitors have the potential to conquer resistance to SMO inhibitors. Moreover, novel therapeutics targeting HH signaling has also been reported. LEQ-506 is an SMO inhibitor undergoing phase I clinical studies in some advanced solid tumors (NCT01106508). It could suppress the proliferation of medulloblastoma cells harboring the SMO D473H mutation, which is derived from a patient who relapsed after an initial response to vismodegib treatment.^545^ The SMO inhibitor taladegib can bind to and inhibit SMO D473H activity. A phase I trial (NCT01919398) assessed the efficacy of taladegib in several advanced solid tumors and suggested that it is effective only in BCC with an estimated ORR of 46.8%.^546^ This clinical trial also indicated that taladegib could benefit patients who were resistant to SMO inhibitors. As the compensatory upregulation of RAS/MAPK and PI3K-mTOR signaling pathways contributes to the mechanism of SMO-independent GLI regulation and resistance to SMO inhibitors, the combined use of HH signaling inhibitors and PI3K or MAPK pathway inhibitors may be a potential strategy to overcome the current resistance to SMO inhibitors.^528,547^

## Proteasome inhibitors

**Fig. 6:**
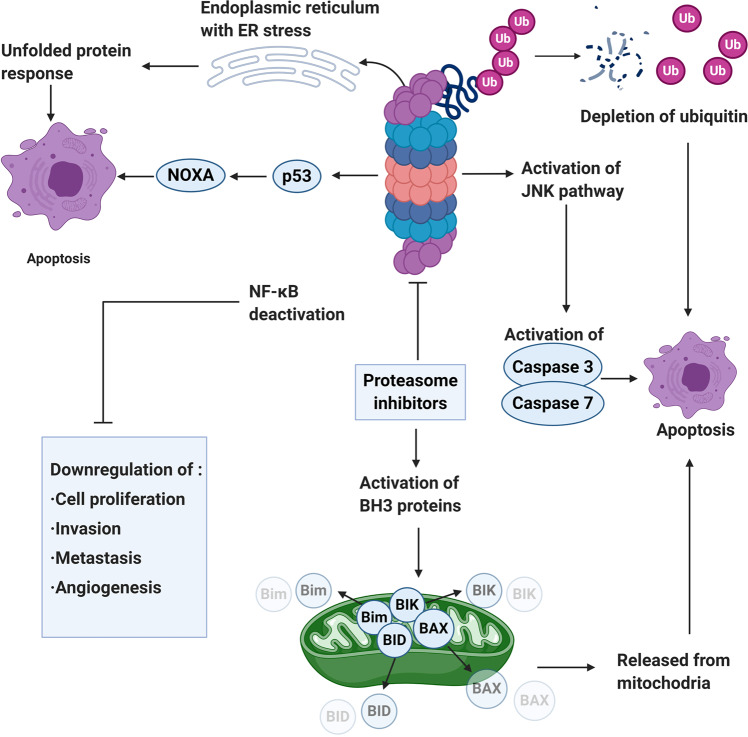
Proteasome inhibition acts through multiple mechanisms to induce cell apoptosis. Proteasome inhibition leads to NF-κB deactivation, thereby downregulating multiple pro-neoplastic pathways associated with cell proliferation, invasion, metastasis, and angiogenesis. Inhibition of proteasome activates the JNK signaling pathway and results in programmed cell death via caspase 3 and 7. Additionally, proteasome inhibition can indirectly cause apoptosis by preventing the degradation of pro-apoptotic family proteins such as BAX, BID, BIK, and BIM as well as NOXA. Inhibition of proteasome prevents the degradation of ubiquitinated proteins, which can increase endoplasmic reticulum (ER) stress and activate the UPR, cell cycle arrest, and subsequent apoptosis. Figure created with BioRender.com

Proteasomes are large multicatalytic enzyme complexes that are expressed in the nucleus and cytoplasm of all eukaryotic cells and are responsible for more than 80% protein degradation in human cells.^548^ The ubiquitin–proteasome system (UPS) has an important role in maintaining cellular protein homeostasis and regulating numerous biological processes, such as cell survival, signal transduction, DNA repair, and antigen presentation.^549^ Most misfolded, unassembled, or damaged proteins that could otherwise form potentially toxic aggregates are degraded via UPS, in which proteins are tagged by ubiquitin and then recognized and degraded into small peptides by the proteasome complex (Fig. 6). Structurally, all proteasomes contain a common core, referred to as the 20S proteasome. The 20S core consists of a cylinder made of four stacked rings: 2 identical outer α-rings and 2 identical inner β-rings, each containing 7 distinct but related subunits.^548,550^ The specificity of the 20S proteasome for substrate action depends on the peptide bond on the N2 terminal threonine residue of the β1, β2, β5 subunits. Dysfunction of the UPS is related to multiple human diseases, such as cancers, autoimmune diseases, and genetic diseases;^550,551^ thus, much work has been conducted by targeting the UPS as a potential treatment strategy.

Multiple myeloma (MM) cells produce excessive paraproteins, and their growth is dependent on proteasome-regulated signaling pathways. Therefore, MM cells are particularly susceptible to proteasome inhibition, and proteasome inhibitors (PIs) have become the backbone of MM clinical therapy.^552^ Bortezomib is the first approved PI for the treatment of relapsed or refractory MM (Table 5). It is a peptide boronic acid and reversibly acts on the β5 catalytic subunit of the proteasome.^553^ The clinical application of bortezomib significantly improves the long-term outcomes for MM patients. Moreover, Bortezomib has also been approved for the treatment of MCL. Currently, there are more than 200 clinical trials related to bortezomib that focus on its combination with other agents, efficacy in other cancers, and even other noncancer applications such as graft-versus-host disease.^554^ However, bortezomib treatment has several limitations including primary resistance in MM and MCL patients, relapse in many initially-responding patients, and induction of dose-limiting peripheral neuropathy (PN).^554,555^ To conquer these limitations, the second-generation PIs have been developed, and many of them are derived from synthetic and natural products.^552,556^ Carfilzomib approved by the FDA in 2012 is a second-generation PI and is derived from the natural product epoxomicin (Table 5).^557^ Unlike bortezomib, carfilzomib is an irreversible inhibitor that contains an epoxyketone warhead, which could covalently bind to the N-terminal threonine-containing active sites of the 20S proteasome.^558^ The irreversible nature of carfilzomib contributes to its efficacy even in MM patients relapsed from or refractory to bortezomib. In addition, the carfilzomib-containing regimens exhibit significantly reduced peripheral neurotoxicity, while cardiovascular events were observed in MM patients treated with carfilzomib.^557^ Another second-generation PI ixazomib was approved in 2015 in combination with lenalidomide and dexamethasone for MM patients who have received at least one prior therapy (Table 5).^559^ Ixazomib is an N-capped dipeptidyl leucine boronic acid that reversibly inhibits the CT-L proteolytic (β5) site of the 20S proteasome.^560^ Ixazomib shares the same pharmacophore boronic acid residue with bortezomib, however, the elimination half-life of the former is much shorter than that of the latter (18 vs. 110 min), which may contribute to the improved safety profiles of xazomib over bortezomib.^559,560^ Patients resistant to bortezomib can still benefit from ixazomib treatment. Moreover, it is worth mentioning that both bortezomib and carfilzomib require parenteral (intravenous or subcutaneous) administration, whereas ixazomib is the first oral PI and is a prodrug.^561^ Ixazomib is rapidly hydrolyzed to the active form (MLN2238) under physiological conditions.

Following the clinical success of the PIs, a number of novel PIs have been developed in recent years but only three of them are currently being evaluated in clinical trials. Marizomib (Salinosporamide A) is a naturally occurring β-lactone-γ-lactam bicyclic compound isolated from Salinispora bacteria. It can bind to 3 major catalytic sites on β5, β1, and β2 of proteasome irreversibly and inhibit proteasome activity at nanomolar concentrations.^562^ The efficacy of marizomib in relapse or refractory MM and lymphoma is evaluated in phase I/II trials (NCT00461045, NCT00396864). Moreover, marizomib is reported to have CNS adverse events, which suggests that it can penetrate the BBB.^563^ Therefore, it is currently under evaluation in a phase III clinical trial for malignant glioblastoma treatment (NCT03345095). Oprozomib is a structural analog of carfilzomib, with a peptide-like backbone and an epoxyketone warhead.^564^ The development of oprozomib aims to find an oral PI by chemical modification of carfilzomib. Currently, it is assessed in clinical trials for the treatment of refractory MM or relapsed MM after receiving bortezomib- or carfilzomib-based therapies (NCT02939183). Another reversible PI under clinical assessment is delanzomib, an orally bioavailable structural homolog of bortezomib with a peptide-like backbone and a boronate warhead.^565^ It is assessed in several phase I/II clinical trials for MM, lymphoma, and solid tumors.^566,567^ Compared with BTZ, delanzomib showed slightly reduced efficacy against MM and some solid tumor cells, but had higher selectivity to cancer cells over normal cells, which means better drug safety. A phase I study of delanzomib also exhibits that it has a favorable safety profile with limited PN.^566^

While emerging as an important treatment strategy for MM, PIs showed weak efficacy against solid cancers. The lack of clinical benefits of PIs in treating solid cancers was speculated to be attributed to their short elimination time and poor distribution to the proteasome target located in solid cancers.^554,568^ Moreover, drug resistance has also been a major hurdle for PIs. As mentioned above, the next-generation PIs with improved PK/PD characteristics may promote the clinical efficacy of MM patients as well as those who are resistant to existing PI treatment, and extend treatment benefits to solid cancers. Meanwhile, combined proteasome inhibition with other active agents, such as BCL-2 inhibitor, HDAC inhibitor, immunomodulatory agents, and some other inhibitors of signaling pathways related to resistant mechanisms, is also a strategy to conquer resistance to PIs.^569^ These combination therapies are being assessed in many preclinical and clinical studies.

## PARP inhibitors

**Fig. 7:**
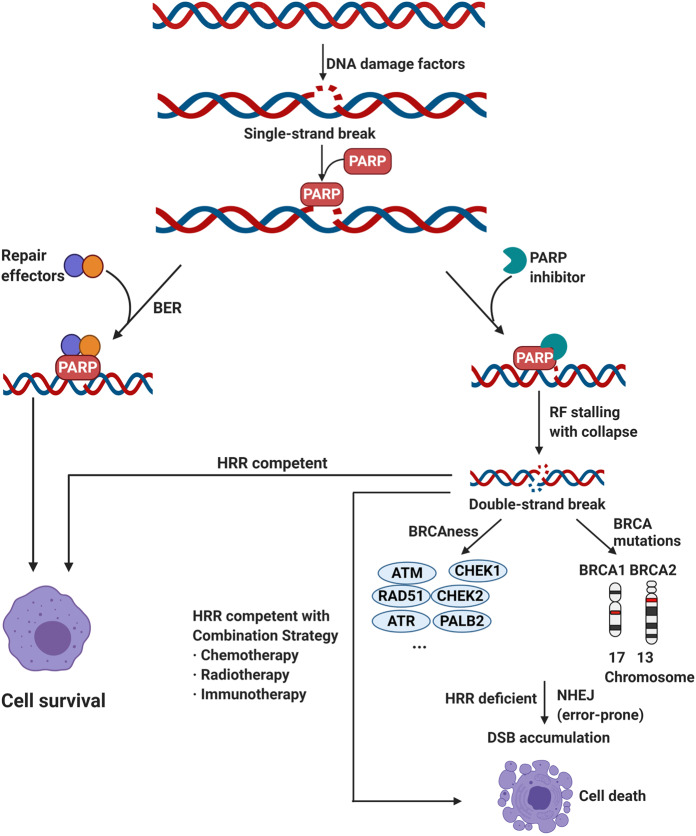
Molecular process of DNA damage repair related to PARP and the mechanism of action of PARP inhibitors. Endogenous single-strand breaks (SSB) are repaired mostly by PARP-dependent base excision repair (BER) pathway. PARP inhibitors suppress the repair of SSB and the unrepaired SSB can be converted to double-strand breaks (DSB) that are toxic to cells. Homologous recombination (HR) is the major pathway to repair DSB. However, the DSB in BRCA1/2 mutant cells cannot be repaired through HR, thus resulting in genomic instability and cell death. Figure created with BioRender.com

Genomic instability is one of the typical characteristics of tumor cells. To maintain genomic integrity, tumor cells have multiple mechanisms to repair DNA lesions, such as the repair pathways of DNA double-strand breaks (DSBs) and single-strand breaks (SSBs). Among them, the former includes homologous recombination and non-homologous end joining (NHEJ), while the latter includes base excision repair (BER), nucleotide excision repair (NER), and mismatch repair (MMR).^570^ Poly (ADP-ribose) polymerases (PARPs) are a group of multifunctional post-translational modification enzymes that engage in a diverse set of cellular processes, including DNA repair, transcription, mitosis, and cell cycle regulation.^571^ To date, 18 members have been identified in PARP family proteins, among which PARP1 is the best-studied PARP member and has an important role in the repair of DNA SSBs. Once DNA SSBs occur, PARP1 binds to damaged DNA through N-terminal zinc finger domains, allowing its cofactor β-nicotinamide adenine dinucleotide (β-NAD) to bind to the active site of the enzyme and activating the catalytic function of the ADP-ribosyltransferase catalytic domain. PARP1 then catalyzes the transfer of PAR chains to the target proteins (PARylation) in the vicinity of the DNA breaks, which promotes chromatin remodeling and the recruitment of a series of DNA repair effectors and completes the DNA repair process (Fig. 7).^570,571^ PARP1 autoPARylation eventually causes its dissociation from DNA damage and restores its autoinhibitory status. Inhibition of another DNA repair pathway in cancer cells with defective DNA repair mechanisms may create a “synergistic lethal” effect; this theory was first proposed by Theodosius Dobzhansky et al.^572^ Breast cancer susceptibility genes *BRCA1* and *BRCA2* are two key tumor suppressors that repair DNA DSBs. Mutations in *BRCA1* and *BRCA2* are susceptible to breast and ovarian cancers, and DSBs are not easily repaired in *BRCA*-mutant tumor cells.^573^ Therefore, PARP inhibition in *BRCA-*mutant cancers can induce synthetic lethality due to the simultaneous blockade of both DSB and SSB repair pathways (Fig. 7).

Nicotinamide, the cofactor of PARPs, which competes with NAD for the catalytic pocket of PARPs, is the first identified PARP inhibitor.^574^ Currently, four PARP inhibitors with nicotinamide pharmacophores (olaparib, rucaparib, niraparib, and talazoparib) have been approved by the FDA or the EMA (Table 5).^575–578^ All PARP inhibitors have the ability to inhibit the catalytic activity of PARPs. However, this mechanism cannot fully explain the antitumor activity of PARP inhibitors. They can also trap PARP in a non-effective state at chromatin, and such binding to the PARP–chromatin complex will produce more effective cytotoxicity.^579^ Based on in-depth research on the mechanisms of action of PARP inhibitors and the results of clinical trials, indications of PARP inhibitors have been continuously updated since the first inhibitor olaparib was approved in 2014. Olaparib was originally approved for patients with deleterious or suspected deleterious germline *BRCA*-mutant advanced ovarian cancer who had undergone three or more prior lines of chemotherapy, followed by rucaparib in 2016, and niraparib in 2017.^575,576^ Later, in 2017 and 2018, olaparib, niraparib, and rucaparib were approved as maintenance therapies in recurrent platinum-sensitive cancers, including epithelial ovarian cancer, fallopian tube cancer (FTC), or primary peritoneal cancer (PPC), regardless of *BRCA* status.^575–577^ Furthermore, olaparib became a first-line maintenance treatment in germline or somatic *BRCA*-mutated cancer patients who responded to platinum-based chemotherapy according to the data of clinical trial (NCT01874353).^580^ In 2018, the FDA successively approved olaparib and talazoparib for *BRCA*-mutated HER2-negative locally advanced or metastatic breast cancer. In addition, niraparib expanded the indications for the treatment of advanced ovarian cancer, FTC, and PPC associated with homologous recombination deficiency positive status in 2019.^581^

At present, a large number of clinical trials related to olaparib, rucaparib, niraparib, or talazoparib alone or in combination are still underway to identify responding patients beyond the ovarian or breast cancer population. Additionally, several other PARP inhibitors, such as pamiparib, veliparib, INO-1001, E7449, iniparib, AZD2461, amelparib, IMP4297, RBN-2397, and fluzoparib, have also entered clinical trials and are in different stages.^582,583^ Among them, veliparib (ABT-888) is an orally bioavailable PARP inhibitor developed by Abbvie that can cross the blood–brain barrier.^584^ Its clinical efficacy has been evaluated in multiple solid tumors. A phase III trial of veliparib with first-line chemotherapy showed an increased PFS in high-grade serous ovarian carcinoma compared with paclitaxel and carboplatin alone (NCT02470585). However, the clinical outcomes could not be significantly improved by veliparib as monotherapy in patients with ovarian or pancreatic cancer as well as by its combination with carboplatin/paclitaxel in TNBC.^585^ To date, veliparib has not been approved by any agency.

The efficacy of PARP inhibitors in cancer treatment is not restricted to *BRCA1/2* mutant sufferers. In the treatment of ovarian cancers, 40–70% of *BRCA1/2* mutated patients failed to respond to PARP inhibitors.^586^ Platinum sensitivity is also reported as a prospective indicator for predicting the response to these inhibitors.^587^ Nevertheless, these two predictors still cannot completely cover the tumor types suitable for PARP inhibitor treatment. This is one of the problems that need further research in PARP inhibition therapy. In addition, with the widespread use of PARP inhibitors in the clinic, the issue of acquired resistance has emerged frequently. Mechanisms of resistance are multifactorial, mainly involving the restoration of HR function caused by secondary mutations of homologous recombination repair genes (*BRCA1/2*, *RAD51C/D*, *PALB2*) and defects in NHEJ as well as loss of 53BP1, stabilization of replication forks, PARP1 mutations, and drug efflux.^588^ Combined therapy is increasingly explored as a means to enhance the efficacy and overcome drug resistance to PARP inhibitors. Many studies have shown that simultaneously targeting PARPs and cell cycle checkpoints (ATR, CHK1, and WEE1) can overcome the resistance caused by restored replication fork stabilization.^589,590^ A phase II study of olaparib plus ATR/WEE1 inhibitor vs. olaparib monotherapy is currently underway in TNBC patients (NCT04191135).^591^ Moreover, it should be noted that combination therapy may also increase the risk of side effects. Severe myelosuppression was observed in several phase I trials of PARP inhibitors combined with chemotherapy drugs.^592^ It is still of great importance to better understand the predictors and resistance mechanisms of PARP inhibitors and ultimately improve diagnosis, treatment strategy, and outcome.

## Concluding remarks: challenges and future perspectives

With the evolution of modern molecular biology and the application of some advanced technologies such as computer-aided drug design, structure biology, and combinatorial chemistry, small-molecule targeted anti-cancer drugs have entered a rapid development stage. To date, 89 small-molecule targeted drugs have been approved by the FDA and/or NMPA to treat various cancers (Fig. 1). Thousands of targeted agents are undergoing clinical trials for cancer treatment (Supplementary Fig. S1). Among them, a large number of promising agents have advanced to phase III trials (Supplementary Tables S2–S5). According to the prediction of the Business Research Company, the global anti-cancer drug market size will reach 200 billion dollars in 2021, among which targeted drugs are the “main force”. Despite the significant progress achieved, there are still some challenges that small-molecule targeted anti-cancer drugs face.

**Fig. 8:**
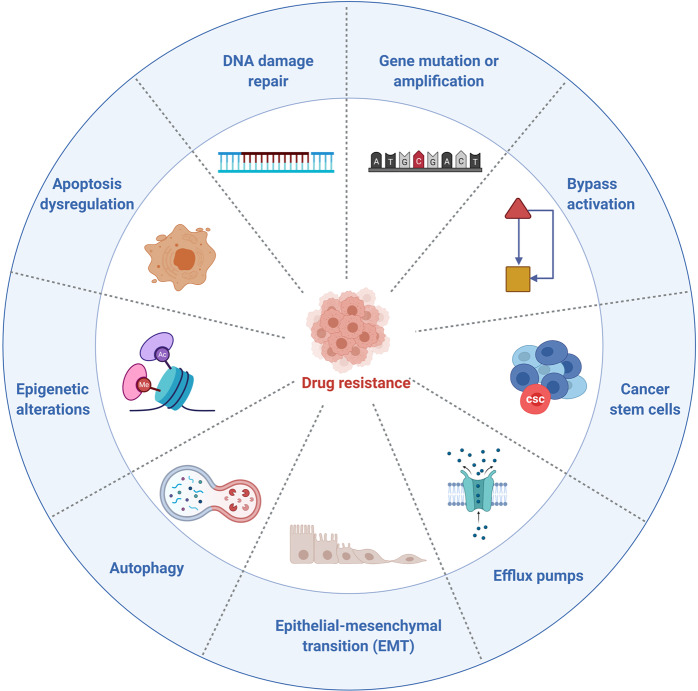
Mechanisms and insights in drug resistance of small-molecule targeted anti-cancer agents. Figure created with BioRender.com

The first major challenge is drug resistance. Almost all targeted anti-cancer drugs come across resistance after a period of time of clinical use. Drug resistance has been linked to many mechanisms, including gene mutation, amplification, CSCs, efflux transporters, apoptosis dysregulation, and autophagy, etc (Fig. 8).^593–599^ Gene mutation is the main reason leading to anti-cancer drug resistance. There are two different views regarding drug-resistant gene mutations. One is that the gene mutations are induced by drugs. The other one is that the drug-resistant mutations have already existed. In the early stage of treatment, cancer cells with drug-sensitive mutations dominate and suppress the proliferation of cells containing drug-resistant mutations. After cells with sensitive mutations are killed, the resistant mutant cells become the mainstream and show resistance. Amplification of other genes is another common reason for anti-cancer drug resistance. For example, *MET* amplification accounts for about 20% of EGFR inhibitor-resistant cases.^46^ CSCs are also thought to be an important reason for drug resistance and recurrence.^595,596^ The CSC theory proposes that the different cells within a tumor, as well as metastasis deriving from it, originate from a single subpopulation of cells with self-renewal and differentiation capacities, which are similar to stem cells. Overexpression of efflux transporters, such as multidrug resistance transporter proteins, especially P-glycoprotein, which renders the resistance to chemotherapeutic drugs, also has a role in the targeted anti-cancer drug resistance.^597,598^ In addition to these causes, apoptosis dysregulation and autophagy also could be responsible for anti-cancer drug resistance.^593,599^

Low efficiency is another major challenge for targeted anti-cancer drugs. As mentioned many times before, targeted anti-cancer drugs are effective only in a limited number of patients. For example, less than 20% of patients with NSCLC are sensitive to EGFR inhibitors (e.g., gefitinib and erlotinib). Patients that are sensitive to these EGFR inhibitors are found to harbor EGFR-activating mutations (for example, exon 19 deletion or exon 21 L858R point mutations).^74^ TRK inhibitors larotrectinib and entrectinib have been approved for the treatment of patients with *NTRK* gene rearrangements, regardless of cancer type and patient age. These rearrangements can be found at high frequencies (up to 90%) in certain types of cancer, such as infantile fibrosarcoma (a rare disease), but the incidence is only 1% of all malignancies.^225^ These highlight the importance of the identification of predictive biomarkers for response to targeted anti-cancer drugs.

Currently, in order to deal with the major challenges of targeted anti-cancer drugs, many strategies have been applied, such as new generation anti-cancer drugs against drug resistance mutations, multitarget drugs, combination therapy, and drugs targeting CSCs.^594,600,601^ In addition to these, several new research trends in this area deserve attention.

The first one is the drug discovery against new type cancer targets. For example, in the past years, some new epigenetic regulatory proteins have garnered increasing attention, such as RNA m6A methylation-related proteins (METTL3/14, FTO, ALKBH5, WTAP, and YTHDFs).^602,603^ microRNAs (miRNAs) are another new type of cancer targets, which are frequently dysregulated in cancers and may serve as promising targets for cancer therapy. Currently, some effects have been paid to discover small-molecule inhibitors against miRNAs, such as miR-21 inhibitor AC1MMYR2 (also known as NSC211332), Lin28-let-7 inhibitors 6-hydroxy-DL-DOPA, SB/ZW/0065, and KCB3602.^604^

Furthermore, some proteins that are previously thought undruggable may also be attractive anti-cancer targets. A typical example is *KRAS*, a most frequently mutated isoform of *RAS* proto-oncogene, which has a predominant role in driving the initiation and progression of cancers.^605,606^ Decades of efforts failed to target KRAS with small molecules due to the high binding affinity of the intrinsic ligand GTP to KRAS, the relatively small size and smooth surface of KRAS, and the high flexibility of KRAS switch regions.^607–609^ However, this dilemma has begun to change recently. A variety of novel small molecules that directly target KRAS are being developed, including covalent allosteric inhibitors for KRAS G12C mutant, protein–protein interaction inhibitors that bind in the switch I/II pocket or the A59 site, and GTP-competitive inhibitors targeting the nucleotide-binding site.^609,610^ To date, four KRAS G12C covalent inhibitors (AMG510, JNJ-74699157, MRTX849, and GDC-6036) with similar allosteric mechanisms have advanced to clinical trials.^611,612^ In addition to KRAS, other types of such previously undruggable cancer targets include MYC,^613^ phosphatases,^614^ and protein–protein interactions,^615,616^ etc.

The second one is the combination of small-molecule targeted drugs with immunotherapy such as PD-1 antibody. Lenvatinib plus pembrolizumab, a PD-1 antibody, was designated as a breakthrough therapy in 2018 by the FDA for patients with advanced or metastatic RCC. The ORR was 63.3% for all RCC patients receiving this combination therapy, and 83.3% in the first-line treatment setting.^617^ Inspiringly, the combination of pembrolizumab with another small-molecule anti-angiogenesis agent axitinib has been approved for the treatment of patients with advanced RCC.^618,619^ This is also the first combination therapy of PD-1 antibody plus targeted drugs approved by the FDA for the first-line treatment of advanced RCC.

The third one is antibody-drug conjugate (ADC) drugs. Though the first ADC drug (Mylotarg, Pfizer) suffered setback in 2010 due to the limitations of coupling technology, targeting, and effectiveness, people did not lose confidence in ADC drugs.^620,621^ With the improvement of antibody-conjugate technology, ten ADC drugs such as polatuzumab vedotin-piiq (Polivy), enfortumab vedotin-ejfv (Padcev), fam-trastuzumab deruxtecan-nxki (Enhertu), sacituzumab govitecan-hziy (Trodelvy), and belantamab mafodotin-blmf (Blenrep) have been approved by the FDA in the past decade.^622–624^ ADC drugs will be an important development direction in the future.

The fourth one is PROTAC, which employs small molecules that recruit target proteins for ubiquitination and removal by the proteasome.^625^ PROTAC technology is different from the traditional “target occupying” inhibitor therapy. It reduces the activity of target proteins by catalyzing the degradation of target proteins. Due to the slow biosynthesis of target proteins, this method can greatly slow down the recovery of target protein activity. PROTAC is a rapidly emerging alternative therapeutic strategy with the potential to address many of the challenges currently faced in modern drug development programs. Currently, two drugs (ARV-110 and ARV-471) designed by PROTAC technology have entered clinical trials.^625^ Both of them are developed by Arvinas and approved by the FDA in 2019 for phase I clinical trials. Among them, ARV-110 specifically binds to androgen receptor (AR) and mediates AR degradation, and is used for the treatment of patients with metastatic CRPC.^626^ ARV-471 is an ER protein degrading agent. In preclinical studies, ARV-471 led to almost completely ER degradation in tumor cells and exhibited strong growth suppression in multiple ER-driven xenograft models.^627^ It is now evaluated clinically for the treatment of patients with locally advanced or metastatic ER^+^/HER2^-^ breast cancer.

The fifth one is synthetic lethality, which means that loss of function of either of the two genes individually has little effect on cancer cell viability, but the inactivation of both genes simultaneously by gene mutation/deletion or pharmacological inhibition leads to cell death.^628^ Normal cells are usually spared the effect of synthetic lethality due to the lack of the fixed genetic alteration. The concept of synthetic lethality has been used in the development of targeted therapy for many years. The most popular examples are the synthetic lethal interaction of combined BCL-XL and MEK inhibition in KRAS-mutant cancer models as well as the successful clinical application of PARP inhibitors in BRCA-mutant ovarian cancer.^574,629^ Although it is not a novel idea, synthetic lethality provides an alternative treatment strategy for some known genetic drivers of cancer that cannot be directly targeted owing to their molecular structure (undruggable oncogenes) or because they result in a functional loss (tumor suppressor genes). Therefore, synthetic lethality has promising potential to drive the discovery of new anti-cancer targets and subsequently the development of effective medicines or combination strategies that are still needed for most cancers.

In summary, small-molecule targeted drugs will continue to be the mainstream in cancer treatment because of their unique advantages, despite the competition from macromolecule drugs. With an in-depth understanding of tumor pathology and the evolution of new drug research and development technology, we believe that more new small-molecule anti-cancer drugs that target novel genes or the mechanism of action will be developed in the near future. It is also expected that some new areas such as the combination of small-molecule targeted drugs with tumor immunotherapy, ADC, and PROTAC will gain significant development in the next decade.

## Supplementary information

Supplemental Material File #1

## References

[1] Bedard PL, Hyman DM, Davids MS, Siu LL (2020). Small molecules, big impact: 20 years of targeted therapy in oncology. Lancet.

[2] Savage DG, Antman KH (2002). Imatinib mesylate–a new oral targeted therapy. N. Engl. J. Med..

[3] Wilkes GM (2018). Targeted therapy: attacking cancer with molecular and immunological targeted agents. Asia Pac. J. Oncol. Nurs..

[4] Lee YT, Tan YJ, Oon CE (2018). Molecular targeted therapy: treating cancer with specificity. Eur. J. Pharm..

[5] Ardito F (2017). The crucial role of protein phosphorylation in cell signaling and its use as targeted therapy (review). Int J. Mol. Med..

[6] Wilson LJ (2018). New perspectives, opportunities, and challenges in exploring the human protein kinome. Cancer Res..

[7] Roskoski R (2016). Classification of small molecule protein kinase inhibitors based upon the structures of their drug-enzyme complexes. Pharm. Res..

[8] Morris SW (1995). Fusion of a kinase gene, ALK, to a nucleolar protein gene, NPM, in non-Hodgkin’s lymphoma. Science.

[9] Iwahara T (1997). Molecular characterization of ALK, a receptor tyrosine kinase expressed specifically in the nervous system. Oncogene.

[10] McManus DT (2004). ALK-positive diffuse large B-cell lymphoma of the stomach associated with a clathrin-ALK rearrangement. Hum. Pathol..

[11] Cessna MH (2002). Expression of ALK1 and p80 in inflammatory myofibroblastic tumor and its mesenchymal mimics: a study of 135 cases. Mod. Pathol..

[12] Soda M (2007). Identification of the transforming EML4-ALK fusion gene in non-small-cell lung cancer. Nature.

[13] Waggott W (1995). Detection of NPM-ALK DNA rearrangement in CD30 positive anaplastic large cell lymphoma. Br. J. Haematol..

[14] Trinei M (2000). A new variant anaplastic lymphoma kinase (ALK)-fusion protein (ATIC-ALK) in a case of ALK-positive anaplastic large cell lymphoma. Cancer Res..

[15] Ma Z (2003). Fusion of ALK to the Ran-binding protein 2 (RANBP2) gene in inflammatory myofibroblastic tumor. Genes Chromosomes Cancer.

[16] Kong X (2019). Drug discovery targeting anaplastic lymphoma kinase (ALK). J. Med. Chem..

[17] Cui JJ (2011). Structure based drug design of crizotinib (PF-02341066), a potent and selective dual inhibitor of mesenchymal-epithelial transition factor (c-MET) kinase and anaplastic lymphoma kinase (ALK). J. Med. Chem..

[18] Shaw AT (2013). Crizotinib versus chemotherapy in advanced ALK-positive lung cancer. N. Engl. J. Med..

[19] Solomon BJ (2014). First-line crizotinib versus chemotherapy in ALK-positive lung cancer. N. Engl. J. Med..

[20] Sasaki T (2011). A novel ALK secondary mutation and EGFR signaling cause resistance to ALK kinase inhibitors. Cancer Res..

[21] Costa DB (2015). Clinical experience with crizotinib in patients with advanced ALK-rearranged non-small-cell lung cancer and brain metastases. J. Clin. Oncol..

[22] Marsilje TH (2013). Synthesis, structure-activity relationships, and in vivo efficacy of the novel potent and selective anaplastic lymphoma kinase (ALK) inhibitor 5-chloro-N2-(2-isopropoxy-5-methyl-4-(piperidin-4-yl)phenyl)-N4-(2-(isopropylsulf onyl)phenyl)pyrimidine-2,4-diamine (LDK378) currently in phase 1 and phase 2 clinical trials. J. Med. Chem..

[23] Kinoshita K (2011). 9-substituted 6,6-dimethyl-11-oxo-6,11-dihydro-5H-benzo[b]carbazoles as highly selective and potent anaplastic lymphoma kinase inhibitors. J. Med. Chem..

[24] Huang WS (2016). Discovery of brigatinib (AP26113), a phosphine oxide-containing, potent, orally active inhibitor of anaplastic lymphoma kinase. J. Med. Chem..

[25] Friboulet L (2014). The ALK inhibitor ceritinib overcomes crizotinib resistance in non-small cell lung cancer. Cancer Discov..

[26] Markham A (2017). Brigatinib: first global approval. Drugs.

[27] Qian M, Zhu B, Wang X, Liebman M (2017). Drug resistance in ALK-positiveNon-small cell lungcancer patients. Semin. Cell Dev. Biol..

[28] Basit S, Ashraf Z, Lee K, Latif M (2017). First macrocyclic 3(rd)-generation ALK inhibitor for treatment of ALK/ROS1 cancer: clinical and designing strategy update of lorlatinib. Eur. J. Med. Chem..

[29] Syed YY (2019). Lorlatinib: first global approval. Drugs.

[30] Guan J (2016). The ALK inhibitor PF-06463922 is effective as a single agent in neuroblastoma driven by expression of ALK and MYCN. Dis. Model Mech..

[31] Drilon A (2017). Safety and antitumor activity of the multitargeted pan-TRK, ROS1, and ALK inhibitor entrectinib: combined results from two phase I trials (ALKA-372-001 and STARTRK-1). Cancer Discov..

[32] Sachdev J (2014). 506 Phase (Ph) 1/2a study of TSR-011, a potent inhibitor of ALK and TRK, in advanced solid tumors including crizotinib-resistant ALK positive non-small cell lung cancer. Eur. J. Cancer.

[33] Drilon A (2018). Repotrectinib (TPX-0005) Is a next-generation ROS1/TRK/ALK inhibitor that potently inhibits ROS1/TRK/ALK solvent-front mutations. Cancer Discov..

[34] Menichincheri M (2016). Discovery of entrectinib: a new 3-aminoindazole as a potent anaplastic lymphoma kinase (ALK), c-ros oncogene 1 kinase (ROS1), and pan-tropomyosin receptor kinases (Pan-TRKs) inhibitor. J. Med. Chem..

[35] Infante JR (2012). Safety, pharmacokinetic, and pharmacodynamic phase I dose-escalation trial of PF-00562271, an inhibitor of focal adhesion kinase, in advanced solid tumors. J. Clin. Oncol..

[36] Shaw AT (2016). Resensitization to crizotinib by the lorlatinib ALK resistance mutation L1198F. N. Engl. J. Med..

[37] Chen Z (2010). Inhibition of ALK, PI3K/MEK, and HSP90 in murine lung adenocarcinoma induced by EML4-ALK fusion oncogene. Cancer Res..

[38] Roskoski R (2017). Anaplastic lymphoma kinase (ALK) inhibitors in the treatment of ALK-driven lung cancers. Pharm. Res..

[39] Courtin A (2016). Emergence of resistance to tyrosine kinase inhibitors in non-small-cell lung cancer can be delayed by an upfront combination with the HSP90 inhibitor onalespib. Br. J. Cancer.

[40] Zhang C (2018). Proteolysis targeting chimeras (PROTACs) of anaplastic lymphoma kinase (ALK). Eur. J. Med. Chem..

[41] Park M (1986). Mechanism of met oncogene activation. Cell.

[42] Baldanzi G, Graziani A (2014). Physiological signaling and structure of the HGF receptor MET. Biomedicines.

[43] Holmes O (2007). Insights into the structure/function of hepatocyte growth factor/scatter factor from studies with individual domains. J. Mol. Biol..

[44] Organ SL, Tsao MS (2011). An overview of the c-MET signaling pathway. Ther. Adv. Med. Oncol..

[45] Blumenschein GR, Mills GB, Gonzalez-Angulo AM (2012). Targeting the hepatocyte growth factor-cMET axis in cancer therapy. J. Clin. Oncol..

[46] Turke AB (2010). Preexistence and clonal selection of MET amplification in EGFR mutant NSCLC. Cancer Cell.

[47] Frampton GM (2015). Activation of MET via diverse exon 14 splicing alterations occurs in multiple tumor types and confers clinical sensitivity to MET inhibitors. Cancer Discov..

[48] Garajova I, Giovannetti E, Biasco G, Peters GJ (2015). c-Met as a Target for Personalized Therapy. Transl. Oncogenomics.

[49] Heigener DF, Reck M (2018). Crizotinib. Recent Results Cancer Res..

[50] Choueiri TK (2016). Cabozantinib versus everolimus in advanced renal cell carcinoma (METEOR): final results from a randomised, open-label, phase 3 trial. Lancet Oncol..

[51] Liu X (2011). A novel kinase inhibitor, INCB28060, blocks c-MET-dependent signaling, neoplastic activities, and cross-talk with EGFR and HER-3. Clin. Cancer Res..

[52] Wolf J (2020). Capmatinib in MET exon 14-mutated or MET-amplified non-small-cell lung cancer. N. Engl. J. Med..

[53] Wu YL (2018). Phase Ib/II study of capmatinib (INC280) plus gefitinib after failure of epidermal growth factor receptor (EGFR) inhibitor therapy in patients with EGFR-mutated, MET factor-dysregulated non-small-cell lung cancer. J. Clin. Oncol..

[54] Lara MS (2017). Preclinical evaluation of MET inhibitor INC-280 with or without the epidermal growth factor receptor inhibitor erlotinib in non-small-cell lung cancer. Clin. Lung Cancer.

[55] Bladt F (2013). EMD 1214063 and EMD 1204831 constitute a new class of potent and highly selective c-Met inhibitors. Clin. Cancer Res..

[56] Friese-Hamim M (2017). The selective c-Met inhibitor tepotinib can overcome epidermal growth factor receptor inhibitor resistance mediated by aberrant c-Met activation in NSCLC models. Am. J. Cancer Res..

[57] Markham A (2020). Tepotinib: first approval. Drugs.

[58] Leighl NB (2017). A phase I study of foretinib plus erlotinib in patients with previously treated advanced non-small cell lung cancer: Canadian cancer trials group IND.196. Oncotarget.

[59] Engstrom LD (2017). Glesatinib exhibits antitumor activity in lung cancer models and patients harboring MET exon 14 mutations and overcomes mutation-mediated resistance to type I MET inhibitors in nonclinical models. Clin. Cancer Res..

[60] Miranda, O., Farooqui, M. & Siegfried, J. M. Status of agents targeting the HGF/c-met axis in lung cancer. *Cancers* **10**, 280 (2018). 10.3390/cancers10090280 PMC6162713 30134579

[61] Rodon J (2017). First-in-human phase I study of oral S49076, a unique MET/AXL/FGFR inhibitor, in advanced solid tumours. Eur. J. Cancer.

[62] Sequist LV (2011). Randomized phase II study of erlotinib plus tivantinib versus erlotinib plus placebo in previously treated non-small-cell lung cancer. J. Clin. Oncol..

[63] Yang J-J (2016). Preliminary results of a phase Ib trial of savolitinib combined with gefitinib in EGFR-mutant lung cancer. J. Clin. Oncol..

[64] Egile C (2015). The selective intravenous inhibitor of the MET tyrosine kinase SAR125844 inhibits tumor growth in MET-amplified cancer. Mol. Cancer Ther..

[65] Shitara K (2017). Phase I dose-escalation study of the c-Met tyrosine kinase inhibitor SAR125844 in Asian patients with advanced solid tumors, including patients with MET-amplified gastric cancer. Oncotarget.

[66] Parikh PK, Ghate MD (2018). Recent advances in the discovery of small molecule c-Met Kinase inhibitors. Eur. J. Med. Chem..

[67] Bradley CA (2017). Targeting c-MET in gastrointestinal tumours: rationale, opportunities and challenges. Nat. Rev. Clin. Oncol..

[68] Salgia R (2017). MET in lung cancer: biomarker selection based on scientific rationale. Mol. Cancer Ther..

[69] Qi J (2011). Multiple mutations and bypass mechanisms can contribute to development of acquired resistance to MET inhibitors. Cancer Res..

[70] Cepero V (2010). MET and KRAS gene amplification mediates acquired resistance to MET tyrosine kinase inhibitors. Cancer Res..

[71] Threadgill DW (1995). Targeted disruption of mouse EGF receptor: effect of genetic background on mutant phenotype. Science.

[72] Blobel CP (2005). ADAMs: key components in EGFR signalling and development. Nat. Rev. Mol. Cell Biol..

[73] Salomon DS, Brandt R, Ciardiello F, Normanno N (1995). Epidermal growth factor-related peptides and their receptors in human malignancies. Crit. Rev. Oncol./Hematol..

[74] Metro G (2006). Epidermal growth factor receptor (EGFR) targeted therapies in non-small cell lung cancer (NSCLC). Rev. Recent Clin. trials.

[75] Roskoski R (2014). The ErbB/HER family of protein-tyrosine kinases and cancer. Pharm. Res..

[76] Gu A (2013). Efficacy and safety evaluation of icotinib in patients with advanced non-small cell lung cancer. Chin. J. Cancer Res..

[77] Lynch TJ (2004). Activating mutations in the epidermal growth factor receptor underlying responsiveness of non-small-cell lung cancer to gefitinib. N. Engl. J. Med..

[78] Kulke MH (2007). Capecitabine plus erlotinib in gemcitabine-refractory advanced pancreatic cancer. J. Clin. Oncol..

[79] Maione P (2015). Overcoming resistance to targeted therapies in NSCLC: current approaches and clinical application. Ther. Adv. Med. Oncol..

[80] Miller VA (2012). Afatinib versus placebo for patients with advanced, metastatic non-small-cell lung cancer after failure of erlotinib, gefitinib, or both, and one or two lines of chemotherapy (LUX-Lung 1): a phase 2b/3 randomised trial. Lancet Oncol..

[81] Lavacchi D, Mazzoni F, Giaccone G (2019). Clinical evaluation of dacomitinib for the treatment of metastatic non-small cell lung cancer (NSCLC): current perspectives. Drug Des. Dev. Ther..

[82] Schrank, Z. et al. Current molecular-targeted therapies in NSCLC and their mechanism of resistance. *Cancers* **10**, 224 (2018). 10.3390/cancers10070224 PMC6071023 29973561

[83] Yver A (2016). Osimertinib (AZD9291)-a science-driven, collaborative approach to rapid drug design and development. Ann. Oncol..

[84] Yang, J. C. et al. Safety, efficacy, and pharmacokinetics of almonertinib (HS-10296) in pretreated patients with EGFR-mutated advanced NSCLC: a multicenter, open-label, phase 1 trial. *J. Thorac. Oncol*. **15**, 1907–1918 (2020). 10.1016/j.jtho.2020.09.001 32916310

[85] Paul B, Trovato JA, Thompson J (2008). Lapatinib: a dual tyrosine kinase inhibitor for metastatic breast cancer. Am. J. Health Syst. Pharm..

[86] Deeks ED (2017). Neratinib: first global approval. Drugs.

[87] Murthy RK (2020). Tucatinib, trastuzumab, and capecitabine for HER2-positive metastatic breast cancer. N. Engl. J. Med..

[88] Kim ES (2016). Olmutinib: first global approval. Drugs.

[89] Roskoski R (2019). Small molecule inhibitors targeting the EGFR/ErbB family of protein-tyrosine kinases in human cancers. Pharm. Res..

[90] Kim DW (2019). Safety, tolerability, and anti-tumor activity of olmutinib in non-small cell lung cancer with T790M mutation: a single arm, open label, phase 1/2 trial. Lung Cancer.

[91] Ma Y (2018). First-in-human phase I study of AC0010, a mutant-selective EGFR inhibitor in non-small cell lung cancer: safety, efficacy, and potential mechanism of resistance. J. Thorac. Oncol..

[92] Erlichman C (2006). Phase I study of EKB-569, an irreversible inhibitor of the epidermal growth factor receptor, in patients with advanced solid tumors. J. Clin. Oncol..

[93] Thress KS (2015). Acquired EGFR C797S mutation mediates resistance to AZD9291 in non-small cell lung cancer harboring EGFR T790M. Nat. Med..

[94] Zhou Z (2019). Durable clinical response of lung adenocarcinoma harboring EGFR 19Del/T790M/in trans-C797S to combination therapy of first- and third-generation EGFR tyrosine kinase inhibitors. J. Thorac. Oncol..

[95] Jia Y (2016). Overcoming EGFR(T790M) and EGFR(C797S) resistance with mutant-selective allosteric inhibitors. Nature.

[96] Lu X (2018). Discovery of JND3229 as a new EGFR(C797S) mutant inhibitor with in vivo monodrug efficacy. ACS Med. Chem. Lett..

[97] Shen, J. et al. Structure-based design of 5-methylpyrimidopyridone derivatives as new wild-type sparing inhibitors of the epidermal growth factor receptor triple mutant (EGFR(L858R/T790M/C797S). *J. Med. Chem.* **62**, 7302–7308 (2019). 10.1021/acs.jmedchem.9b00576 31298540

[98] Gilliland DG, Griffin JD (2002). The roles of FLT3 in hematopoiesis and leukemia. Blood.

[99] Kiyoi H (2002). Mechanism of constitutive activation of FLT3 with internal tandem duplication in the juxtamembrane domain. Oncogene.

[100] Quentmeier H, Reinhardt J, Zaborski M, Drexler HG (2003). FLT3 mutations in acute myeloid leukemia cell lines. Leukemia.

[101] Tallman MS, Gilliland DG, Rowe JM (2005). Drug therapy for acute myeloid leukemia. Blood.

[102] Daver N, Schlenk RF, Russell NH, Levis MJ (2019). Targeting FLT3 mutations in AML: review of current knowledge and evidence. Leukemia.

[103] Auclair D (2007). Antitumor activity of sorafenib in FLT3-driven leukemic cells. Leukemia.

[104] Stone RM (2017). Midostaurin plus chemotherapy for acute myeloid leukemia with a FLT3 mutation. N. Engl. J. Med..

[105] Garcia JS, Stone RM (2017). The development of FLT3 inhibitors in acute myeloid leukemia. Hematol. Oncol. Clin. North Am..

[106] Knapper S (2011). The clinical development of FLT3 inhibitors in acute myeloid leukemia. Expert Opin. Investig. Drugs.

[107] Baldi GG, Gronchi A, Stacchiotti S (2020). Pexidartinib for the treatment of adult symptomatic patients with tenosynovial giant cell tumors. Expert Rev. Clin. Pharm..

[108] Mori M (2017). Gilteritinib, a FLT3/AXL inhibitor, shows antileukemic activity in mouse models of FLT3 mutated acute myeloid leukemia. Invest. N. Drugs.

[109] Zarrinkar PP (2009). AC220 is a uniquely potent and selective inhibitor of FLT3 for the treatment of acute myeloid leukemia (AML). Blood.

[110] Cortes J (2018). Quizartinib, an FLT3 inhibitor, as monotherapy in patients with relapsed or refractory acute myeloid leukaemia: an open-label, multicentre, single-arm, phase 2 trial. Lancet Oncol..

[111] Galanis A (2014). Crenolanib is a potent inhibitor of FLT3 with activity against resistance-conferring point mutants. Blood.

[112] Cao ZX (2012). SKLB1028, a novel oral multikinase inhibitor of EGFR, FLT3 and Abl, displays exceptional activity in models of FLT3-driven AML and considerable potency in models of CML harboring Abl mutants. Leukemia.

[113] Sutamtewagul G, Vigil CE (2018). Clinical use of FLT3 inhibitors in acute myeloid leukemia. Onco Targets Ther..

[114] Lim SH, Dubielecka PM, Raghunathan VM (2017). Molecular targeting in acute myeloid leukemia. J. Transl. Med..

[115] Miller GD, Bruno BJ, Lim CS (2014). Resistant mutations in CML and Ph(+)ALL - role of ponatinib. Biologics.

[116] Elshoury A, Przespolewski A, Baron J, Wang ES (2019). Advancing treatment of acute myeloid leukemia: the future of FLT3 inhibitors. Expert Rev. Anticancer Ther..

[117] Smith CC (2012). Validation of ITD mutations in FLT3 as a therapeutic target in human acute myeloid leukaemia. Nature.

[118] Sung L (2012). Predictors and short-term outcomes of hyperleukocytosis in children with acute myeloid leukemia: a report from the Children’s Oncology Group. Haematologica.

[119] Lin WH (2014). Evaluation of the antitumor effects of BPR1J-340, a potent and selective FLT3 inhibitor, alone or in combination with an HDAC inhibitor, vorinostat, in AML cancer. PLoS ONE.

[120] Larrosa-Garcia M, Baer MR (2017). FLT3 inhibitors in acute myeloid leukemia: current status and future directions. Mol. Cancer Ther..

[121] Cao T (2019). The FLT3-ITD mutation and the expression of its downstream signaling intermediates STAT5 and Pim-1 are positively correlated with CXCR4 expression in patients with acute myeloid leukemia. Sci. Rep..

[122] Uras IZ (2016). Palbociclib treatment of FLT3-ITD+ AML cells uncovers a kinase-dependent transcriptional regulation of FLT3 and PIM1 by CDK6. Blood.

[123] Sandhofer N (2015). Dual PI3K/mTOR inhibition shows antileukemic activity in MLL-rearranged acute myeloid leukemia. Leukemia.

[124] Yamaura T (2018). A novel irreversible FLT3 inhibitor, FF-10101, shows excellent efficacy against AML cells with FLT3 mutations. Blood.

[125] Chen CT (2015). Identification of a potent 5-phenyl-thiazol-2-ylamine-based inhibitor of FLT3 with activity against drug resistance-conferring point mutations. Eur. J. Med. Chem..

[126] Smith CC (2015). Characterizing and overriding the structural mechanism of the quizartinib-resistant FLT3 “Gatekeeper” F691L mutation with PLX3397. Cancer Discov..

[127] Xu B (2017). MZH29 is a novel potent inhibitor that overcomes drug resistance FLT3 mutations in acute myeloid leukemia. Leukemia.

[128] Park I (2003). Angiogenesis and microsatellite alterations in oral cavity and oropharynx cancer. Otolaryngol. Head Neck Surg..

[129] Risau W (1997). Mechanisms of angiogenesis. Nature.

[130] Klagsbrun M, Moses MA (1999). Molecular angiogenesis. Chem. Biol..

[131] Yadav L (2015). Tumour angiogenesis and angiogenic inhibitors: a review. J. Clin. Diagn. Res..

[132] Yang WH, Xu J, Mu JB, Xie J (2017). Revision of the concept of anti-angiogenesis and its applications in tumor treatment. Chronic Dis. Transl. Med..

[133] Folkman J (1971). Tumor angiogenesis - therapeutic implications. N. Engl. J. Med..

[134] Potente M, Gerhardt H, Carmeliet P (2011). Basic and therapeutic aspects of angiogenesis. Cell.

[135] Hanahan D, Folkman J (1996). Patterns and emerging mechanisms of the angiogenic switch during tumorigenesis. Cell.

[136] Kerbel RS (2000). Tumor angiogenesis: past, present and the near future. Carcinogenesis.

[137] Fagiani E, Christofori G (2013). Angiopoietins in angiogenesis. Cancer Lett..

[138] Zheng X (2018). The regulation of cytokine signaling by retinal determination gene network pathway in cancer. Onco Targets Ther..

[139] Leung DW (1989). Vascular endothelial growth factor is a secreted angiogenic mitogen. Science.

[140] Itoh N, Ornitz DM (2004). Evolution of the Fgf and Fgfr gene families. Trends Genet..

[141] Chen PH, Chen X, He X (2013). Platelet-derived growth factors and their receptors: structural and functional perspectives. Biochim. Biophys. Acta.

[142] Shi Y, Massagué J (2003). Mechanisms of TGF-β signaling from cell membrane to the nucleus. Cell.

[143] Rak J, Yu JL, Klement G, Kerbel RS (2000). Oncogenes and angiogenesis: signaling three-dimensional tumor growth. J. Investig. Dermatol Symp. Proc..

[144] Ferrara N, Davis-Smyth T (1997). The biology of vascular endothelial growth factor. Endocr. Rev..

[145] Eskens FA, Verweij J (2006). The clinical toxicity profile of vascular endothelial growth factor (VEGF) and vascular endothelial growth factor receptor (VEGFR) targeting angiogenesis inhibitors; a review. Eur. J. Cancer.

[146] Kerbel R, Folkman J (2002). Clinical translation of angiogenesis inhibitors. Nat. Rev. Cancer.

[147] Wilhelm SM (2008). Preclinical overview of sorafenib, a multikinase inhibitor that targets both Raf and VEGF and PDGF receptor tyrosine kinase signaling. Mol. Cancer Ther..

[148] Llovet JM (2008). Sorafenib in advanced hepatocellular carcinoma. N. Engl. J. Med..

[149] Wilhelm S (2006). Discovery and development of sorafenib: a multikinase inhibitor for treating cancer. Nat. Rev. Drug Discov..

[150] Woo HY, Heo J (2012). Sorafenib in liver cancer. Expert Opin. Pharmacother..

[151] Escudier B (2007). Sorafenib in advanced clear-cell renal-cell carcinoma. N. Engl. J. Med..

[152] Brose MS (2014). Sorafenib in radioactive iodine-refractory, locally advanced or metastatic differentiated thyroid cancer: a randomised, double-blind, phase 3 trial. Lancet.

[153] Motzer RJ, Escudier B, Gannon A, Figlin RA (2017). Sunitinib: ten years of successful clinical use and study in advanced renal cell carcinoma. Oncologist.

[154] van der Graaf WTA (2012). Pazopanib for metastatic soft-tissue sarcoma (PALETTE): a randomised, double-blind, placebo-controlled phase 3 trial. Lancet.

[155] Keating GM (2015). Axitinib: a review in advanced renal cell carcinoma. Drugs.

[156] Elisei R (2013). Cabozantinib in progressive medullary thyroid cancer. J. Clin. Oncol..

[157] Escudier B (2018). The role of tivozanib in advanced renal cell carcinoma therapy. Expert Rev. Anticancer Ther..

[158] Schenone S, Brullo C, Botta M (2008). Small molecules ATP-competitive inhibitors of FLT3: a chemical overview. Curr. Med. Chem..

[159] Roskoski R (2007). Sunitinib: a VEGF and PDGF receptor protein kinase and angiogenesis inhibitor. Biochem. Biophys. Res. Commun..

[160] Morabito A (2010). Vandetanib: an overview of its clinical development in NSCLC and other tumors. Drugs Today.

[161] Yoh K (2017). Vandetanib in patients with previously treated RET-rearranged advanced non-small-cell lung cancer (LURET): an open-label, multicentre phase 2 trial. Lancet Respir. Med..

[162] Zhang Y (2010). XL-184, a MET, VEGFR-2 and RET kinase inhibitor for the treatment of thyroid cancer, glioblastoma multiforme and NSCLC. IDrugs Investig. Drugs J..

[163] Markham A (2020). Selpercatinib: first approval. Drugs.

[164] Markham A (2020). Pralsetinib: first approval. Drugs.

[165] Wilhelm SM (2011). Regorafenib (BAY 73-4506): a new oral multikinase inhibitor of angiogenic, stromal and oncogenic receptor tyrosine kinases with potent preclinical antitumor activity. Int J. Cancer.

[166] Strumberg D (2012). Regorafenib (BAY 73-4506) in advanced colorectal cancer: a phase I study. Br. J. Cancer.

[167] Demetri GD (2013). Efficacy and safety of regorafenib for advanced gastrointestinal stromal tumours after failure of imatinib and sunitinib (GRID): an international, multicentre, randomised, placebo-controlled, phase 3 trial. Lancet.

[168] Dhillon S (2015). Nintedanib: a review of its use as second-line treatment in adults with advanced non-small cell lung cancer of adenocarcinoma histology. Target Oncol..

[169] Keating GM (2015). Nintedanib: a review of its use in patients with idiopathic pulmonary fibrosis. Drugs.

[170] Alshangiti A, Chandhoke G, Ellis PM (2018). Antiangiogenic therapies in non-small-cell lung cancer. Curr. Oncol..

[171] Aoyama T, Yoshikawa T (2016). Targeted therapy: apatinib - new third-line option for refractory gastric or GEJ cancer. Nat. Rev. Clin. Oncol..

[172] Syed YY (2018). Anlotinib: first global approval. Drugs.

[173] Han B (2018). Anlotinib as a third-line therapy in patients with refractory advanced non-small-cell lung cancer: a multicentre, randomised phase II trial (ALTER0302). Br. J. Cancer.

[174] Shirley M (2018). Fruquintinib: first global approval. Drugs.

[175] Li J (2018). Effect of fruquintinib vs placebo on overall survival in patients with previously treated metastatic colorectal cancer: The FRESCO Randomized Clinical Trial. JAMA.

[176] Chen Z, Jiang L (2019). The clinical application of fruquintinib on colorectal cancer. Expert Rev. Clin. Pharm..

[177] Perera TPS (2017). Discovery and pharmacological characterization of JNJ-42756493 (Erdafitinib), a functionally selective small-molecule FGFR family inhibitor. Mol. Cancer Ther..

[178] Zhang Y (2005). Constitutive activating mutation of the FGFR3b in oral squamous cell carcinomas. Int J. Cancer.

[179] Hoy SM (2020). Pemigatinib: first approval. Drugs.

[180] Okamoto I (2020). Comparison of carboplatin plus pemetrexed followed by maintenance pemetrexed with docetaxel monotherapy in elderly patients with advanced nonsquamous non-small cell lung cancer: A Phase 3 Randomized Clinical Trial. JAMA Oncol..

[181] Dhillon S (2020). Avapritinib: first approval. Drugs.

[182] Kasireddy V, von Mehren M (2017). Emerging drugs for the treatment of gastrointestinal stromal tumour. Expert Opin. Emerg. Drugs.

[183] Heinrich MC (2020). Avapritinib in advanced PDGFRA D842V-mutant gastrointestinal stromal tumour (NAVIGATOR): a multicentre, open-label, phase 1 trial. Lancet Oncol..

[184] Saleh, N. Avapritinib approved for GIST subgroup. *Cancer Discov*. **10**, 334 (2020). 10.1158/2159-8290.CD-NB2020-002 31974168

[185] Dhillon S (2020). Ripretinib: first approval. Drugs.

[186] Batchelor TT (2010). Phase II study of cediranib, an oral pan-vascular endothelial growth factor receptor tyrosine kinase inhibitor, in patients with recurrent glioblastoma. J. Clin. Oncol..

[187] Liu JF (2014). Combination cediranib and olaparib versus olaparib alone for women with recurrent platinum-sensitive ovarian cancer: a randomised phase 2 study. Lancet Oncol..

[188] Renhowe PA (2009). Design, structure-activity relationships and in vivo characterization of 4-amino-3-benzimidazol-2-ylhydroquinolin-2-ones: a novel class of receptor tyrosine kinase inhibitors. J. Med. Chem..

[189] Kim KB (2011). Phase I/II and pharmacodynamic study of dovitinib (TKI258), an inhibitor of fibroblast growth factor receptors and VEGF receptors, in patients with advanced melanoma. Clin. Cancer Res..

[190] Raghav KP, Blumenschein GR (2011). Motesanib and advanced NSCLC: experiences and expectations. Expert Opin. Investig. Drugs.

[191] Kubota K (2017). Phase III, randomized, placebo-controlled, double-blind trial of motesanib (AMG-706) in combination with paclitaxel and carboplatin in East Asian patients with advanced nonsquamous non-small-cell lung cancer. J. Clin. Oncol..

[192] Schlumberger MJ (2009). Phase II study of safety and efficacy of motesanib in patients with progressive or symptomatic, advanced or metastatic medullary thyroid cancer. J. Clin. Oncol..

[193] Bass MB (2010). Biomarkers as predictors of response to treatment with motesanib in patients with progressive advanced thyroid cancer. J. Clin. Endocrinol. Metab..

[194] Sherman SI (2008). Motesanib diphosphate in progressive differentiated thyroid cancer. N. Engl. J. Med..

[195] Xu JM (2017). Sulfatinib, a novel kinase inhibitor, in patients with advanced solid tumors: results from a phase I study. Oncotarget.

[196] Xu J (2019). Surufatinib in advanced well-differentiated neuroendocrine tumors: A Multicenter, Single-arm, Open-label, Phase Ib/II Trial. Clin. Cancer Res..

[197] Xu, J. et al. Surufatinib in advanced extrapancreatic neuroendocrine tumours (SANET-ep): a randomised, double-blind, placebo-controlled, phase 3 study. *Lancet Oncol.* **21**, 1500–1512 (2020). 10.1016/S1470-2045(20)30496-4 32966811

[198] Heinrich MC (2012). Crenolanib inhibits the drug-resistant PDGFRA D842V mutation associated with imatinib-resistant gastrointestinal stromal tumors. Clin. Cancer Res..

[199] Indio, V. et al. Integrated molecular characterization of gastrointestinal stromal tumors (GIST) harboring the rare D842V mutation in PDGFRA gene. *Int. J. Mol. Sci*. **19**, 732 (2018). 10.3390/ijms19030732 PMC5877593 29510530

[200] Jetani H (2018). CAR T-cells targeting FLT3 have potent activity against FLT3(-)ITD(+) AML and act synergistically with the FLT3-inhibitor crenolanib. Leukemia.

[201] Guffanti F (2017). In vitro and in vivo activity of lucitanib in FGFR1/2 amplified or mutated cancer models. Neoplasia.

[202] Cai ZW (2008). Discovery of brivanib alaninate ((S)-((R)-1-(4-(4-fluoro-2-methyl-1H-indol-5-yloxy)-5-methylpyrrolo[2,1-f][1,2,4]triazin-6-yloxy)propan-2-yl)2-aminopropanoate), a novel prodrug of dual vascular endothelial growth factor receptor-2 and fibroblast growth factor receptor-1 kinase inhibitor (BMS-540215). J. Med. Chem..

[203] Johnson PJ (2013). Brivanib versus sorafenib as first-line therapy in patients with unresectable, advanced hepatocellular carcinoma: results from the randomized phase III BRISK-FL study. J. Clin. Oncol..

[204] Kudo M (2014). Brivanib as adjuvant therapy to transarterial chemoembolization in patients with hepatocellular carcinoma: a randomized phase III trial. Hepatology.

[205] Tucker JA (2014). Structural insights into FGFR kinase isoform selectivity: diverse binding modes of AZD4547 and ponatinib in complex with FGFR1 and FGFR4. Structure.

[206] Felix NS (2019). Effects of the FGF receptor-1 inhibitor, infigratinib, with or without sildenafil, in experimental pulmonary arterial hypertension. Br. J. Pharm..

[207] Makawita S (2020). Infigratinib in patients with advanced cholangiocarcinoma with FGFR2 gene fusions/translocations: the PROOF 301 trial. Future Oncol..

[208] Lahn M, Kloeker S, Berry BS (2005). TGF-beta inhibitors for the treatment of cancer. Expert Opin. Investig. Drugs.

[209] Herbertz S (2015). Clinical development of galunisertib (LY2157299 monohydrate), a small molecule inhibitor of transforming growth factor-beta signaling pathway. Drug Des. Dev. Ther..

[210] Jung SY (2020). Population pharmacokinetics of vactosertib, a new TGF-beta receptor type Iota inhibitor, in patients with advanced solid tumors. Cancer Chemother. Pharm..

[211] Xu G (2020). Synthesis and biological evaluation of 4-(pyridin-4-oxy)-3-(3,3-difluorocyclobutyl)-pyrazole derivatives as novel potent transforming growth factor-beta type 1 receptor inhibitors. Eur. J. Med. Chem..

[212] Yingling JM (2018). Preclinical assessment of galunisertib (LY2157299 monohydrate), a first-in-class transforming growth factor-β receptor type I inhibitor. Oncotarget.

[213] Santini V (2019). Phase II study of the ALK5 inhibitor galunisertib in very low-, low-, and intermediate-risk myelodysplastic syndromes. Clin. Cancer Res..

[214] Holmgaard RB (2018). Targeting the TGFbeta pathway with galunisertib, a TGFbetaRI small molecule inhibitor, promotes anti-tumor immunity leading to durable, complete responses, as monotherapy and in combination with checkpoint blockade. J. Immunother. Cancer.

[215] Teleanu, R. I., Chircov, C., Grumezescu, A. M. & Teleanu, D. M. Tumor angiogenesis and anti-angiogenic strategies for cancer treatment. *J. Clin. Med*. **9**, 84 (2019). 10.3390/jcm9010084 PMC7020037 31905724

[216] Rajabi, M. & Mousa, S. A. The role of angiogenesis in cancer treatment. *Biomedicines*. **5**, 34 (2017). 10.3390/biomedicines5020034 PMC5489820 28635679

[217] Russo M, Giavazzi R (2018). Anti-angiogenesis for cancer: current status and prospects. Thromb. Res..

[218] Mauceri HJ (1998). Combined effects of angiostatin and ionizing radiation in antitumour therapy. Nature.

[219] Hlatky L, Hahnfeldt P, Folkman J (2002). Clinical application of antiangiogenic therapy: microvessel density, what it does and doesn’t tell us. J. Natl Cancer Inst..

[220] Giuliano S, Pagès G (2013). Mechanisms of resistance to anti-angiogenesis therapies. Biochimie.

[221] Posadas EM, Limvorasak S, Figlin RA (2017). Targeted therapies for renal cell carcinoma. Nat. Rev. Nephrol..

[222] Sheng X (2019). Axitinib in combination with toripalimab, a humanized immunoglobulin G(4) monoclonal antibody against programmed cell death-1, in patients with metastatic mucosal melanoma: An Open-Label Phase IB Trial. J. Clin. Oncol..

[223] Liu X (2017). Early presence of anti-angiogenesis-related adverse events as a potential biomarker of antitumor efficacy in metastatic gastric cancer patients treated with apatinib: a cohort study. J. Hematol. Oncol..

[224] Rini BI (2011). Diastolic blood pressure as a biomarker of axitinib efficacy in solid tumors. Clin. Cancer Res..

[225] Cocco E, Scaltriti M, Drilon A (2018). NTRK fusion-positive cancers and TRK inhibitor therapy. Nat. Rev. Clin. Oncol..

[226] Scott-Solomon E, Kuruvilla R (2018). Mechanisms of neurotrophin trafficking via Trk receptors. Mol. Cell. Neurosci..

[227] Nakagawara A (2001). Trk receptor tyrosine kinases: a bridge between cancer and neural development. Cancer Lett..

[228] Bertrand T (2012). The crystal structures of TrkA and TrkB suggest key regions for achieving selective inhibition. J. Mol. Biol..

[229] Drilon A (2018). Efficacy of larotrectinib in TRK fusion-positive cancers in adults and children. N. Engl. J. Med..

[230] Doebele RC (2020). Entrectinib in patients with advanced or metastatic NTRK fusion-positive solid tumours: integrated analysis of three phase 1-2 trials. Lancet Oncol..

[231] Ardini E (2016). Entrectinib, a pan-TRK, ROS1, and ALK inhibitor with activity in multiple molecularly defined cancer indications. Mol. Cancer Ther..

[232] Federman N, McDermott R (2019). Larotrectinib, a highly selective tropomyosin receptor kinase (TRK) inhibitor for the treatment of TRK fusion cancer. Expert Rev. Clin. Pharm..

[233] Grüllich C (2018). Cabozantinib: multi-kinase Inhibitor of MET, AXL, RET, and VEGFR2. Recent Results Cancer Res..

[234] Konicek BW (2018). Merestinib (LY2801653) inhibits neurotrophic receptor kinase (NTRK) and suppresses growth of NTRK fusion bearing tumors. Oncotarget.

[235] Lin CC (2019). A phase 1, open-label, dose-escalation trial of oral TSR-011 in patients with advanced solid tumours and lymphomas. Br. J. Cancer.

[236] Yang, Y. et al. Sitravatinib, a tyrosine kinase inhibitor, inhibits the transport function of ABCG2 and restores sensitivity to chemotherapy-resistant cancer cells in vitro. *Front. Oncol*. **10**, 700 (2020). 10.3389/fonc.2020.00700 PMC7236772 32477943

[237] Piao Y (2016). Novel MET/TIE2/VEGFR2 inhibitor altiratinib inhibits tumor growth and invasiveness in bevacizumab-resistant glioblastoma mouse models. Neuro-Oncol..

[238] Katayama R (2019). The new-generation selective ROS1/NTRK inhibitor DS-6051b overcomes crizotinib resistant ROS1-G2032R mutation in preclinical models. Nat. Commun..

[239] Khotskaya YB (2017). Targeting TRK family proteins in cancer. Pharmacol. Therap..

[240] Russo M (2016). Acquired resistance to the TRK inhibitor entrectinib in colorectal cancer. Cancer Discov..

[241] Drilon A (2017). A next-generation TRK kinase inhibitor overcomes acquired resistance to prior trk kinase inhibition in patients with TRK fusion-positive solid tumors. Cancer Discov..

[242] Drilon A (2019). TRK inhibitors in TRK fusion-positive cancers. Ann. Oncol..

[243] Quintas-Cardama A, Cortes J (2009). Molecular biology of bcr-abl1-positive chronic myeloid leukemia. Blood.

[244] Redaelli A (2004). Clinical and epidemiologic burden of chronic myelogenous leukemia. Expert Rev. Anticancer Ther..

[245] Kurzrock R, Kantarjian HM, Druker BJ, Talpaz M (2003). Philadelphia chromosome-positive leukemias: from basic mechanisms to molecular therapeutics. Ann. Intern. Med..

[246] Klein F (2004). The BCR-ABL1 kinase bypasses selection for the expression of a pre-B cell receptor in pre-B acute lymphoblastic leukemia cells. J. Exp. Med..

[247] Deininger M, Buchdunger E, Druker BJ (2005). The development of imatinib as a therapeutic agent for chronic myeloid leukemia. Blood.

[248] Druker BJ (2006). Five-year follow-up of patients receiving imatinib for chronic myeloid leukemia. N. Engl. J. Med..

[249] Hochhaus A (2002). Molecular and chromosomal mechanisms of resistance to imatinib (STI571) therapy. Leukemia.

[250] Hantschel O, Grebien F, Superti-Furga G (2012). The growing arsenal of ATP-competitive and allosteric inhibitors of BCR-ABL. Cancer Res..

[251] Azam M, Latek RR, Daley GQ (2003). Mechanisms of autoinhibition and STI-571/imatinib resistance revealed by mutagenesis of BCR-ABL. Cell.

[252] Jabbour E, Cortes J, Kantarjian H (2009). Treatment selection after imatinib resistance in chronic myeloid leukemia. Target Oncol..

[253] Weisberg E (2005). Characterization of AMN107, a selective inhibitor of native and mutant Bcr-Abl. Cancer Cell.

[254] Puttini M (2006). In vitro and in vivo activity of SKI-606, a novel Src-Abl inhibitor, against imatinib-resistant Bcr-Abl+ neoplastic cells. Cancer Res..

[255] Sawyers CL (2010). Even better kinase inhibitors for chronic myeloid leukemia. N. Engl. J. Med..

[256] O’Hare T (2005). In vitro activity of Bcr-Abl inhibitors AMN107 and BMS-354825 against clinically relevant imatinib-resistant Abl kinase domain mutants. Cancer Res..

[257] O’Hare T (2011). Targeting the BCR-ABL signaling pathway in therapy-resistant Philadelphia chromosome-positive leukemia. Clin. Cancer Res..

[258] O’Hare T (2009). AP24534, a pan-BCR-ABL inhibitor for chronic myeloid leukemia, potently inhibits the T315I mutant and overcomes mutation-based resistance. Cancer Cell.

[259] Wylie AA (2017). The allosteric inhibitor ABL001 enables dual targeting of BCR-ABL1. Nature.

[260] Eide CA (2011). The ABL switch control inhibitor DCC-2036 is active against the chronic myeloid leukemia mutant BCR-ABLT315I and exhibits a narrow resistance profile. Cancer Res..

[261] Yokota A (2007). INNO-406, a novel BCR-ABL/Lyn dual tyrosine kinase inhibitor, suppresses the growth of Ph+ leukemia cells in the central nervous system, and cyclosporine A augments its in vivo activity. Blood.

[262] Zabriskie MS (2014). BCR-ABL1 compound mutations combining key kinase domain positions confer clinical resistance to ponatinib in Ph chromosome-positive leukemia. Cancer Cell.

[263] O’Hare T, Zabriskie MS, Eiring AM, Deininger MW (2012). Pushing the limits of targeted therapy in chronic myeloid leukaemia. Nat. Rev. Cancer.

[264] Gibbons DL (2014). Molecular dynamics reveal BCR-ABL1 polymutants as a unique mechanism of resistance to PAN-BCR-ABL1 kinase inhibitor therapy. Proc. Natl Acad. Sci. USA.

[265] Alvandi F (2014). U.S. Food and Drug Administration approval summary: omacetaxine mepesuccinate as treatment for chronic myeloid leukemia. Oncologist.

[266] Khoury HJ (2015). Omacetaxine mepesuccinate in patients with advanced chronic myeloid leukemia with resistance or intolerance to tyrosine kinase inhibitors. Leuk. Lymphoma.

[267] Ogbogu PU (2009). Hypereosinophilic syndrome: a multicenter, retrospective analysis of clinical characteristics and response to therapy. J. Allergy Clin. Immunol..

[268] Montemurro M (2018). Long-term outcome of dasatinib first-line treatment in gastrointestinal stromal tumor: a multicenter, 2-stage phase 2 trial (Swiss Group for Clinical Cancer Research 56/07). Cancer.

[269] Rossari F, Minutolo F, Orciuolo E (2018). Past, present, and future of Bcr-Abl inhibitors: from chemical development to clinical efficacy. J. Hematol. Oncol..

[270] Tanaka S, Baba Y (2020). B cell receptor signaling. Adv. Exp. Med. Biol..

[271] Hendriks RW, Yuvaraj S, Kil LP (2014). Targeting Bruton’s tyrosine kinase in B cell malignancies. Nat. Rev. Cancer.

[272] Schwartzberg PL, Finkelstein LD, Readinger JA (2005). TEC-family kinases: regulators of T-helper-cell differentiation. Nat. Rev. Immunol..

[273] Khan WN (1995). Defective B cell development and function in Btk-deficient mice. Immunity.

[274] Ma L (2013). Discovery and characterization of LY2784544, a small-molecule tyrosine kinase inhibitor of JAK2V617F. Blood Cancer J..

[275] Davids MS, Brown JR (2014). Ibrutinib: a first in class covalent inhibitor of Bruton’s tyrosine kinase. Future Oncol..

[276] Skarzynski M (2016). Interactions between ibrutinib and anti-CD20 antibodies: competing effects on the outcome of combination therapy. Clin. Cancer Res..

[277] Akinleye A (2013). Ibrutinib and novel BTK inhibitors in clinical development. J. Hematol. Oncol..

[278] Wang ML (2013). Targeting BTK with ibrutinib in relapsed or refractory mantle-cell lymphoma. N. Engl. J. Med..

[279] Byrd JC (2013). Targeting BTK with ibrutinib in relapsed chronic lymphocytic leukemia. N. Engl. J. Med..

[280] Noy A (2017). Targeting Bruton tyrosine kinase with ibrutinib in relapsed/refractory marginal zone lymphoma. Blood.

[281] Mato AR (2018). Toxicities and outcomes of 616 ibrutinib-treated patients in the United States: a real-world analysis. Haematologica.

[282] Rajasekaran, N. et al. Three BTK-specific inhibitors, in contrast to ibrutinib, do not antagonize rituximab-dependent NK-cell mediated cytotoxicity. *Blood*. **124**, 3118 (2014).

[283] Wu J, Zhang M, Liu D (2016). Acalabrutinib (ACP-196): a selective second-generation BTK inhibitor. J. Hematol. Oncol..

[284] Wu J, Liu C, Tsui ST, Liu D (2016). Second-generation inhibitors of Bruton tyrosine kinase. J. Hematol. Oncol..

[285] Herman SEM (2017). The Bruton tyrosine kinase (BTK) inhibitor acalabrutinib demonstrates potent on-target effects and efficacy in two mouse models of chronic lymphocytic leukemia. Clin. Cancer Res..

[286] Guo Y (2019). Discovery of zanubrutinib (BGB-3111), a novel, potent, and selective covalent inhibitor of Bruton’s tyrosine kinase. J. Med. Chem..

[287] Syed YY (2020). Zanubrutinib: first approval. Drugs.

[288] Blum KA (2015). B-cell receptor pathway modulators in NHL. Hematol. Am. Soc. Hematol. Educ. Program..

[289] Naymagon L, Abdul-Hay M (2016). Novel agents in the treatment of multiple myeloma: a review about the future. J. Hematol. Oncol..

[290] Schwartz M, Zhang Y, Rosenblatt JD (2016). B cell regulation of the anti-tumor response and role in carcinogenesis. J. Immunother. Cancer.

[291] Burger JA (2014). Bruton’s tyrosine kinase (BTK) inhibitors in clinical trials. Curr. Hematol. Malig. Rep..

[292] Walter HS (2016). A phase 1 clinical trial of the selective BTK inhibitor ONO/GS-4059 in relapsed and refractory mature B-cell malignancies. Blood.

[293] Danilov AV (2020). Phase Ib study of tirabrutinib in combination with idelalisib or entospletinib in previously treated chronic lymphocytic leukemia. Clin. Cancer Res..

[294] Evans EK (2013). Inhibition of Btk with CC-292 provides early pharmacodynamic assessment of activity in mice and humans. J. Pharm. Exp. Ther..

[295] Abdelhameed AS, Attwa MW, Al-Shaklia NS, Kadi AA (2019). A highly sensitive LC-MS/MS method to determine novel Bruton’s tyrosine kinase inhibitor spebrutinib: application to metabolic stability evaluation. R. Soc. Open Sci..

[296] Brown JR (2016). Phase I study of single-agent CC-292, a highly selective Bruton’s tyrosine kinase inhibitor, in relapsed/refractory chronic lymphocytic leukemia. Haematologica.

[297] Haselmayer P (2019). Efficacy and pharmacodynamic modeling of the BTK inhibitor evobrutinib in autoimmune disease models. J. Immunol..

[298] Watterson SH (2019). Discovery of Branebrutinib (BMS-986195): a strategy for identifying a highly potent and selective covalent inhibitor providing rapid in vivo inactivation of Bruton’s tyrosine kinase (BTK). J. Med. Chem..

[299] Park JK (2016). HM71224, a novel Bruton’s tyrosine kinase inhibitor, suppresses B cell and monocyte activation and ameliorates arthritis in a mouse model: a potential drug for rheumatoid arthritis. Arthritis Res. Ther..

[300] Zhang Z (2018). Targeting Bruton’s tyrosine kinase for the treatment of B cell associated malignancies and autoimmune diseases: preclinical and clinical developments of small molecule inhibitors. Arch. Pharm..

[301] Kim HO (2019). Development of BTK inhibitors for the treatment of B-cell malignancies. Arch. Pharm. Res..

[302] Thompson PA, Burger JA (2018). Bruton’s tyrosine kinase inhibitors: first and second generation agents for patients with chronic lymphocytic leukemia (CLL). Expert Opin. Investig. Drugs.

[303] Brandhuber B (2018). LOXO-305, a next generation reversible BTK inhibitor, for overcoming acquired resistance to irreversible BTK inhibitors. Clin. Lymphoma Myeloma Leuk..

[304] Erickson RI (2017). Bruton’s tyrosine kinase small molecule inhibitors induce a distinct pancreatic toxicity in rats. J. Pharm. Exp. Ther..

[305] Eathiraj S (2016). Targeting ibrutinib-resistant BTK-C481S mutation with ARQ 531, a reversible non-covalent inhibitor of BTK. Clin. Lymphoma Myeloma Leuk..

[306] Woyach JA (2017). BTK(C481S)-mediated resistance to ibrutinib in chronic lymphocytic leukemia. J. Clin. Oncol..

[307] Serra López-Matencio JM, Morell Baladrón A, Castañeda S (2019). JAK-STAT inhibitors for the treatment of immunomediated diseases. Med. Clín..

[308] Villarino AV, Kanno Y, O’Shea JJ (2017). Mechanisms and consequences of Jak-STAT signaling in the immune system. Nat. Immunol..

[309] Cacalano NA (1999). Autosomal SCID caused by a point mutation in the N-terminus of Jak3: mapping of the Jak3-receptor interaction domain. EMBO J..

[310] Banerjee S (2017). JAK-STAT signaling as a target for inflammatory and autoimmune diseases: current and future prospects. Drugs.

[311] O’Shea JJ (2015). The JAK-STAT pathway: impact on human disease and therapeutic intervention. Annu Rev. Med..

[312] Bryan MC, Rajapaksa NS (2018). Kinase inhibitors for the treatment of immunological disorders: recent advances. J. Med. Chem..

[313] Schwartz DM (2017). JAK inhibition as a therapeutic strategy for immune and inflammatory diseases. Nat. Rev. Drug Discov..

[314] Strand V (2015). Systematic review and meta-analysis of serious infections with tofacitinib and biologic disease-modifying antirheumatic drug treatment in rheumatoid arthritis clinical trials. Arthritis Res. Ther..

[315] Mesa RA, Yasothan U, Kirkpatrick P (2012). Ruxolitinib. Nat. Rev. Drug Discov..

[316] Jamieson C (2015). Effect of treatment with a JAK2-selective inhibitor, fedratinib, on bone marrow fibrosis in patients with myelofibrosis. J. Transl. Med..

[317] Zhang FQ (2015). JAK2 inhibitor TG101348 overcomes erlotinib-resistance in non-small cell lung carcinoma cells with mutated EGF receptor. Oncotarget.

[318] Gremese E, Ferraccioli G (2013). Tofacitinib for rheumatoid arthritis. Lancet.

[319] Sanchez GAM (2018). JAK1/2 inhibition with baricitinib in the treatment of autoinflammatory interferonopathies. J. Clin. Invest..

[320] Verstovsek S (2008). WP1066, a novel JAK2 inhibitor, suppresses proliferation and induces apoptosis in erythroid human cells carrying the JAK2 V617F mutation. Clin. Cancer Res..

[321] Hexner EO (2008). Lestaurtinib (CEP701) is a JAK2 inhibitor that suppresses JAK2/STAT5 signaling and the proliferation of primary erythroid cells from patients with myeloproliferative disorders. Blood.

[322] Mascarenhas J (2019). Phase II trial of Lestaurtinib, a JAK2 inhibitor, in patients with myelofibrosis. Leuk. Lymphoma.

[323] Mascarenhas JO (2017). Primary analysis of a phase II open-label trial of INCB039110, a selective JAK1 inhibitor, in patients with myelofibrosis. Haematologica.

[324] Yang EG (2016). Design and synthesis of Janus kinase 2 (JAK2) and histone deacetlyase (HDAC) bispecific inhibitors based on pacritinib and evidence of dual pathway inhibition in hematological cell lines. J. Med. Chem..

[325] Mesa RA (2017). Pacritinib versus best available therapy for the treatment of myelofibrosis irrespective of baseline cytopenias (PERSIST-1): an international, randomised, phase 3 trial. Lancet Haematol..

[326] Harrington R, Al Nokhatha SA, Conway R (2020). JAK inhibitors in rheumatoid arthritis: an evidence-based review on the emerging clinical data. J. Inflamm. Res..

[327] Xu P (2020). Janus kinases (JAKs): The efficient therapeutic targets for autoimmune diseases and myeloproliferative disorders. Eur. J. Med. Chem..

[328] Chan LC (2019). IL-6/JAK1 pathway drives PD-L1 Y112 phosphorylation to promote cancer immune evasion. J. Clin. Invest..

[329] Dai Z, Zeng W, Christiano AM (2018). 1098 Efficacy of selective next-generation JAK inhibitors in the treatment of alopecia areata. J. Investig. Dermatol..

[330] Norman P (2014). Selective JAK inhibitors in development for rheumatoid arthritis. Expert Opin. Investig. Drugs.

[331] Santarpia L, Lippman SM, El-Naggar AK (2012). Targeting the MAPK-RAS-RAF signaling pathway in cancer therapy. Expert Opin. Ther. Targets.

[332] Degirmenci, U., Wang, M. & Hu, J. Targeting aberrant RAS/RAF/MEK/ERK signaling for cancer therapy. *Cells*. **9**, 198 (2020). 10.3390/cells9010198 PMC7017232 31941155

[333] Flaherty KT, McArthur G (2010). BRAF, a target in melanoma: implications for solid tumor drug development. Cancer.

[334] Long GV (2011). Prognostic and clinicopathologic associations of oncogenic BRAF in metastatic melanoma. J. Clin. Oncol..

[335] Ostrem JM (2013). K-Ras(G12C) inhibitors allosterically control GTP affinity and effector interactions. Nature.

[336] Liu T, Wang Z, Guo P, Ding N (2019). Electrostatic mechanism of V600E mutation-induced B-Raf constitutive activation in colorectal cancer: molecular implications for the selectivity difference between type-I and type-II inhibitors. Eur. Biophys. J..

[337] Tsai J (2008). Discovery of a selective inhibitor of oncogenic B-Raf kinase with potent antimelanoma activity. Proc. Natl Acad. Sci. USA.

[338] Hauschild A (2012). Dabrafenib in BRAF-mutated metastatic melanoma: a multicentre, open-label, phase 3 randomised controlled trial. Lancet.

[339] Li Z (2016). Encorafenib (LGX818), a potent BRAF inhibitor, induces senescence accompanied by autophagy in BRAFV600E melanoma cells. Cancer Lett..

[340] Molnar E (2018). Pan-RAF and MEK vertical inhibition enhances therapeutic response in non-V600 BRAF mutant cells. BMC Cancer.

[341] Saro S, Diwakar D (2018). MEK inhibitors for the treatment of NRAS mutant melanoma. Drug Des. Devel. Ther.

[342] Roskoski R (2018). Targeting oncogenic Raf protein-serine/threonine kinases in human cancers. Pharm. Res..

[343] Khunger A, Khunger M, Velcheti V (2018). Dabrafenib in combination with trametinib in the treatment of patients with BRAF V600-positive advanced or metastatic non-small cell lung cancer: clinical evidence and experience. Ther. Adv. Respir. Dis..

[344] Markham A, Keam SJ (2020). Selumetinib: first approval. Drugs.

[345] Luebker SA, Koepsell SA (2019). Diverse mechanisms of BRAF inhibitor resistance in melanoma identified in clinical and preclinical studies. Front. Oncol..

[346] Al-Olabi L (2018). Mosaic RAS/MAPK variants cause sporadic vascular malformations which respond to targeted therapy. J. Clin. Invest.

[347] Arend RC (2020). EMR 20006-012: a phase II randomized double-blind placebo controlled trial comparing the combination of pimasertib (MEK inhibitor) with SAR245409 (PI3K inhibitor) to pimasertib alone in patients with previously treated unresectable borderline or low grade ovarian cancer. Gynecol. Oncol..

[348] Chenard-Poirier M (2017). Results from the biomarker-driven basket trial of RO5126766 (CH5127566), a potent RAF/MEK inhibitor, in RAS- or RAF-mutated malignancies including multiple myeloma. J. Clin. Oncol..

[349] Blake JF (2016). Discovery of (S)-1-(1-(4-Chloro-3-fluorophenyl)-2-hydroxyethyl)-4-(2-((1-methyl-1H-pyrazol-5-y l)amino)pyrimidin-4-yl)pyridin-2(1H)-one (GDC-0994), an Extracellular Signal-Regulated Kinase 1/2 (ERK1/2) Inhibitor in Early Clinical Development. J. Med. Chem..

[350] Okimoto RA (2016). Preclinical efficacy of a RAF inhibitor that evades paradoxical MAPK pathway activation in protein kinase BRAF-mutant lung cancer. Proc. Natl Acad. Sci. USA.

[351] Pelster MS, Amaria RN (2019). Combined targeted therapy and immunotherapy in melanoma: a review of the impact on the tumor microenvironment and outcomes of early clinical trials. Ther. Adv. Med. Oncol..

[352] Kakadia S (2018). Mechanisms of resistance to BRAF and MEK inhibitors and clinical update of US Food and Drug Administration-approved targeted therapy in advanced melanoma. Onco Targets Ther..

[353] Hanahan D, Weinberg RA (2011). Hallmarks of cancer: the next generation. Cell.

[354] Weinberg RA (1995). The retinoblastoma protein and cell cycle control. Cell.

[355] Hunter T, Pines J (1994). Cyclins and cancer. II: Cyclin D and CDK inhibitors come of age. Cell.

[356] O’Leary B, Finn RS, Turner NC (2016). Treating cancer with selective CDK4/6 inhibitors. Nat. Rev. Clin. Oncol..

[357] Chen P (2016). Spectrum and degree of CDK drug interactions predicts clinical performance. Mol. Cancer Ther..

[358] Anders L (2011). A systematic screen for CDK4/6 substrates links FOXM1 phosphorylation to senescence suppression in cancer cells. Cancer Cell.

[359] Asghar U, Witkiewicz AK, Turner NC, Knudsen ES (2015). The history and future of targeting cyclin-dependent kinases in cancer therapy. Nat. Rev. Drug Discov..

[360] Choi YJ (2012). The requirement for cyclin D function in tumor maintenance. Cancer Cell.

[361] Sawai CM (2012). Therapeutic targeting of the cyclin D3:CDK4/6 complex in T cell leukemia. Cancer Cell.

[362] Ameratunga M, Kipps E, Okines AFC, Lopez JS (2019). To cycle or fight-CDK4/6 inhibitors at the crossroads of anticancer immunity. Clin. Cancer Res..

[363] Chen X (2019). Latest overview of the cyclin-dependent kinases 4/6 inhibitors in breast cancer: the past, the present and the future. J. Cancer.

[364] Sanchez-Martinez C, Lallena MJ, Sanfeliciano SG, de Dios A (2019). Cyclin dependent kinase (CDK) inhibitors as anticancer drugs: recent advances (2015-2019). Bioorg. Med. Chem. Lett..

[365] Laderian B, Fojo T (2017). CDK4/6 Inhibition as a therapeutic strategy in breast cancer: palbociclib, ribociclib, and abemaciclib. Semin. Oncol..

[366] Infante JR (2016). A phase I study of the cyclin-dependent kinase 4/6 inhibitor ribociclib (LEE011) in patients with advanced solid tumors and lymphomas. Clin. Cancer Res..

[367] Patnaik A (2016). Efficacy and safety of abemaciclib, an inhibitor of CDK4 and CDK6, for patients with breast cancer, non-small cell lung cancer, and other solid tumors. Cancer Discov..

[368] Spring LM, Zangardi ML, Moy B, Bardia A (2017). Clinical management of potential toxicities and drug interactions related to cyclin-dependent kinase 4/6 inhibitors in breast cancer: practical considerations and recommendations. Oncologist.

[369] Finn RS (2015). The cyclin-dependent kinase 4/6 inhibitor palbociclib in combination with letrozole versus letrozole alone as first-line treatment of oestrogen receptor-positive, HER2-negative, advanced breast cancer (PALOMA-1/TRIO-18): a randomised phase 2 study. Lancet Oncol..

[370] Cristofanilli M (2016). Fulvestrant plus palbociclib versus fulvestrant plus placebo for treatment of hormone-receptor-positive, HER2-negative metastatic breast cancer that progressed on previous endocrine therapy (PALOMA-3): final analysis of the multicentre, double-blind, phase 3 randomised controlled trial. Lancet Oncol..

[371] Sledge GW (2017). MONARCH 2: abemaciclib in combination with fulvestrant in women with HR+/HER2- advanced breast cancer who had progressed while receiving endocrine therapy. J. Clin. Oncol..

[372] Barroso-Sousa R, Shapiro GI, Tolaney SM (2016). Clinical development of the CDK4/6 inhibitors ribociclib and abemaciclib in breast cancer. Breast Care.

[373] Goldman JW (2016). Treatment Rationale and Study Design for the JUNIPER Study: a randomized phase III study of abemaciclib with best supportive care versus erlotinib with best supportive care in patients with stage IV non-small-cell lung cancer with a detectable KRAS mutation whose disease has progressed after platinum-based chemotherapy. Clin. Lung Cancer.

[374] Tripathy D, Bardia A, Sellers WR (2017). Ribociclib (LEE011): mechanism of action and clinical impact of this selective cyclin-dependent kinase 4/6 inhibitor in various solid tumors. Clin. Cancer Res..

[375] He, S. et al. Transient CDK4/6 inhibition protects hematopoietic stem cells from chemotherapy-induced exhaustion. *Science Transl. Med.* **9**, eaal3986 (2017). 10.1126/scitranslmed.aal3986 PMC5774632 28446688

[376] Wang J (2017). Critical roles of conventional dendritic cells in promoting T cell-dependent hepatitis through regulating natural killer T cells. Clin. Exp. Immunol..

[377] Kim WS (2013). 5’-OH-5-nitro-indirubin oxime (AGM130), an indirubin derivative, induces apoptosis of Imatinib-resistant chronic myeloid leukemia cells. Leuk. Res..

[378] Wang Y (2018). Discovery of 4-((7H-pyrrolo[2,3-d]pyrimidin-4-yl)amino)-N-(4-((4-methylpiperazin-1-yl)methyl)p henyl)-1H-pyrazole-3-carboxamide (FN-1501), an FLT3- and CDK-kinase inhibitor with potentially high efficiency against acute myelocytic leukemia. J. Med Chem..

[379] Hazel P (2018). Corrigendum: inhibitor selectivity for cyclin-dependent kinase7: a structural, thermodynamic, and modelling study. ChemMedChem.

[380] Hu S (2019). Discovery and characterization of SY-1365, a selective, covalent inhibitor of CDK7. Cancer Res..

[381] Sielecki TM, Boylan JF, Benfield PA, Trainor GL (2000). Cyclin-dependent kinase inhibitors: useful targets in cell cycle regulation. J. Med. Chem..

[382] Condorelli R (2018). Polyclonal RB1 mutations and acquired resistance to CDK 4/6 inhibitors in patients with metastatic breast cancer. Ann. Oncol..

[383] Herrera-Abreu MT (2016). Early adaptation and acquired resistance to CDK4/6 inhibition in estrogen receptor-positive breast cancer. Cancer Res..

[384] Caldon CE (2012). Cyclin E2 overexpression is associated with endocrine resistance but not insensitivity to CDK2 inhibition in human breast cancer cells. Mol. Cancer Ther..

[385] Spring LM (2020). Cyclin-dependent kinase 4 and 6 inhibitors for hormone receptor-positive breast cancer: past, present, and future. Lancet.

[386] Bilanges B, Posor Y, Vanhaesebroeck B (2019). PI3K isoforms in cell signalling and vesicle trafficking. Nat. Rev. Mol. Cell Biol..

[387] Jean S, Kiger AA (2014). Classes of phosphoinositide 3-kinases at a glance. J. Cell Sci..

[388] Fruman DA (2017). The PI3K pathway in human disease. Cell.

[389] Fresno Vara JA (2004). PI3K/Akt signalling pathway and cancer. Cancer Treat. Rev..

[390] Liu N (2013). BAY 80-6946 is a highly selective intravenous PI3K inhibitor with potent p110alpha and p110delta activities in tumor cell lines and xenograft models. Mol. Cancer Ther..

[391] Vangapandu HV, Jain N, Gandhi V (2017). Duvelisib: a phosphoinositide-3 kinase delta/gamma inhibitor for chronic lymphocytic leukemia. Expert Opin. Investig. Drugs.

[392] Vangapandu HV (2017). B-cell receptor signaling regulates metabolism in chronic lymphocytic leukemia. Mol. Cancer Res..

[393] James A (2015). Absorption, distribution, metabolism, and excretion of [(14)C]BYL719 (alpelisib) in healthy male volunteers. Cancer Chemother. Pharm..

[394] Foster P (2015). The selective PI3K inhibitor XL147 (SAR245408) inhibits tumor growth and survival and potentiates the activity of chemotherapeutic agents in preclinical tumor models. Mol. Cancer Ther..

[395] Yaguchi S (2006). Antitumor activity of ZSTK474, a new phosphatidylinositol 3-kinase inhibitor. J. Natl Cancer Inst..

[396] Evans CA (2016). Discovery of a selective phosphoinositide-3-kinase (PI3K)-gamma inhibitor (IPI-549) as an immuno-oncology clinical candidate. ACS Med. Chem. Lett..

[397] Juric D (2017). A first-in-human, phase I, dose-escalation study of TAK-117, a selective PI3Kalpha isoform inhibitor, in patients with advanced solid malignancies. Clin. Cancer Res..

[398] Serra V (2008). NVP-BEZ235, a dual PI3K/mTOR inhibitor, prevents PI3K signaling and inhibits the growth of cancer cells with activating PI3K mutations. Cancer Res..

[399] Chang KY (2011). Novel phosphoinositide 3-kinase/mTOR dual inhibitor, NVP-BGT226, displays potent growth-inhibitory activity against human head and neck cancer cells in vitro and in vivo. Clin. Cancer Res..

[400] Lin J (2013). Targeting activated Akt with GDC-0068, a novel selective Akt inhibitor that is efficacious in multiple tumor models. Clin. Cancer Res..

[401] Alzahrani AS (2019). PI3K/Akt/mTOR inhibitors in cancer: At the bench and bedside. Semin. Cancer Biol..

[402] McKenna M, McGarrigle S, Pidgeon GP (2018). The next generation of PI3K-Akt-mTOR pathway inhibitors in breast cancer cohorts. Biochim. Biophys. Rev. Cancer.

[403] Schmid P (2020). Capivasertib plus paclitaxel versus placebo plus paclitaxel as first-line therapy for metastatic triple-negative breast cancer: The PAKT Trial. J. Clin. Oncol..

[404] Spencer A (2014). The novel AKT inhibitor afuresertib shows favorable safety, pharmacokinetics, and clinical activity in multiple myeloma. Blood.

[405] Tolcher AW (2020). Phase I dose-escalation trial of the oral AKT inhibitor uprosertib in combination with the oral MEK1/MEK2 inhibitor trametinib in patients with solid tumors. Cancer Chemother. Pharm..

[406] Benjamin D, Colombi M, Moroni C, Hall MN (2011). Rapamycin passes the torch: a new generation of mTOR inhibitors. Nat. Rev. Drug Discov..

[407] Li J, Kim SG, Blenis J (2014). Rapamycin: one drug, many effects. Cell Metab..

[408] Hsieh AC (2012). The translational landscape of mTOR signalling steers cancer initiation and metastasis. Nature.

[409] Basu B (2015). First-in-human pharmacokinetic and pharmacodynamic study of the dual m-TORC 1/2 inhibitor AZD2014. Clin. Cancer Res..

[410] Mosedale M (2019). Identification of candidate risk factor genes for human idelalisib toxicity using a collaborative cross approach. Toxicol. Sci..

[411] Tarakhovsky A (2010). Tools and landscapes of epigenetics. Nat. Immunol..

[412] Cheng Y (2019). Targeting epigenetic regulators for cancer therapy: mechanisms and advances in clinical trials. Signal Transduct. Target Ther..

[413] Duan R, Du W, Guo W (2020). EZH2: a novel target for cancer treatment. J. Hematol. Oncol..

[414] Yang YX (2019). Therapeutic potential of enhancer of zeste homolog 2 in autoimmune diseases. Expert Opin. Ther. Targets.

[415] Wu X (2019). Increased EZH2 expression in prostate cancer is associated with metastatic recurrence following external beam radiotherapy. Prostate.

[416] Bai Y (2019). Inhibition of enhancer of zeste homolog 2 (EZH2) overcomes enzalutamide resistance in castration-resistant prostate cancer. J. Biol. Chem..

[417] Jones BA, Varambally S, Arend RC (2018). Histone methyltransferase EZH2: A Therapeutic Target For Ovarian Cancer. Mol. Cancer Ther..

[418] Jia N (2014). Enhancer of zeste homolog 2 is involved in the proliferation of endometrial carcinoma. Oncol. Lett..

[419] Puppe J (2019). EZH2 is overexpressed in BRCA1-like breast tumors and predictive for sensitivity to high-dose platinum-based chemotherapy. Clin. Cancer Res..

[420] Mahmoud F (2016). Role of EZH2 histone methyltrasferase in melanoma progression and metastasis. Cancer Biol. Ther..

[421] Sashida G, Iwama A (2017). Multifaceted role of the polycomb-group gene EZH2 in hematological malignancies. Int J. Hematol..

[422] Italiano A (2018). Tazemetostat, an EZH2 inhibitor, in relapsed or refractory B-cell non-Hodgkin lymphoma and advanced solid tumours: a first-in-human, open-label, phase 1 study. Lancet Oncol..

[423] Lue JK, Amengual JE (2018). Emerging EZH2 inhibitors and their application in lymphoma. Curr. Hematol. Malig. Rep..

[424] Danis E (2016). Ezh2 controls an early hematopoietic program and growth and survival signaling in early T cell precursor acute lymphoblastic leukemia. Cell Rep..

[425] Miranda TB (2009). DZNep is a global histone methylation inhibitor that reactivates developmental genes not silenced by DNA methylation. Mol. Cancer Ther..

[426] Yap DB (2011). Somatic mutations at EZH2 Y641 act dominantly through a mechanism of selectively altered PRC2 catalytic activity, to increase H3K27 trimethylation. Blood.

[427] Bodor C (2013). EZH2 mutations are frequent and represent an early event in follicular lymphoma. Blood.

[428] Qi W (2017). An allosteric PRC2 inhibitor targeting the H3K27me3 binding pocket of EED. Nat. Chem. Biol..

[429] He Y (2017). The EED protein-protein interaction inhibitor A-395 inactivates the PRC2 complex. Nat. Chem. Biol..

[430] Dong H (2019). An allosteric PRC2 inhibitor targeting EED suppresses tumor progression by modulating the immune response. Cancer Res..

[431] Potjewyd F (2020). Degradation of polycomb repressive complex 2 with an EED-targeted bivalent chemical degrader. Cell Chem. Biol..

[432] Wang X (2017). A covalently bound inhibitor triggers EZH2 degradation through CHIP-mediated ubiquitination. EMBO J..

[433] Bisserier M, Wajapeyee N (2018). Mechanisms of resistance to EZH2 inhibitors in diffuse large B-cell lymphomas. Blood.

[434] Wu S (2018). SWI/SNF catalytic subunits’ switch drives resistance to EZH2 inhibitors in ARID1A-mutated cells. Nat. Commun..

[435] Huang X (2018). Targeting epigenetic crosstalk as a therapeutic strategy for EZH2-aberrant solid tumors. Cell.

[436] Gibaja V (2016). Development of secondary mutations in wild-type and mutant EZH2 alleles cooperates to confer resistance to EZH2 inhibitors. Oncogene.

[437] Tremblay-LeMay R, Rastgoo N, Pourabdollah M, Chang H (2018). EZH2 as a therapeutic target for multiple myeloma and other haematological malignancies. Biomark. Res..

[438] Zhang Y (2017). Combination of EZH2 inhibitor and BET inhibitor for treatment of diffuse intrinsic pontine glioma. Cell Biosci..

[439] Lue JK (2019). Precision targeting with EZH2 and HDAC inhibitors in epigenetically dysregulated lymphomas. Clin. Cancer Res..

[440] Richart L, Margueron R (2020). Drugging histone methyltransferases in cancer. Curr. Opin. Chem. Biol..

[441] Park SY, Kim JS (2020). A short guide to histone deacetylases including recent progress on class II enzymes. Exp. Mol. Med..

[442] Delcuve GP, Khan DH, Davie JR (2012). Roles of histone deacetylases in epigenetic regulation: emerging paradigms from studies with inhibitors. Clin. Epigenet..

[443] Li, Y. & Seto, E. HDACs and HDAC inhibitors in cancer development and therapy. *Cold Spring Harb. Perspect. Med*. **6**, a026831 (2016). 10.1101/cshperspect.a026831 PMC5046688 27599530

[444] Eckschlager, T., Plch, J., Stiborova, M. & Hrabeta, J. Histone deacetylase inhibitors as anticancer drugs. *Int. J. Mol. Sci*. **18**, 1414 (2017). 10.3390/ijms18071414 PMC5535906 28671573

[445] Dokmanovic M, Clarke C, Marks PA (2007). Histone deacetylase inhibitors: overview and perspectives. Mol. Cancer Res..

[446] Mann BS (2007). Vorinostat for treatment of cutaneous manifestations of advanced primary cutaneous T-cell lymphoma. Clin. Cancer Res..

[447] Lee HZ (2015). FDA approval: belinostat for the treatment of patients with relapsed or refractory peripheral T-cell lymphoma. Clin. Cancer Res..

[448] Yee AJ, Raje NS (2018). Panobinostat and multiple myeloma in 2018. Oncologist.

[449] Piekarz RL (2011). Phase 2 trial of romidepsin in patients with peripheral T-cell lymphoma. Blood.

[450] Xu Y, Zhang P, Liu Y (2017). Chidamide tablets: HDAC inhibition to treat lymphoma. Drugs Today.

[451] Cea M (2011). Synergistic interactions between HDAC and sirtuin inhibitors in human leukemia cells. PLoS ONE.

[452] Suraweera A, O’Byrne KJ, Richard DJ (2018). Combination therapy with histone deacetylase inhibitors (HDACi) for the treatment of cancer: achieving the full therapeutic potential of HDACi. Front. Oncol..

[453] Pan D, Lu X (2020). New therapeutic avenue of epigenetic modulations in cancer. Transl. Breast Cancer Res..

[454] Abaza YM (2017). Phase 1 dose escalation multicenter trial of pracinostat alone and in combination with azacitidine in patients with advanced hematologic malignancies. Cancer.

[455] Qin HT, Li HQ, Liu F (2017). Selective histone deacetylase small molecule inhibitors: recent progress and perspectives. Expert Opin. Ther. Pat..

[456] Kyaw MTH (2019). The HDAC inhibitor, SAHA, combined with cisplatin synergistically induces apoptosis in alpha-fetoprotein-producing hepatoid adenocarcinoma cells. Acta Histochem. Cytochem..

[457] Vitkeviciene A (2019). HDAC and HMT inhibitors in combination with conventional therapy: a novel treatment option for acute promyelocytic leukemia. J. Oncol..

[458] Gray J, Cubitt CL, Zhang S, Chiappori A (2012). Combination of HDAC and topoisomerase inhibitors in small cell lung cancer. Cancer Biol. Ther..

[459] Zhang Y (2018). Combined HDAC and bromodomain protein inhibition reprograms tumor cell metabolism and elicits synthetic lethality in glioblastoma. Clin. Cancer Res..

[460] Banik, D., Moufarrij, S. & Villagra, A. Immunoepigenetics combination therapies: an overview of the role of HDACs in cancer immunotherapy. *Int. J. Mol. Sci*. **20**, 2241 (2019). 10.3390/ijms20092241 PMC6539010 31067680

[461] Stoddard BL, Dean A, Koshland DE (1993). Structure of isocitrate dehydrogenase with isocitrate, nicotinamide adenine dinucleotide phosphate, and calcium at 2.5-A resolution: a pseudo-Michaelis ternary complex. Biochemistry.

[462] Xu X (2004). Structures of human cytosolic NADP-dependent isocitrate dehydrogenase reveal a novel self-regulatory mechanism of activity. J. Biol. Chem..

[463] Henderson NS (1965). Isozymes of isocitrate dehydrogenase: subunit structure and intracellular location. J. Exp. Zool..

[464] Yang H, Ye D, Guan KL, Xiong Y (2012). IDH1 and IDH2 mutations in tumorigenesis: mechanistic insights and clinical perspectives. Clin. Cancer Res..

[465] Amary MF (2011). IDH1 and IDH2 mutations are frequent events in central chondrosarcoma and central and periosteal chondromas but not in other mesenchymal tumours. J. Pathol..

[466] Ganguly BB, Kadam NN (2016). Mutations of myelodysplastic syndromes (MDS): an update. Mutat. Res Rev. Mutat. Res..

[467] Farshidfar F (2017). Integrative genomic analysis of cholangiocarcinoma identifies distinct IDH-mutant molecular profiles. Cell Rep..

[468] Gross S (2010). Cancer-associated metabolite 2-hydroxyglutarate accumulates in acute myelogenous leukemia with isocitrate dehydrogenase 1 and 2 mutations. J. Exp. Med..

[469] Ward PS (2010). The common feature of leukemia-associated IDH1 and IDH2 mutations is a neomorphic enzyme activity converting alpha-ketoglutarate to 2-hydroxyglutarate. Cancer Cell.

[470] Lu C (2012). IDH mutation impairs histone demethylation and results in a block to cell differentiation. Nature.

[471] Prensner JR, Chinnaiyan AM (2011). Metabolism unhinged: IDH mutations in cancer. Nat. Med..

[472] Dang L, Jin S, Su SM (2010). IDH mutations in glioma and acute myeloid leukemia. Trends Mol. Med..

[473] Kim ES (2017). Enasidenib: first global approval. Drugs.

[474] Stein EMEnasidenib (2018). a targeted inhibitor of mutant IDH2 proteins for treatment of relapsed or refractory acute myeloid leukemia. Future Oncol..

[475] Yen K (2017). AG-221, a first-in-class therapy targeting acute myeloid leukemia harboring oncogenic IDH2 mutations. Cancer Discov..

[476] Galkin M, Jonas BA (2019). Enasidenib in the treatment of relapsed/refractory acute myeloid leukemia: an evidence-based review of its place in therapy. Core Evid..

[477] Popovici-Muller J (2018). Discovery of AG-120 (Ivosidenib): a first-in-class mutant IDH1 inhibitor for the treatment of IDH1 mutant cancers. ACS Med. Chem. Lett..

[478] DiNardo CD (2018). Durable remissions with ivosidenib in IDH1-mutated relapsed or refractory AML. N. Engl. J. Med..

[479] Norsworthy KJ (2019). FDA approval summary: ivosidenib for relapsed or refractory acute myeloid leukemia with an isocitrate dehydrogenase-1 mutation. Clin. Cancer Res..

[480] Duggan S (2018). Caplacizumab: first global approval. Drugs.

[481] Feliciano P (2013). PAK inhibitor in fragile X. Nat. Genet..

[482] de Botton S (2015). Targeting isocitrate dehydrogenase (IDH)1 and IDH2 mutations clinical results in advanced hematologic malignancies. Ann. Oncol..

[483] Ma T (2018). Inhibitors of mutant isocitrate dehydrogenases 1 and 2 (mIDH1/2): an update and perspective. J. Med. Chem..

[484] Chaturvedi A (2017). Pan-mutant-IDH1 inhibitor BAY1436032 is highly effective against human IDH1 mutant acute myeloid leukemia in vivo. Leukemia.

[485] Caravella JA (2020). Structure-based design and identification of FT-2102 (olutasidenib), a potent mutant-selective IDH1 inhibitor. J. Med. Chem..

[486] Cho YS (2017). Discovery and evaluation of clinical candidate IDH305, a brain penetrant mutant IDH1 inhibitor. ACS Med. Chem. Lett..

[487] DiNardo CD, Stein EM (2018). SOHO state of the art update and next questions: IDH therapeutic targeting in AML. Clin. Lymphoma Myeloma Leuk..

[488] Harding JJ (2018). Isoform switching as a mechanism of acquired resistance to mutant isocitrate dehydrogenase inhibition. Cancer Discov..

[489] Intlekofer AM (2018). Acquired resistance to IDH inhibition through trans or cis dimer-interface mutations. Nature.

[490] Medeiros BC (2017). Isocitrate dehydrogenase mutations in myeloid malignancies. Leukemia.

[491] Fujiwara H (2018). Isocitrate dehydrogenase 1 mutation sensitizes intrahepatic cholangiocarcinoma to the BET inhibitor JQ1. Cancer Sci..

[492] DiNardo CD (2019). Venetoclax combined with decitabine or azacitidine in treatment-naive, elderly patients with acute myeloid leukemia. Blood.

[493] Chaturvedi, A. et al. Synergistic activity of IDH1 inhibitor BAY1436032 with azacitidine in IDH1 mutant acute myeloid leukemia. *Haematologica* **106**, 565–573 (2020). 10.3324/haematol.2019.236992 PMC7849562 32241846

[494] Kantarjian H (2006). Decitabine improves patient outcomes in myelodysplastic syndromes: results of a phase III randomized study. Cancer.

[495] Silverman LR (2002). Randomized controlled trial of azacitidine in patients with the myelodysplastic syndrome: a study of the cancer and leukemia group B. J. Clin. Oncol..

[496] Olino K, Park T, Ahuja N (2020). Exposing hidden targets: combining epigenetic and immunotherapy to overcome cancer resistance. Semin. Cancer Biol..

[497] Bates SE (2020). Epigenetic therapies for cancer. N. Engl. J. Med..

[498] Ghasemi S (2020). Cancer’s epigenetic drugs: where are they in the cancer medicines?. Pharmacogenomics J..

[499] Laubach JP, Moreau P, San-Miguel JF, Richardson PG (2015). Panobinostat for the treatment of multiple myeloma. Clin. Cancer Res..

[500] Knight T (2019). A delicate balance - The BCL-2 family and its role in apoptosis, oncogenesis, and cancer therapeutics. Biochem. Pharm..

[501] Warren CFA, Wong-Brown MW, Bowden NA (2019). BCL-2 family isoforms in apoptosis and cancer. Cell Death Dis..

[502] Huang K (2019). BH3-only proteins target BCL-xL/MCL-1, not BAX/BAK, to initiate apoptosis. Cell Res..

[503] Fabregat I (2009). Dysregulation of apoptosis in hepatocellular carcinoma cells. World J. Gastroenterol..

[504] Schattenberg JM, Schuchmann M, Galle PR (2011). Cell death and hepatocarcinogenesis: dysregulation of apoptosis signaling pathways. J. Gastroenterol. Hepatol..

[505] Perini GF (2018). BCL-2 as therapeutic target for hematological malignancies. J. Hematol. Oncol..

[506] Ashkenazi A, Fairbrother WJ, Leverson JD, Souers AJ (2017). From basic apoptosis discoveries to advanced selective BCL-2 family inhibitors. Nat. Rev. Drug Discov..

[507] Zhu Y (2016). Identification of a novel senolytic agent, navitoclax, targeting the Bcl-2 family of anti-apoptotic factors. Aging Cell.

[508] Lampson BL, Davids MS (2017). The development and current use of BCL-2 inhibitors for the treatment of chronic lymphocytic leukemia. Curr. Hematol. Malig. Rep..

[509] Anderson MA, Huang D, Roberts A (2014). Targeting BCL2 for the treatment of lymphoid malignancies. Semin. Hematol..

[510] Valentin R, Grabow S, Davids MS (2018). The rise of apoptosis: targeting apoptosis in hematologic malignancies. Blood.

[511] Tse C (2008). ABT-263: a potent and orally bioavailable Bcl-2 family inhibitor. Cancer Res..

[512] Stilgenbauer S (2016). Venetoclax in relapsed or refractory chronic lymphocytic leukaemia with 17p deletion: a multicentre, open-label, phase 2 study. Lancet Oncol..

[513] Deeks ED (2016). Venetoclax: first global approval. Drugs.

[514] Guerra VA, DiNardo C, Konopleva M (2019). Venetoclax-based therapies for acute myeloid leukemia. Best Pr. Res. Clin. Haematol..

[515] Caenepeel S (2018). AMG 176, a selective MCL1 inhibitor, is effective in hematologic cancer models alone and in combination with established therapies. Cancer Discov..

[516] Casara P (2018). S55746 is a novel orally active BCL-2 selective and potent inhibitor that impairs hematological tumor growth. Oncotarget.

[517] McBride A (2019). The role of inhibition of apoptosis in acute leukemias and myelodysplastic syndrome. Front. Oncol..

[518] Tron AE (2018). Discovery of Mcl-1-specific inhibitor AZD5991 and preclinical activity in multiple myeloma and acute myeloid leukemia. Nat. Commun..

[519] Yalniz FF, Wierda WG (2019). Targeting BCL2 in chronic lymphocytic leukemia and other hematologic malignancies. Drugs.

[520] Yang S (2019). The chemical biology of apoptosis: revisited after 17 years. Eur. J. Med. Chem..

[521] Aldoss I (2019). Venetoclax and hypomethylating agents in TP53-mutated acute myeloid leukaemia. Br. J. Haematol..

[522] Villalobos-Ortiz M (2020). BH3 profiling discriminates on-target small molecule BH3 mimetics from putative mimetics. Cell Death Differ..

[523] Thomas S (2013). Targeting the Bcl-2 family for cancer therapy. Expert Opin. Ther. Targets.

[524] Tahir SK (2017). Potential mechanisms of resistance to venetoclax and strategies to circumvent it. BMC Cancer.

[525] Lok SW (2019). A phase Ib dose-escalation and expansion study of the BCL2 inhibitor venetoclax combined with tamoxifen in ER and BCL2-positive metastatic breast cancer. Cancer Discov..

[526] Pak E, Segal RA (2016). Hedgehog signal transduction: key players, oncogenic drivers, and cancer therapy. Dev. Cell.

[527] Tukachinsky H, Petrov K, Watanabe M, Salic A (2016). Mechanism of inhibition of the tumor suppressor Patched by Sonic Hedgehog. Proc. Natl Acad. Sci. USA.

[528] Dlugosz A, Agrawal S, Kirkpatrick P (2012). Vismodegib. Nat. Rev. Drug Discov..

[529] Jain S, Song R, Xie J (2017). Sonidegib: mechanism of action, pharmacology, and clinical utility for advanced basal cell carcinomas. Onco Targets Ther..

[530] Basset-Seguin N (2017). Vismodegib in patients with advanced basal cell carcinoma: Primary analysis of STEVIE, an international, open-label trial. Eur. J. Cancer.

[531] Lear JT (2018). Long-term efficacy and safety of sonidegib in patients with locally advanced and metastatic basal cell carcinoma: 30-month analysis of the randomized phase 2 BOLT study. J. Eur. Acad. Dermatol Venereol..

[532] Sekulic A (2012). Efficacy and safety of vismodegib in advanced basal-cell carcinoma. N. Engl. J. Med..

[533] LoRusso PM (2011). Phase I trial of hedgehog pathway inhibitor vismodegib (GDC-0449) in patients with refractory, locally advanced or metastatic solid tumors. Clin. Cancer Res..

[534] Stathis A (2017). Phase I trial of the oral smoothened inhibitor sonidegib in combination with paclitaxel in patients with advanced solid tumors. Invest. N. Drugs.

[535] Li Y, Song Q, Day BW, Phase I (2019). and phase II sonidegib and vismodegib clinical trials for the treatment of paediatric and adult MB patients: a systemic review and meta-analysis. Acta Neuropathol. Commun..

[536] Jamieson C, Martinelli G, Papayannidis C, Cortes JE (2020). Hedgehog pathway inhibitors: a new therapeutic class for the treatment of acute myeloid leukemia. Blood Cancer Discov..

[537] Xie, H., Paradise, B. D., Ma, W. W. & Fernandez-Zapico, M. E. Recent advances in the clinical targeting of Hedgehog/GLI signaling in cancer. *Cells*. **8**, 394 (2019). 10.3390/cells8050394 PMC6562674 31035664

[538] Pietrobono, S. & Stecca, B. Targeting the oncoprotein smoothened by small molecules: focus on novel acylguanidine derivatives as potent smoothened inhibitors. *Cells* **7**, 272 (2018). 10.3390/cells7120272 PMC6316656 30558232

[539] Suzman DL, Antonarakis ES (2015). Clinical implications of hedgehog pathway signaling in prostate cancer. Cancers.

[540] Cortes JE, Gutzmer R, Kieran MW, Solomon JA (2019). Hedgehog signaling inhibitors in solid and hematological cancers. Cancer Treat. Rev..

[541] Girardi, D., Barrichello, A., Fernandes, G. & Pereira, A. Targeting the Hedgehog pathway in cancer: current evidence and future perspectives. *Cells* **8**, 153 (2019). 10.3390/cells8020153 PMC6406365 30759860

[542] Bhateja, P., Cherian, M., Majumder, S. & Ramaswamy, B. The Hedgehog signaling pathway: a viable target in breast cancer? *Cancers* **11**, 1126 (2019). 10.3390/cancers11081126 PMC6721501 31394751

[543] Kim J (2010). Arsenic antagonizes the Hedgehog pathway by preventing ciliary accumulation and reducing stability of the Gli2 transcriptional effector. Proc. Natl Acad. Sci. USA.

[544] Liu, X. et al. Development of hedgehog pathway inhibitors by epigenetically targeting GLI through BET bromodomain for the treatment of medulloblastoma. *Acta Pharm. Sin. B* **11**, 488–504 (2020). 10.1016/j.apsb.2020.07.007 PMC7893122 33643826

[545] Tu J (2018). Molecular modeling study on resistance of WT/D473H SMO to antagonists LDE-225 and LEQ-506. Pharm. Res..

[546] Ueno H (2018). A phase I and pharmacokinetic study of taladegib, a Smoothened inhibitor, in Japanese patients with advanced solid tumors. Invest. N. Drugs.

[547] Dong X, Wang C, Chen Z, Zhao W (2018). Overcoming the resistance mechanisms of smoothened inhibitors. Drug Discov. Today.

[548] Adams J (2003). The proteasome: structure, function, and role in the cell. Cancer Treat. Rev..

[549] Burger AM, Seth AK (2004). The ubiquitin-mediated protein degradation pathway in cancer: therapeutic implications. Eur. J. Cancer.

[550] Kodroń, A., Mussulini, B. H., Pilecka, I. & Chacińska, A. The ubiquitin-proteasome system and its crosstalk with mitochondria as therapeutic targets in medicine. *Pharmacol Res.* **163**, 105248 (2020). 10.1016/j.phrs.2020.105248 33065283

[551] Adams J (2004). The proteasome: a suitable antineoplastic target. Nat. Rev. Cancer.

[552] Gandolfi S (2017). The proteasome and proteasome inhibitors in multiple myeloma. Cancer Metastasis Rev..

[553] Adams J, Kauffman M (2004). Development of the proteasome inhibitor Velcade (Bortezomib). Cancer Investig..

[554] Fricker LD (2020). Proteasome inhibitor drugs. Annu. Rev. Pharmacol. Toxicol..

[555] Dimopoulos MA, Richardson PG, Moreau P, Anderson KC (2015). Current treatment landscape for relapsed and/or refractory multiple myeloma. Nat. Rev. Clin. Oncol..

[556] Dick LR, Fleming PE (2010). Building on bortezomib: second-generation proteasome inhibitors as anti-cancer therapy. Drug Discov. Today.

[557] Herndon TM (2013). U.s. Food and Drug Administration approval: carfilzomib for the treatment of multiple myeloma. Clin. Cancer Res..

[558] Demo SD (2007). Antitumor activity of PR-171, a novel irreversible inhibitor of the proteasome. Cancer Res..

[559] Zanwar S, Abeykoon JP, Kapoor P (2018). Ixazomib: a novel drug for multiple myeloma. Expert Rev. Hematol..

[560] Kupperman E (2010). Evaluation of the proteasome inhibitor MLN9708 in preclinical models of human cancer. Cancer Res..

[561] Xie J (2019). Ixazomib - the first oral proteasome inhibitor. Leuk. Lymphoma.

[562] Groll M, Huber R, Potts BC (2006). Crystal structures of Salinosporamide A (NPI-0052) and B (NPI-0047) in complex with the 20S proteasome reveal important consequences of beta-lactone ring opening and a mechanism for irreversible binding. J. Am. Chem. Soc..

[563] Ma L, Diao A (2015). Marizomib, a potent second generation proteasome inhibitor from natural origin. Anti-Cancer Agents Med. Chem..

[564] Zhou HJ (2009). Design and synthesis of an orally bioavailable and selective peptide epoxyketone proteasome inhibitor (PR-047). J. Med. Chem..

[565] Piva R (2008). CEP-18770: a novel, orally active proteasome inhibitor with a tumor-selective pharmacologic profile competitive with bortezomib. Blood.

[566] Gallerani E (2013). A first in human phase I study of the proteasome inhibitor CEP-18770 in patients with advanced solid tumours and multiple myeloma. Eur. J. Cancer.

[567] Vogl DT (2017). Phase I/II study of the novel proteasome inhibitor delanzomib (CEP-18770) for relapsed and refractory multiple myeloma. Leuk. Lymphoma.

[568] Park JE (2018). Next-generation proteasome inhibitors for cancer therapy. Transl. Res..

[569] Dolloff NGEmerging (2015). Therapeutic strategies for overcoming proteasome inhibitor resistance. Adv. Cancer Res..

[570] Caldecott KW (2014). Protein ADP-ribosylation and the cellular response to DNA strand breaks. DNA Repair.

[571] Vyas S, Chang P (2014). New PARP targets for cancer therapy. Nat. Rev. Cancer.

[572] Dobzhansky T (1946). Genetics of natural populations; recombination and variability in populations of Drosophila pseudoobscura. Genetics.

[573] King MC, Marks JH, Mandell JB, New York Breast Cancer Study, Group. (2003). Breast and ovarian cancer risks due to inherited mutations in BRCA1 and BRCA2. Science.

[574] Lord CJ, Ashworth A (2017). PARP inhibitors: synthetic lethality in the clinic. Science.

[575] Kim G (2015). FDA approval summary: olaparib monotherapy in patients with deleterious germline BRCA-mutated advanced ovarian cancer treated with three or more lines of chemotherapy. Clin. Cancer Res..

[576] Dockery LE, Gunderson CC, Moore KN (2017). Rucaparib: the past, present, and future of a newly approved PARP inhibitor for ovarian cancer. Onco Targets Ther..

[577] Essel KG, Moore KN (2018). Niraparib for the treatment of ovarian cancer. Expert Rev. Anticancer Ther..

[578] Hoy SM (2018). Talazoparib: first global approval. Drugs.

[579] Strom CE (2011). Poly (ADP-ribose) polymerase (PARP) is not involved in base excision repair but PARP inhibition traps a single-strand intermediate. Nucleic Acids Res..

[580] Pujade-Lauraine E (2017). Olaparib tablets as maintenance therapy in patients with platinum-sensitive, relapsed ovarian cancer and a BRCA1/2 mutation (SOLO2/ENGOT-Ov21): a double-blind, randomised, placebo-controlled, phase 3 trial. Lancet Oncol..

[581] Konstantinopoulos, P. A. et al. Single-Arm phases 1 and 2 trial of niraparib in combination with pembrolizumab in patients with recurrent platinum-resistant ovarian carcinoma. *JAMA Oncol*. **5**, 1141–1149 (2019). 10.1001/jamaoncol.2019.1048 PMC6567832 31194228

[582] Sachdev E (2019). PARP inhibition in cancer: an update on clinical development. Target Oncol..

[583] Mateo J (2019). A decade of clinical development of PARP inhibitors in perspective. Ann. Oncol..

[584] Donawho CK (2007). ABT-888, an orally active poly(ADP-ribose) polymerase inhibitor that potentiates DNA-damaging agents in preclinical tumor models. Clin. Cancer Res..

[585] Coleman RL (2019). Veliparib with first-line chemotherapy and as maintenance therapy in ovarian cancer. N. Engl. J. Med..

[586] Moore K (2018). Maintenance olaparib in patients with newly diagnosed advanced ovarian cancer. N. Engl. J. Med..

[587] Jiang X (2019). PARP inhibitors in ovarian cancer: Sensitivity prediction and resistance mechanisms. J. Cell Mol. Med..

[588] D’Andrea AD (2018). Mechanisms of PARP inhibitor sensitivity and resistance. DNA Repair.

[589] Haynes B, Murai J, Lee JM (2018). Restored replication fork stabilization, a mechanism of PARP inhibitor resistance, can be overcome by cell cycle checkpoint inhibition. Cancer Treat. Rev..

[590] Carrassa L, Colombo I, Damia G, Bertoni F (2020). Targeting the DNA damage response for patients with lymphoma: Preclinical and clinical evidences. Cancer Treat. Rev..

[591] Burgess, B. T. et al. Olaparib combined with an ATR or Chk1 inhibitor as a treatment strategy for acquired olaparib-resistant BRCA1 mutant ovarian cells. *Diagnostics* **10**, 121 (2020). 10.3390/diagnostics10020121 PMC7168282 32098452

[592] Awada A (2016). An open-label, dose-escalation study to evaluate the safety and pharmacokinetics of CEP-9722 (a PARP-1 and PARP-2 inhibitor) in combination with gemcitabine and cisplatin in patients with advanced solid tumors. Anticancer Drugs.

[593] Schram AM, Chang MT, Jonsson P, Drilon A (2017). Fusions in solid tumours: diagnostic strategies, targeted therapy, and acquired resistance. Nat. Rev. Clin. Oncol..

[594] Pottier, C. et al. Tyrosine kinase inhibitors in cancer: breakthrough and challenges of targeted therapy. *Cancers* **12**, 731 (2020). 10.3390/cancers12030731 PMC7140093 32244867

[595] Gasch C, Ffrench B, O’Leary JJ, Gallagher MF (2017). Catching moving targets: cancer stem cell hierarchies, therapy-resistance & considerations for clinical intervention. Mol. Cancer.

[596] Najafi M, Mortezaee K, Majidpoor J (2019). Cancer stem cell (CSC) resistance drivers. Life Sci..

[597] Erin N, Grahovac J, Brozovic A, Efferth T (2020). Tumor microenvironment and epithelial mesenchymal transition as targets to overcome tumor multidrug resistance. Drug Resist. Updat..

[598] Bukowski K, Kciuk M, Kontek R (2020). Mechanisms of multidrug resistance in cancer chemotherapy. Int. J. Mol. Sci..

[599] Mele L (2020). The role of autophagy in resistance to targeted therapies. Cancer Treat. Rev..

[600] Hussain S (2019). Cancer drug resistance: a fleet to conquer. J. Cell Biochem..

[601] Boumahdi S, de Sauvage FJ (2020). The great escape: tumour cell plasticity in resistance to targeted therapy. Nat. Rev. Drug Discov..

[602] Shi H, Wei J, He C (2019). Where, when, and how: context-dependent functions of RNA methylation writers, readers, and erasers. Mol. Cell.

[603] Meyer KD, Jaffrey SR (2017). Rethinking m(6)A readers, writers, and erasers. Annu Rev. Cell Dev. Biol..

[604] Van Meter EN, Onyango JA, Teske KA (2020). A review of currently identified small molecule modulators of microRNA function. Eur. J. Med. Chem..

[605] Pylayeva-Gupta Y, Grabocka E, Bar-Sagi D (2011). RAS oncogenes: weaving a tumorigenic web. Nat. Rev. Cancer.

[606] Jancik S, Drabek J, Radzioch D, Hajduch M (2010). Clinical relevance of KRAS in human cancers. J. Biomed. Biotechnol..

[607] Cox AD (2014). Drugging the undruggable RAS: mission possible?. Nat. Rev. Drug Discov..

[608] Cox AD, Der CJ, Philips MR (2015). Targeting RAS membrane association: back to the future for anti-RAS drug discovery?. Clin. Cancer Res..

[609] Papke B, Der CJ (2017). Drugging RAS: know the enemy. Science.

[610] Chen H (2020). Small-molecule inhibitors directly targeting KRAS as anticancer therapeutics. J. Med. Chem..

[611] Nagasaka M (2020). KRAS G12C Game of Thrones, which direct KRAS inhibitor will claim the iron throne?. Cancer Treat. Rev..

[612] Liu P, Wang Y, Li X (2019). Targeting the untargetable KRAS in cancer therapy. Acta Pharm. Sin. B.

[613] Bayliss R, Burgess SG, Leen E, Richards MW (2017). A moving target: structure and disorder in pursuit of Myc inhibitors. Biochem. Soc. Trans..

[614] Krzyzosiak A (2018). Target-based discovery of an inhibitor of the regulatory phosphatase PPP1R15B. Cell.

[615] Kieffer C, Jourdan JP, Jouanne M, Voisin-Chiret AS (2020). Noncellular screening for the discovery of protein-protein interaction modulators. Drug Discov. Today.

[616] Mabonga L, Kappo AP (2019). Protein-protein interaction modulators: advances, successes and remaining challenges. Biophys. Rev..

[617] Taylor MH (2020). Phase IB/II trial of lenvatinib plus pembrolizumab in patients with advanced renal cell carcinoma, endometrial cancer, and other selected advanced solid tumors. J. Clin. Oncol..

[618] Chau V, Bilusic M (2020). Pembrolizumab in combination with axitinib as first-line treatment for patients with renal cell carcinoma (RCC): evidence to date. Cancer Manag. Res..

[619] Rini BI (2019). Pembrolizumab plus axitinib versus sunitinib for advanced renal-cell carcinoma. N. Engl. J. Med..

[620] Schapira L (2011). Simple rules can improve prognostic accuracy. J. Clin. Oncol..

[621] Beck A (2010). The next generation of antibody-drug conjugates comes of age. Discov. Med..

[622] Thomas A, Teicher BA, Hassan R (2016). Antibody–drug conjugates for cancer therapy. Lancet Oncol..

[623] Yaghoubi S (2020). Potential drugs used in the antibody-drug conjugate (ADC) architecture for cancer therapy. J. Cell Physiol..

[624] Torre, B. G. & Albericio, F. An analysis of FDA drug approvals from the perspective of molecules. *Molecules* **26**, 627 (2021). 10.3390/molecules26030627 PMC7865374 33504104

[625] An S, Fu L (2018). Small-molecule PROTACs: an emerging and promising approach for the development of targeted therapy drugs. EBioMedicine.

[626] Wang Y (2020). Degradation of proteins by PROTACs and other strategies. Acta Pharm. Sin. B.

[627] Lin X, Xiang H, Luo G (2020). Targeting estrogen receptor alpha for degradation with PROTACs: A promising approach to overcome endocrine resistance. Eur. J. Med. Chem..

[628] Huang A, Garraway LA, Ashworth A, Weber B (2020). Synthetic lethality as an engine for cancer drug target discovery. Nat. Rev. Drug Discov..

[629] Corcoran RB (2013). Synthetic lethal interaction of combined BCL-XL and MEK inhibition promotes tumor regressions in KRAS mutant cancer models. Cancer Cell.

