# Supplementary material for: Small molecules in targeted cancer therapy: advances, challenges, and future perspectives
Source: Signal Transduct Target Ther. 2021 May 31;6:201. doi: 10.1038/s41392-021-00572-w (PMC8165101; doi:10.1038/s41392-021-00572-w)

**Small Molecules in Targeted Cancer Therapy: Advances, Challenges, and Future Perspectives**

Lei Zhong^1, 2^, Yueshan Li^1^, Liang Xiong^1^, Wenjing Wang^1^, Ming Wu^1^, Ting Yuan^2^, Wei Yang^1^, Chenyu Tian^1^, Zhuang Miao^1^, Tianqi Wang^1^, and Shengyong Yang^1^

^1^State Key Laboratory of Biotherapy and Cancer Center, West China Hospital, Sichuan University, Chengdu, Sichuan 610041, P.R. China.

^2^Personalized Drug Therapy Key Laboratory of Sichuan Province, Department of Pharmacy, Sichuan Provincial People’s Hospital, School of Medicine, University of Electronic Science and Technology of China, Chengdu, Sichuan 610072, P.R. China.

These authors contributed equally: Lei Zhong, Yueshan Li, Liang Xiong and Wenjing Wang.

Corresponding authors: Shengyong Yang, State Key Laboratory of Biotherapy and Cancer Center, West China Hospital, Sichuan University, Chengdu, Sichuan 610041, China. E-mail: [yangsy@scu.edu.cn](mailto:yangsy@scu.edu.cn)

**Supplementary materials：**

**Supplementary Table S1-S5**

**Supplementary Figure S1**

**Table S1 Features of small molecule and macromolecule drugs.**

| **Comparison items** | **Small molecules** | **Macromolecules** |
| --- | --- | --- |
| molecular weight | < 1000 Da | 1500-150000 Da |
| off-target effect | more common | rarer |
| penetrate cell membrane | easy | difficult |
| penetrate the blood brain barrier | relatively easy | very difficult |
| administration method | mainly oral | injection |
| method of drug absorption | mainly simple diffusion | mainly active transfer |
| the main difficulty of development | long time period | technically difficult |
| current main category | signal transduction inhibitors | vaccines, proteins, antibodies, nucleic acids |

**Table S2 Small molecule kinase inhibitors evaluated in phase III trials for cancers**

| **Chemical structure** | **Name** | | **Targets** | | **Indications (start year)** | **Corporation** |
| --- | --- | --- | --- | --- | --- | --- |
|  | Pamufetinib | | c-Met/VEGFR-2 | | Osteosarcoma (2020) | Taiho |
|  | Alflutinib | | EGFR | | NSCLC (2019) | Allist Pharmaceutical |
|  | Mobocertinib | | EGFR | | NSCLC (2019) | Takeda |
|  | Famitinib | | FLT3/c-Kit/PDGFR-β/VEGFR-1/2/3 | | CRC (2015)/NSCLC (2016)/GIST (2020) | Hengrui |
|  | Crenolanib | | FLT3/PDGFR-α | | GIST (2016)/AML (2018) | Arog Pharmaceuticals |
|  | Dovitinib | | FLT3/EGFR/FGFR-1/3/PDGFR-β/VEGFR-1/2 | | RCC (2014) | Allarity Therapeutics |
|  | Cediranib | VEGFR-1/2/3 | | Ovarian cancer (2015) | | AstraZeneca |
| 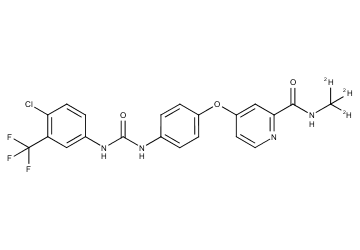 | Donafenib | VEGFR/Raf-1/BRAF/PDGFR | | HCC (2016) | | Protia |
|  | Sitravatinib | PDGFRα/β/VEGFR-1/2/3/DDR2/EPH/FLT3/c-Met/c-Kit/RET/ /MERTK/AXL | | NSCLC (2019) | | Mirati Therapeutics |
|  | Infigratinib | FGFR1/2/3/4 | | Bladder cancer (2020) | | QED Therapeutics |
|  | Asciminib | Bcr-Abl | | CML (2017) | | Novartis |
|  | Momelotinib | JAK1/2/ALK2 | | Myelofibrosis（2019） | | Sierra Oncology |
|  | Ipatasertib | AKT1/2/3 | | Breast cancer (2019) | | Roche/ Chugai Pharmaceutical |
|  | Capivasertib | AKT1/2/3/p70S6K | | TNBC (2019)/Breast cancer (2020)/Prostate cancer (2020) | | AstraZeneca |
|  | Parsaclisib | PI3Kδ | | Myelofibrosis (2020) | | Incyte |
|  | Inavolisib | PI3Kα | | Breast cancer (2020) | | Roche |
|  | Buparlisib | PI3Kα/β/δ/γ | | Head and neck squamous cell carcinoma (2020) | | Adlai Nortye |
|  | Enzastaurin | PI3K/AKT/PKC-β | | Glioblastoma multiforme (2021)/DLBCL (2018) | | Denovo Biopharma |
| 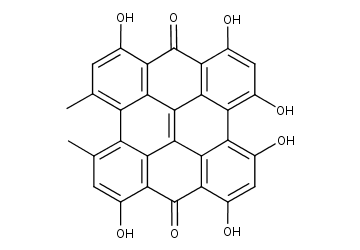 | Hypericin | PKC | | CTCL (2015) | | Soligenix |
| 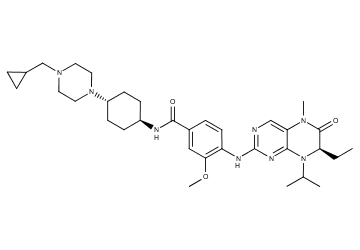 | Volasertib | PLK1 | | AML (2013) | | Boehringer Ingelheim |

**Table S3 Small molecule epigenetic inhibitors evaluated in phase III trials for cancers**

| **Chemical structure** | **Name** | **Targets** | **Indications (start year)** | **Corporation** |
| --- | --- | --- | --- | --- |
| 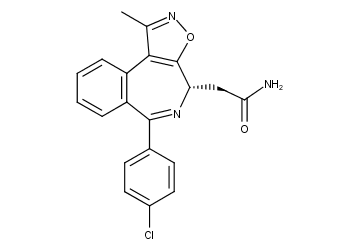 | Pelabresib | BRD4 | Myelofibrosis (2020) | Constellation Pharmaceuticals |
|  | Abexinostat | HDACs/RAD51 | Renal cell carcinoma (2019) | Xynomic Pharmaceuticals |
|  | Givinostat | HDACs/IL-1β/TNF-α | Duchenne muscular dystrophy (2017) | Italfarmaco |
| 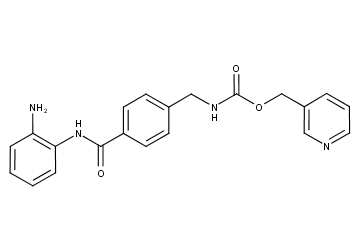 | Inavolisib | HDAC1/2 | Breast cancer (2018) | Bayer |
|  | Vorasidenib | IDH1/2 | Glioma (2019) | Agios Pharmaceuticals |

**Table S4 Small molecule inhibitors of apoptosis inducers, hedgehog pathway, proteasome, and PARP evaluated in phase III trials for cancers**

| **Chemical structure** | **Name** | **Targets** | **Indications (start year)** | **Corporation** |
| --- | --- | --- | --- | --- |
|  | Navitoclax | BCL-2/BCL-X/BCL-W/BFL-1/MDM2 | Myelofibrosis (2020) | AbbVie |
|  | Patidegib | SMO | Basal cell nevus syndrome (2019) | PellePharm |
|  | Salinosporamide A | Proteasome | Glioblastoma multiforme (2018) | Celgene |
|  | Senaparib | PARP1 | Ovarian cancer (2019) | IMPACT Therapeutics |
|  | Fluzoparib | PARP1/2 | Ovarian cancer (2019)/ Adenocarcinoma (2020) | Hengrui |
|  | Veliparib | PARP1/2 | NSCLC (2014)/ Breast cancer (2014)/ Ovarian cancer (2015)/ Fallopian tube cancer (2015) | AbbVie |

**Table S5 Small molecule inhibitors of other targets evaluated in phase III trials for cancers**

| **Chemical structure** | **Name** | **Targets** | **Indications (start year)** | **Corporation** |
| --- | --- | --- | --- | --- |
| 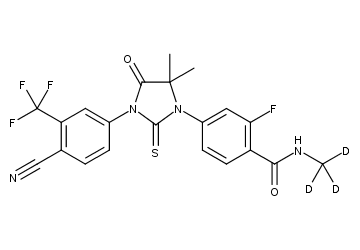 | HC-1119 | Androgen Receptor (AR) | Prostate cancer (2019) | Hinova Pharmaceuticals |
| 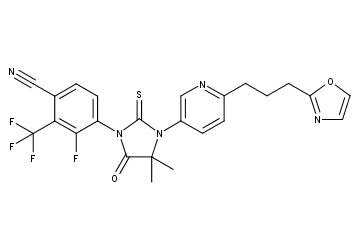 | Proxalutamide | Androgen Receptor (AR) | Prostate cancer (2018) | Kintor Pharmaceuticals |
|  | Nitroxoline | Cathepsin B/  MetAP2/SIRT1 | Bladder cancer (2017) | Asieris Pharmaceuticals |
| 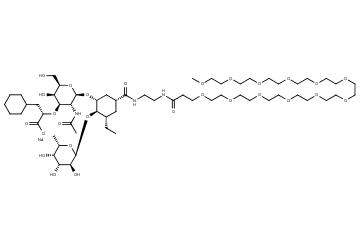 | Uproleselan sodium | E-Selectin | AML (2018) | GlycoMimetics |
| 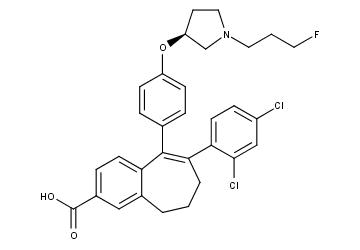 | Amcenestrant | Estrogen Receptor (ER) | Breast cancer (2020) | Sanofi |
| 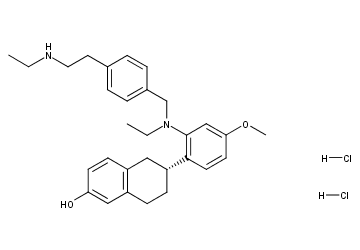 | Elacestrant | Estrogen Receptor (ER) | Breast cancer (2018) | Eisai |
| 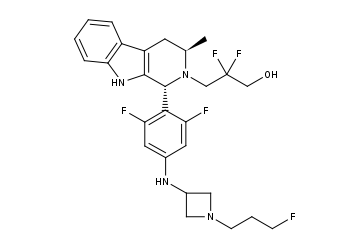 | Giredestrant | Estrogen Receptor (ER)/PKC | Breast cancer (2020) | Roche |
| 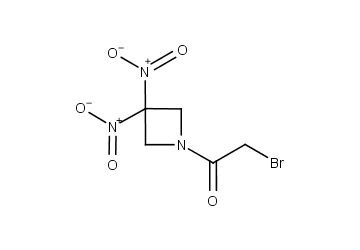 | RRx-001 | G6PD | SCLC (2018) | EpicentRx |
| 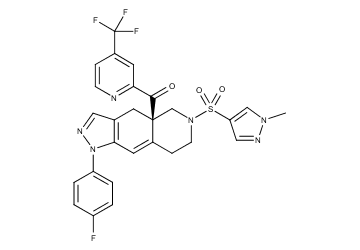 | Relacorilant | Glucocorticoid Receptor (GR) | Pancreatic ductal adenocarcinoma (2020) | Corcept Therapeutics |
| 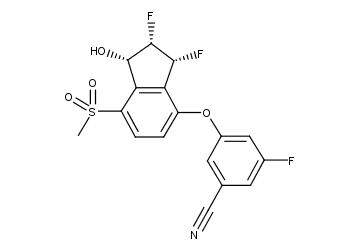 | Belzutifan | HIF-2α | Renal carcinoma | Merck & Co. |
| 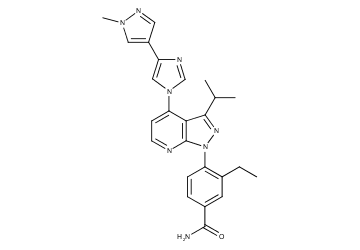 | Pimitespib | HSP90 | GIST (2018) | Taiho |
| 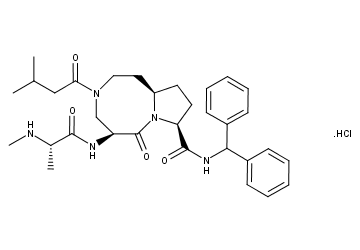 | Xevinapant hydrochloride | IAP1/2/3 | Head and neck squamous cell carcinoma (2020) | Ascentage Pharma |
| 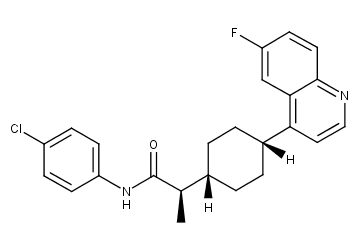 | Linrodostat | IDO1 | Melanoma (2017)/ Head and neck squamous cell carcinoma (2018)/ Bladder cancer (2018) | Bristol-Myers Squibb |
| 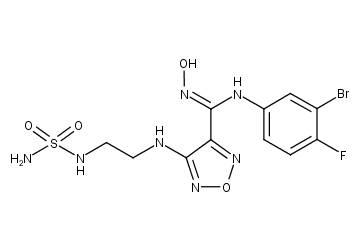 | Epacadostat | IDO1 | NSCLC (2017) | Incyte |
| 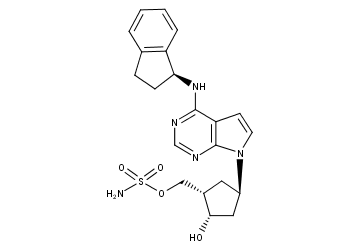 | Pevonedistat | NAE1 | AML (2019) | Takeda |
| 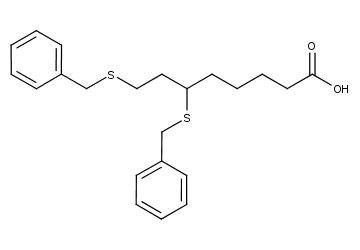 | Devimistat | PDH/OGDH | AML (2018) | Rafael Pharmaceuticals |
| 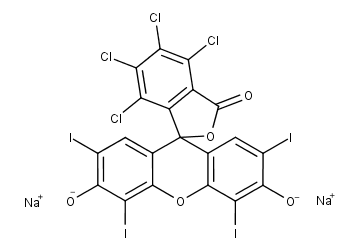 | Rose Bengal disodium | Preprotein Translocase SecA | Melanoma (2015) | Provectus Biopharmaceuticals |
| 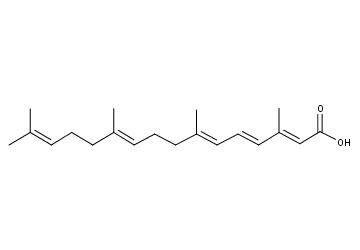 | Acyclic retinoid | RXRα/RARβ | HCC (2012) | Eisai |
| 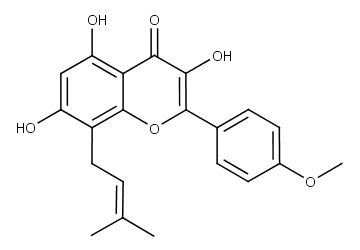 | Anhydroicaritin | SREBP/SPK1/FAS | HCC (2017) | Shenogen |
| 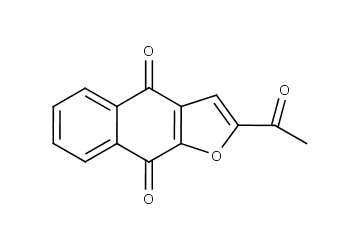 | Napabucasin | STAT3 | Pancreatic adenocarcinoma (2017) | Sumitomo Dainippon Pharma |
| 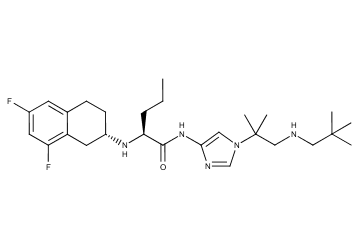 | Nirogacestat | γ-Secretase | Desmoid tumors (2019) | Pfizer |

**Supplementary Figure Legend:**

**Supplementary Figure S1:** Overview for the clinical trials of major small molecule targeted anti-cancer drugs and the targets involved.


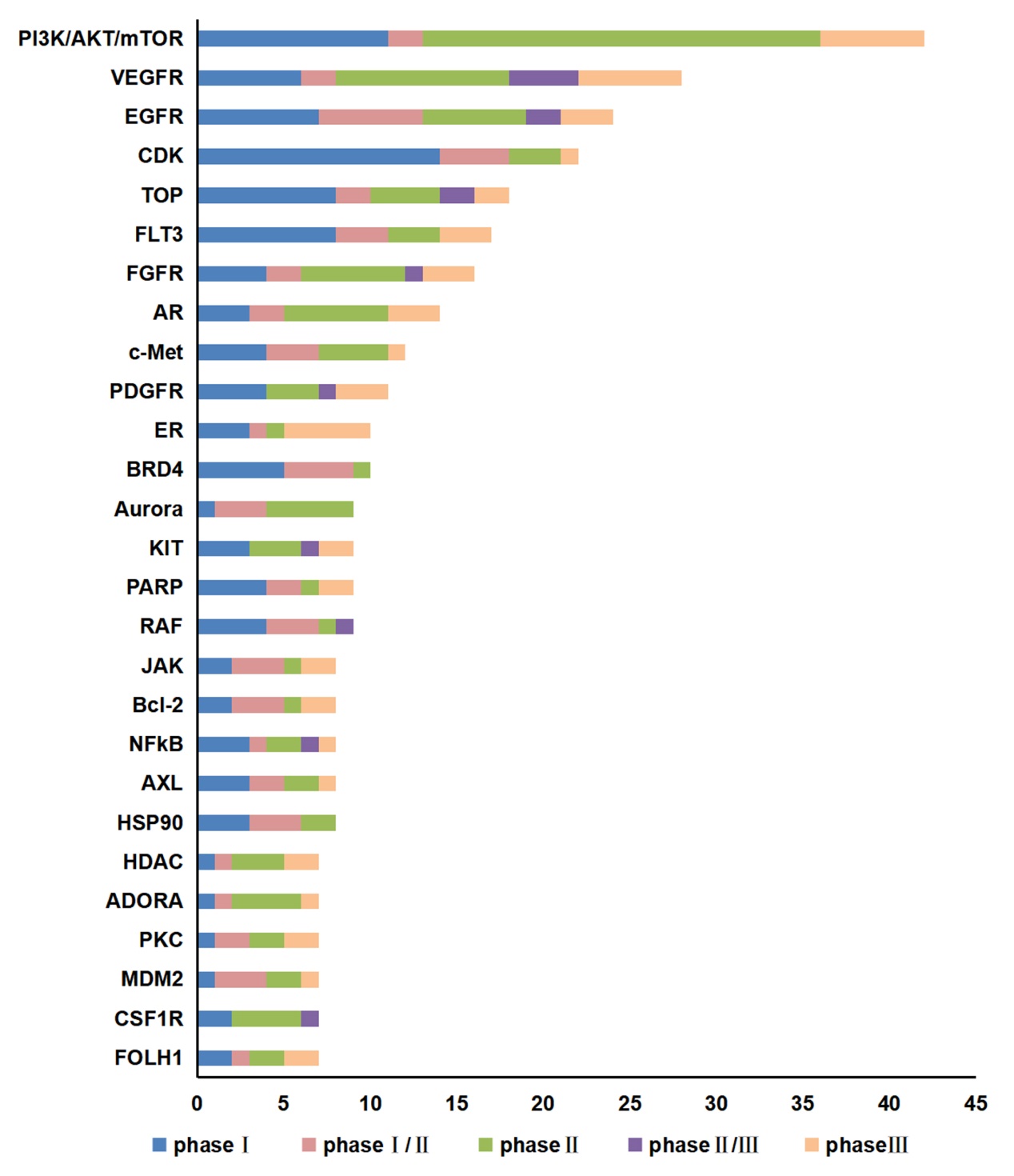


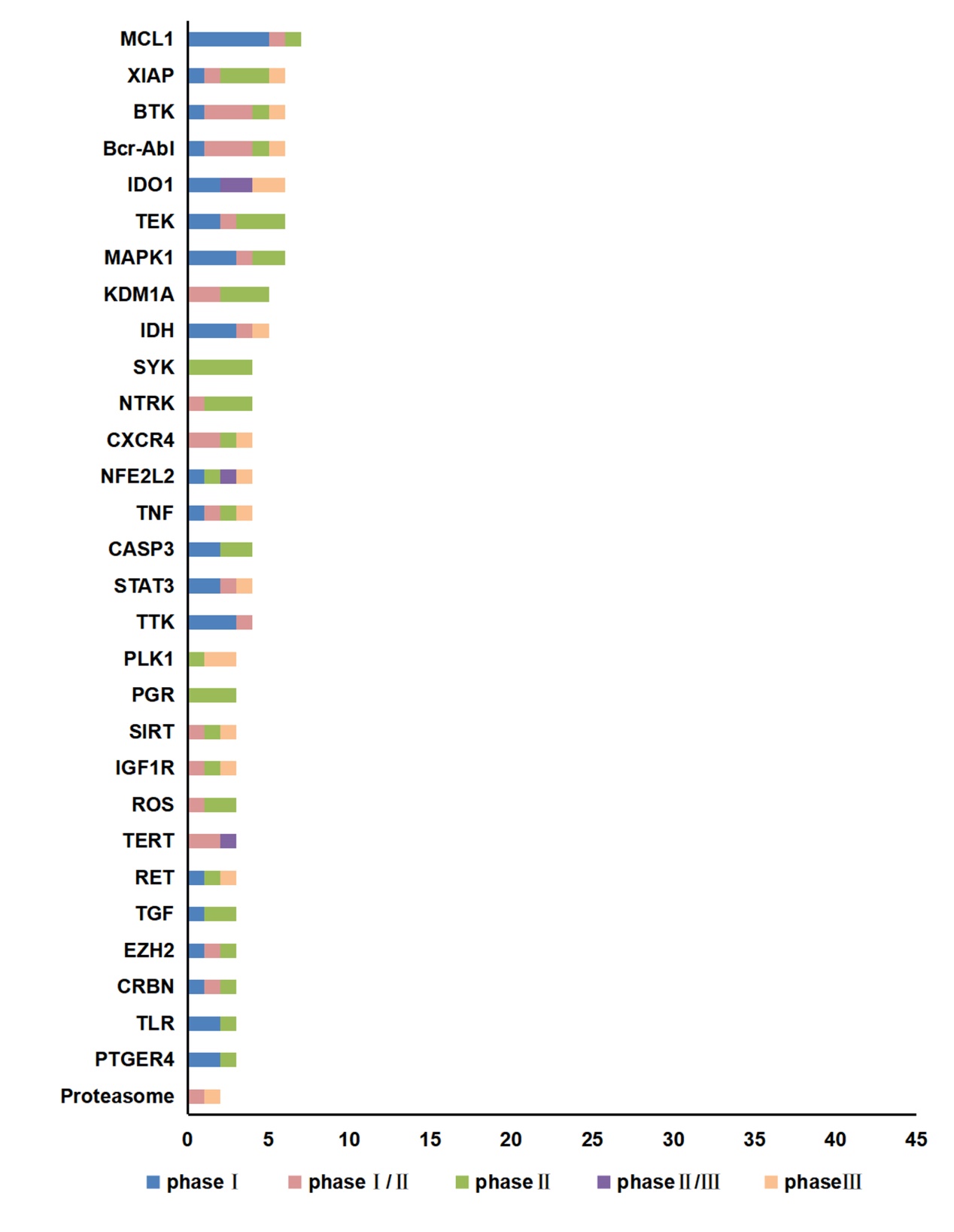


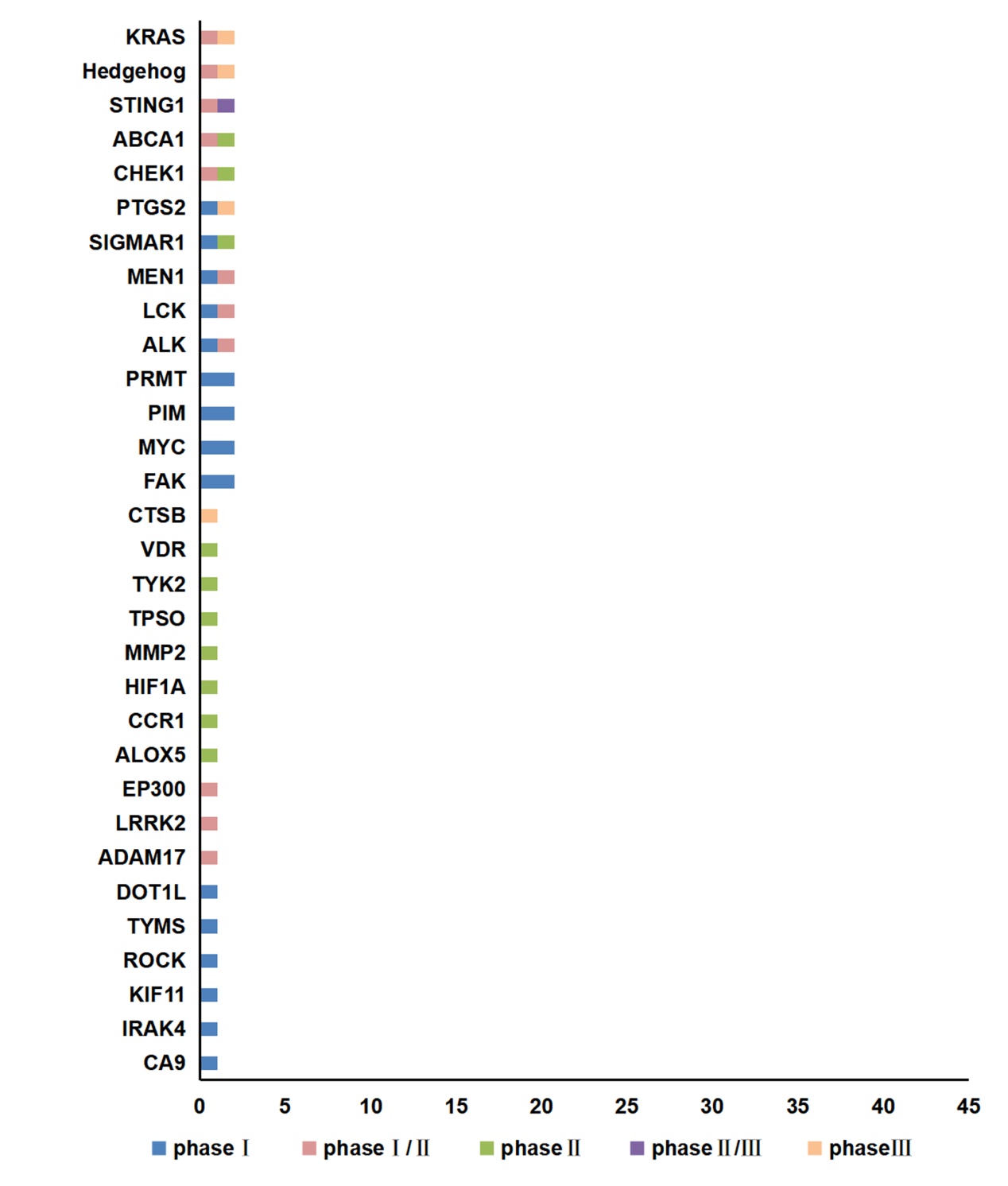

Supplement: Supplementary file 1 — Supplemental Material File #1 [file 41392_2021_572_MOESM1_ESM.docx]
